# Supplementary material for: Enhancing Buprestidae monitoring in Europe: Trap catches increase with a fluorescent yellow colour but not with the presence of decoys
Source: PLoS One. 2024 Jul 18;19(7):e0307397. doi: 10.1371/journal.pone.0307397 (PMC11257278; doi:10.1371/journal.pone.0307397)
Supplement: S1 File — (PDF) [file pone.0307397.s001.pdf]

Agritrap project : Enhancing Buprestidae monitoring in Europe

Supplementary materials of the paper - detailed statistical analyses

Gilles San Martin Alexandre Kuhn & the Agritrap partners

06 December 2023 - 09:29

Contents

1 Introduction **3**

2 General overview of the data **7**
2.1 Variability of total buprestidae captures between sites . . . 7
2.1.1 Raw total catches per site . . . 7
2.1.2 Standardized total per trap and per site . . . 7
2.2 Specific composition . . . 9
2.2.1 Total number of individuals of different species per site . . . 9
2.2.2 Standardized number of individuals per trap and per species on different sites . . . 11
2.3 Specific composition per tree species . . . 11
2.4 Maps . . . 13
2.4.1 World map . . . 13
2.4.2 Belgium . . . 13
2.5 Summary table per site . . . 15
2.6 Conclusions . . . 16

3 Main dataset: Comparison of the effect of different trap types on captures **17**
3.1 Effect of the decoys on the number of catches . . . 17
3.1.1 Do we capture more Buprestidae on comparable traps when we add a decoy ? . . . 17
3.1.2 Are some species more attracted by decoys than others ? . . . 19
3.1.3 Are males or females differently attracted by decoys ? . . . 21
3.1.3.1 Species by species . . . 21
3.1.3.2 All species grouped . . . 23
3.1.4 Conclusions . . . 24
3.2 Effect of trap type on the number of catches . . . 26
3.2.1 What is the effect of trap type on the total number of individuals caught ? . . . 26
3.2.2 What is the relative importance of trap type, site and tree to explain the number of catches ? . . . 29
3.2.3 What is the impact of the area of the trap ? . . . 30
3.2.4 Are some species more attracted by certain types of traps ? . . . 32
3.2.5 Are males or females differently attracted by certain type of traps ? . . . 36
3.2.5.1 Species by species . . . 36
3.2.5.2 All species grouped . . . 39
3.3 Effect of trap type on number of species caught . . . 41
3.3.1 Number of species vs number of individuals or trapping duration . . . 41
3.3.2 Number of species vs trap type . . . 43

3.4 Effect of trap type on the probability of detection of each species 45
3.5 How robust are our conclusions if we change the statistical approach or the data ? 47
3.5.1 Gaussian Mixed Model on a subset of the data with only the complete blocs 47
3.5.1.1 Total number of individuals caught 47
3.5.1.2 Total number of individuals caught without the bottle traps 48
3.5.1.3 Total number of individuals caught and each year analysed separately 49
3.5.2 Poisson/Negative binomial model on a subset of the data with only the complete blocs 50
3.5.2.1 Total number of individuals caught 50
3.5.2.2 Total number of individuals caught without the bottle traps 50
3.5.2.3 Total number of individuals caught and each year analysed separately 51
3.5.3 Trap catches for different species 51
3.5.3.1 Gaussian model on a subset of the data with only the complete blocs 51
3.5.3.2 Poisson / Negative binomial model on a subset of the data with only the complete blocs 53
3.5.4 General conclusions of the robustness analysis 53

4 Examination a few miscellaneous special cases **55**
4.1 “Pale Yellow” Fan-traps 55
4.2 Does (3Z)-hexanol chemical lure have an effect on the captures ? 55
4.3 Captures in Pear trees orchards 56
4.4 Captures on Poplars 57
4.5 Captures in Canada 59

5 Phenology **60**
5.1 Total catches 60
5.2 Phenology of the most common species 61

6 Spectral analysis of the traps color (reflectance spectra) **63**

7 Session Info **65**

8 References **66**

1 Introduction

This document contains the detailed analysis of our "Agritrap" project dataset. The report is entirely generated by R code (using Rmarkdown). If you want see the R code and reproduce our results, please refer to the R script with the same name as this document. All data and R code will be made available on publication in a public figshare repository : https://doi.org/10.6084/m9.figshare.23982834 (DOI activated after publication)

Planed experimental design :

2021 :

- 5 sites per team (only Belgian teams in 2021)
  - PC Fruit : pear Orchards
  - CRA-W : Quercus forests
  - Others = Quercus or Fagus forests/trees
- 3 tree per site with a combination of different traps on the same tree
- 4 type of traps :
  - Bottle trap green
  - Bottle trap green with decoys (most of the time on the same rope as the other bottle trap)
  - MULTz yellow : on 2 trees only
  - Multifunnel green : on 2 trees only

So 2 trees had a combination of 4 traps and 1 tree had only the two types of bottle traps. The multifunnel and MULTz arrived somewhat later in the season and it was not always possible to place them along with the bottle traps at the beginning of the season (however it Was OK for most of the sites with the highest abundance of Agrilus spp.)

2022 :

- 5 sites per team
  - Belgium : same kind of distribution for the 4 Belgian partners
- 2 tree per site with a combination of different traps on the same tree (or very close)
- 6 types of traps :
  - Fan-trap green
  - Fan-trap green with decoys
  - Fan-trap yellow
  - Fan-trap yellow with decoys
  - MULTz yellow
  - Multifunnel green

There has been however some variations around this ideal protocol or some additions.

- Some Fan-traps were not yellow but "pale yellow" (see explanations below) –> these have been excluded from most analyses
- in some localities the samples from fan-traps with or without decoy were pooled together (called "Fan-traps mixed" in the dataset). This was an error made on the field caused by a communication problem between teams and personnel. This means that these samples are the sum of 2 traps and are not directly comparable to the other Fan-Traps. These traps samples were not used to test the effect of the decoys but were used with the other traps once the data from the traps with or without decoys has been pooled.
- ILVO tested a chemical lure on 3 multifunnel traps at 3 sites different sites –> these traps were not used in most analyses
- Some Belgian colleagues put also a few traps in Populus sites to see if it was possible to capture other species like Agrilus ater. The design was often less structured for these sites –> these data have been

excluded from most analyses.

- We sent Fan-Traps in Canada (team = RNCan) and France (Team = INRAE) where colleagues placed the fan-traps of different colors with or without decoys along with a variation of other traps commercial traps
- The French colleagues added some 3D decoys on some of the mutifunnel green traps. These traps have been excluded from most analyses because their low number made them not usable for global comparisons The decoys used on the Fan-Traps and Bottle traps where all dead A.planipennis

There were also some deviations from the planed design due to field or organisational constraints (some traps were destroyed or detached by the wind, some traps models were available later than expected, it was impossible to replace some traps when our stock was depleted, etc.).

NB: for the main statistical analyses and graphs, if a species has never been observed on a given tree in any of the traps, then the lines corresponding to this combination of treeID and burpestid species are removed from the dataset. This allows us to ignore the cases were a species is absent (the comparison between traps is useless in thi case). This means also that when we analyse the total number of buprestid caught, the trees with 0 captures are dropped.

The following table provides a summary of all the combinations of treatments, including the unusual variations that were most of the time excluded from the analyses.
`NbIndiv` = total number of individuals captured, `NbSites` = number of sites `NbTraps` = Number of traps, `NbSamples` Number of samples (a sample is one catch in one trap during a certain period of time)

Year	Team	Tree_Species	Trap_Model	Trap_Color	Trap_Decoy	Trap_Lure	NbIndiv	NbSites	NbTraps	NbSamples
2021	CRAW	Quercus	Bottle	Green	A.planipennis	No lure	63	5	15	104
2021	CRAW	Quercus	Bottle	Green	No decoy	No lure	47	5	15	103
2021	CRAW	Quercus	Multifunnel	Green	No decoy	No lure	73	5	10	70
2021	CRAW	Quercus	MULTz	Yellow	No decoy	No lure	600	5	10	67
2021	PCfruit	Pyrus	Bottle	Green	A.planipennis	No lure	2	5	15	135
2021	PCfruit	Pyrus	Bottle	Green	No decoy	No lure	5	5	15	135
2021	PCfruit	Pyrus	Multifunnel	Green	No decoy	No lure	4	5	10	40
2021	PCfruit	Pyrus	MULTz	Yellow	No decoy	No lure	6	5	10	80
2021	ULB	Fagus	Bottle	Green	A.planipennis	No lure	0	2	6	30
2021	ULB	Fagus	Bottle	Green	No decoy	No lure	0	2	6	30
2021	ULB	Fagus	Multifunnel	Green	No decoy	No lure	1	2	2	4
2021	ULB	Fagus	MULTz	Yellow	No decoy	No lure	1	2	5	20
2021	ULB	Quercus	Bottle	Green	A.planipennis	No lure	0	1	3	15
2021	ULB	Quercus	Bottle	Green	No decoy	No lure	1	1	3	15
2021	ULB	Quercus	Multifunnel	Green	No decoy	No lure	0	1	1	2
2021	ULB	Quercus	MULTz	Yellow	No decoy	No lure	5	1	2	10
2022	CRAW	Populus	Multifunnel	Green	No decoy	No lure	6	8	8	19
2022	CRAW	Populus	MULTz	Yellow	No decoy	No lure	2	6	6	11
2022	CRAW	Quercus	Fan-trap	Green	A.planipennis	No lure	114	5	10	78
2022	CRAW	Quercus	Fan-trap	Green	No decoy	No lure	132	5	10	77
2022	CRAW	Quercus	Fan-trap	Yellow	A.planipennis	No lure	668	5	10	78
2022	CRAW	Quercus	Fan-trap	Yellow	No decoy	No lure	593	5	10	79
2022	CRAW	Quercus	Multifunnel	Green	No decoy	No lure	499	5	10	77
2022	CRAW	Quercus	MULTz	Yellow	No decoy	No lure	1370	5	10	78
2022	ILVO	Fagus	Fan-trap	Green	Mixed	No lure	0	1	1	3
2022	ILVO	Fagus	Fan-trap	Yellow	Mixed	No lure	0	1	1	3
2022	ILVO	Fagus	Multifunnel	Green	No decoy	No lure	0	1	1	4
2022	ILVO	Fagus	MULTz	Yellow	No decoy	No lure	0	1	1	4
2022	ILVO	Populus	Fan-trap	Green	Mixed	No lure	0	1	2	6
2022	ILVO	Populus	Fan-trap	Yellow	Mixed	No lure	5	1	2	6
2022	ILVO	Populus	Multifunnel	Green	No decoy	No lure	112	1	3	9
2022	ILVO	Populus	MULTz	Yellow	No decoy	No lure	24	1	3	9
2022	ILVO	Quercus	Fan-trap	Green	Mixed	No lure	4	2	4	12
2022	ILVO	Quercus	Fan-trap	Yellow	Mixed	No lure	14	2	4	12
2022	ILVO	Quercus	Multifunnel	Green	No decoy	(3Z)-hexanol	14	3	3	12
2022	ILVO	Quercus	Multifunnel	Green	No decoy	No lure	60	6	9	34
2022	ILVO	Quercus	MULTz	Yellow	No decoy	No lure	30	3	6	22
2022	PCfruit	Pyrus	Fan-trap	Green	A.planipennis	No lure	0	4	9	45
2022	PCfruit	Pyrus	Fan-trap	Green	No decoy	No lure	0	4	9	45

Year	Team	Tree_Species	Trap_Model	Trap_Color	Trap_Decoy	Trap_Lure	NbIndiv	NbSites	NbTraps	NbSamples
2022	PCfruit	Pyrus	Fan-trap	Yellow	A.planipennis	No lure	0	4	9	45
2022	PCfruit	Pyrus	Fan-trap	Yellow	No decoy	No lure	0	4	9	45
2022	PCfruit	Pyrus	Multifunnel	Green	No decoy	No lure	0	4	9	45
2022	PCfruit	Pyrus	MULTz	Yellow	No decoy	No lure	0	3	6	30
2022	ULB	Fagus	Fan-trap	Green	A.planipennis	No lure	1	3	6	48
2022	ULB	Fagus	Fan-trap	Green	No decoy	No lure	1	3	6	47
2022	ULB	Fagus	Fan-trap	Pale yellow	A.planipennis	No lure	1	3	6	47
2022	ULB	Fagus	Fan-trap	Pale yellow	No decoy	No lure	2	3	6	47
2022	ULB	Fagus	Fan-trap	Yellow	A.planipennis	No lure	2	3	6	36
2022	ULB	Fagus	Fan-trap	Yellow	No decoy	No lure	4	3	6	36
2022	ULB	Fagus	Multifunnel	Green	No decoy	No lure	24	3	6	47
2022	ULB	Fagus	MULTz	Yellow	No decoy	No lure	1	3	5	37
2022	ULB	Quercus	Fan-trap	Green	A.planipennis	No lure	0	2	4	32
2022	ULB	Quercus	Fan-trap	Green	No decoy	No lure	1	2	4	32
2022	ULB	Quercus	Fan-trap	Pale yellow	A.planipennis	No lure	0	2	4	32
2022	ULB	Quercus	Fan-trap	Pale yellow	No decoy	No lure	0	2	4	32
2022	ULB	Quercus	Fan-trap	Yellow	A.planipennis	No lure	1	2	4	24
2022	ULB	Quercus	Fan-trap	Yellow	No decoy	No lure	1	2	4	24
2022	ULB	Quercus	Multifunnel	Green	No decoy	No lure	4	2	4	32
2022	ULB	Quercus	MULTz	Yellow	No decoy	No lure	1	2	4	32
2022	RNCan	Quercus	Fan-trap	Green	A.planipennis	No lure	1	1	3	9
2022	RNCan	Quercus	Fan-trap	Green	No decoy	No lure	0	1	3	9
2022	RNCan	Quercus	Fan-trap	Yellow	A.planipennis	No lure	3	1	3	9
2022	RNCan	Quercus	Fan-trap	Yellow	No decoy	No lure	14	1	3	9
2022	RNCan	Quercus	Multifunnel	Green	No decoy	No lure	15	1	3	9
2022	INRAE	Quercus	Fan-trap	Green	A.planipennis	No lure	3	6	6	12
2022	INRAE	Quercus	Fan-trap	Green	No decoy	No lure	12	6	6	12
2022	INRAE	Quercus	Fan-trap	Yellow	A.planipennis	No lure	32	6	6	12
2022	INRAE	Quercus	Fan-trap	Yellow	No decoy	No lure	56	6	6	12
2022	INRAE	Quercus	Multifunnel	Green	3D commercial	No lure	259	6	6	12
2022	INRAE	Quercus	Multifunnel	Green	3D INRAE	No lure	179	6	6	12
2022	INRAE	Quercus	Multifunnel	Green	No decoy	No lure	72	6	6	11
2022	INRAE	Quercus	MULTz	Yellow	No decoy	No lure	166	6	6	10

Another summary table showing only the main sites used in most analysis (eg excluding sites with 3Z lures, pale yellow traps, samples from Canada etc…) and grouping per country, year, tree species and type of trap. NbDays = average number of days each trap remained active on the field.

Country	Year	Tree_Species	Trap_Type	NbIndiv	NbSites	NbTraps	NbSamples	NbDays
Belgium	2021	Fagus	Bottle green	0	2	6	30	107.0
Belgium	2021	Fagus	Bottle green decoy	0	2	6	30	107.0
Belgium	2021	Fagus	Multifunnel green	1	2	2	4	37.0
Belgium	2021	Fagus	MULTz yellow	1	2	5	20	89.2
Belgium	2021	Pyrus	Bottle green	5	5	15	135	101.0
Belgium	2021	Pyrus	Bottle green decoy	2	5	15	135	101.0
Belgium	2021	Pyrus	Multifunnel green	4	5	10	40	38.0
Belgium	2021	Pyrus	MULTz yellow	6	5	10	80	89.8
Belgium	2021	Quercus	Bottle green	48	6	18	118	100.5
Belgium	2021	Quercus	Bottle green decoy	63	6	18	119	101.3
Belgium	2021	Quercus	Multifunnel green	73	6	11	72	91.7
Belgium	2021	Quercus	MULTz yellow	605	6	12	77	98.2
Belgium	2022	Fagus	Fan-trap green	1	3	6	47	122.8
Belgium	2022	Fagus	Fan-trap green decoy	1	3	6	48	125.0
Belgium	2022	Fagus	Fan-trap green mixed	0	1	1	3	89.0
Belgium	2022	Fagus	Fan-trap yellow	4	3	6	36	90.0
Belgium	2022	Fagus	Fan-trap yellow decoy	2	3	6	36	90.0
Belgium	2022	Fagus	Fan-trap yellow mixed	0	1	1	3	89.0
Belgium	2022	Fagus	Multifunnel green	24	4	7	51	122.2
Belgium	2022	Fagus	MULTz yellow	1	4	6	41	118.0
Belgium	2022	Populus	Fan-trap green mixed	0	1	2	6	89.0
Belgium	2022	Populus	Fan-trap yellow mixed	5	1	2	6	89.0
Belgium	2022	Populus	Multifunnel green	118	9	11	28	55.1
Belgium	2022	Populus	MULTz yellow	26	7	9	20	48.8
Belgium	2022	Pyrus	Fan-trap green	0	4	9	45	84.0
Belgium	2022	Pyrus	Fan-trap green decoy	0	4	9	45	84.0
Belgium	2022	Pyrus	Fan-trap yellow	0	4	9	45	84.0
Belgium	2022	Pyrus	Fan-trap yellow decoy	0	4	9	45	84.0
Belgium	2022	Pyrus	Multifunnel green	0	4	9	45	84.0
Belgium	2022	Pyrus	MULTz yellow	0	3	6	30	84.0
Belgium	2022	Quercus	Fan-trap green	133	7	14	109	113.5
Belgium	2022	Quercus	Fan-trap green decoy	114	7	14	110	114.4
Belgium	2022	Quercus	Fan-trap green mixed	4	2	4	12	90.5
Belgium	2022	Quercus	Fan-trap yellow	594	7	14	103	105.2
Belgium	2022	Quercus	Fan-trap yellow decoy	669	7	14	102	104.2
Belgium	2022	Quercus	Fan-trap yellow mixed	14	2	4	12	90.5
Belgium	2022	Quercus	Multifunnel green	554	10	20	131	114.9
Belgium	2022	Quercus	MULTz yellow	1401	10	20	132	115.7
France	2022	Quercus	Fan-trap green	12	6	6	12	44.0
France	2022	Quercus	Fan-trap green decoy	3	6	6	12	44.0
France	2022	Quercus	Fan-trap yellow	56	6	6	12	44.0
France	2022	Quercus	Fan-trap yellow decoy	32	6	6	12	44.0
France	2022	Quercus	Multifunnel green	72	6	6	11	53.2
France	2022	Quercus	MULTz yellow	166	6	6	10	48.3

2 General overview of the data

2.1 Variability of total buprestidae captures between sites

NB : the "Vierzon" sites are from France and "Cranberry Lake" is the Canadian site.

2.1.1 Raw total catches per site

The following graph shows simply the sum of all individuals per site (without taking into account differences in trapping intensity). NB : Rochefort is the only site that has been sampled during the 2 years.

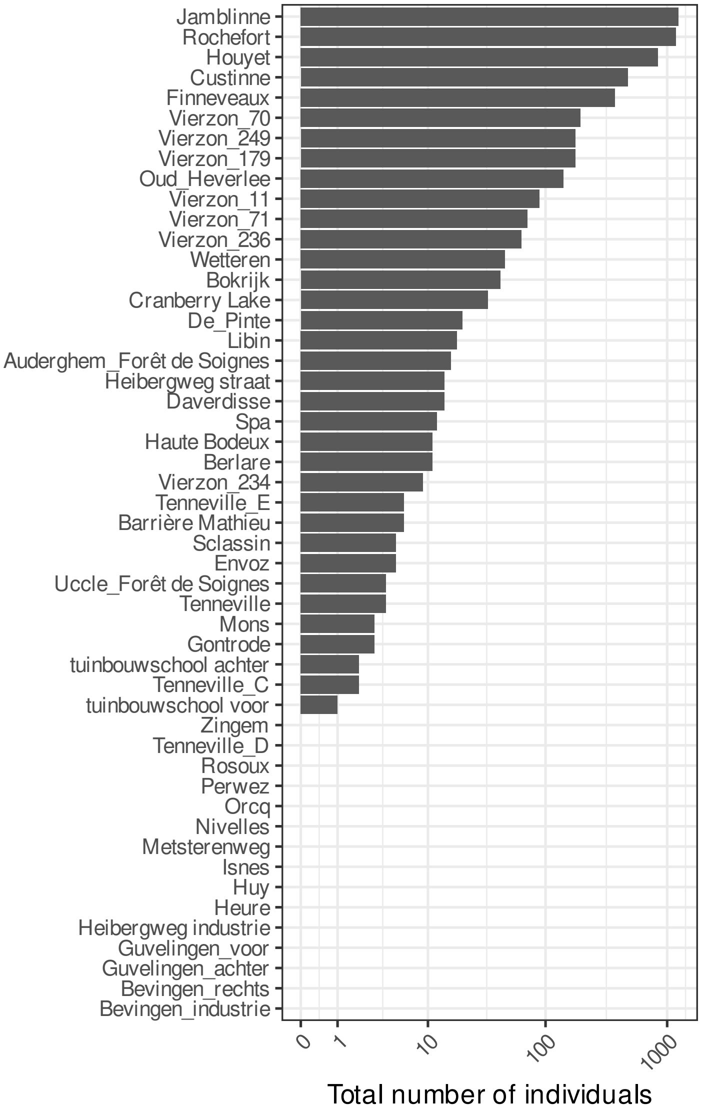

Figure 1:

2.1.2 Standardized total per trap and per site

Each point represent the captures in one trap standardized for a trapping duration of 90 days. The red dot and bars are the average and 95% bootstrap Confidence Interval. The scale is logarithmic !

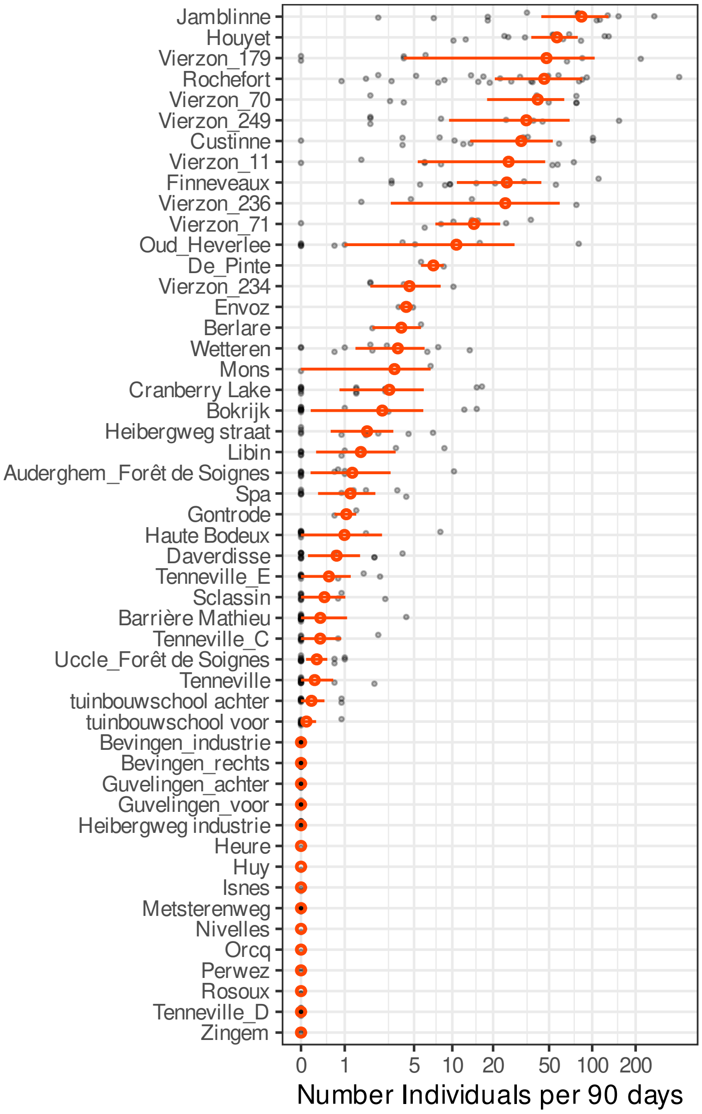

Figure 2:

Conclusions

There is a very large variation between sites. In some sites we captured almost no Buprestidae probably because there were very few of them in that site. For pear orchards where the only captured species is Agrilus sinuatus, it is possible that this species in particular is not very much attracted by the traps.

It will be difficult to compare the traps types from sites where we had so few captures.

2.2 Specific composition

Full names of the Species

On request of one of the reviewers, we provide here the full names of the species, with their authority, as retrieved from the GBIF backbone taxonomy (through the `taxize` package).

Names	Full_Names
Agrilus angustulus	Agrilus angustulus (Illiger, 1803)
Agrilus ater	Agrilus ater (Linnaeus, 1767)
Agrilus biguttatus	Agrilus biguttatus (Fabricius, 1776)
Agrilus bilineatus	Agrilus bilineatus (Weber, 1801)
Agrilus convexicollis	Agrilus convexicollis Redtenbacher, 1849
Agrilus crinicornis	Agrilus crinicornis Horn, 1891
Agrilus curtulus	Agrilus curtulus Mulsant & Rey, 1863
Agrilus graminis	Agrilus graminis Kiesenwetter, 1857
Agrilus hastulifer	Agrilus hastulifer (Ratzeburg, 1837)
Agrilus laticornis	Agrilus laticornis (Illiger, 1803)
Agrilus obscuricollis	Agrilus obscuricollis Kiesenwetter, 1857
Agrilus obsoletoguttatus	Agrilus obsoletoguttatus Gory, 1841
Agrilus olivicolor	Agrilus olivicolor Kiesenwetter, 1857
Agrilus pratensis	Agrilus pratensis (Ratzeburg, 1837)
Agrilus sinuatus	Agrilus sinuatus (Olivier, 1790)
Agrilus spp.	Agrilus Curtis, 1825
Agrilus sulcicollis	Agrilus sulcicollis Lacordaire, 1835
Agrilus viridis	Agrilus viridis (Linnaeus, 1758)
Anthaxia candens	Anthaxia candens (Panzer, 1793)
Anthaxia manca	Anthaxia manca (Linnaeus, 1767)
Anthaxia nitidula	Anthaxia nitidula (Linnaeus, 1758)
Anthaxia quadripunctata	Anthaxia quadripunctata (Linnaeus, 1758)
Anthaxia quercata	Anthaxia quercata (Fabricius, 1801)
Anthaxia salicis	Anthaxia salicis (Fabricius, 1776)
Brachys aerosus	Brachys aerosus Melsheimer, 1845
Chrysobothris affinis	Chrysobothris affinis (Fabricius, 1794)
Coraebus undatus	Coraebus undatus (Fabricius, 1787)
Meliboeus fulgidicollis	Meliboeus fulgidicollis (Lucas, 1846)
Trachys minutus	Trachys minutus (Linnaeus, 1758)
Quercus	Quercus L.
Fagus	Fagus L.
Populus	Populus L.
Pyrus	Pyrus Webster, 1814

2.2.1 Total number of individuals of different species per site

Comparison of the specific composition between countries. The Canadian fauna is of course completely different.

The French fauna is rather similar with a few additional species (A. curtulus, A. hastulifer,...). Agrilus sulcicollis seems to be much more frequent in Belgium.

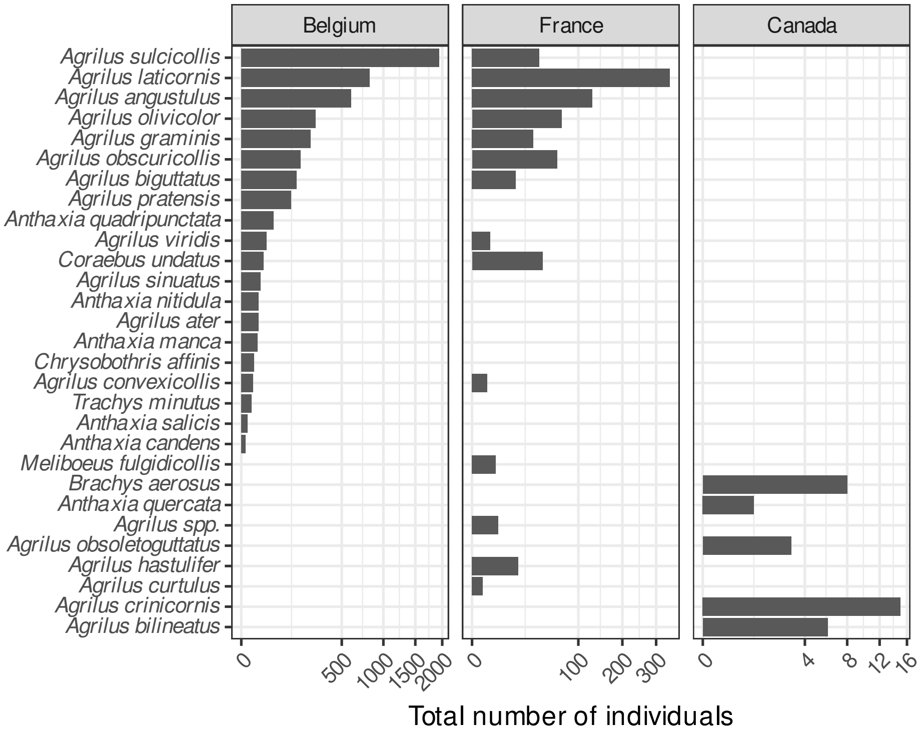

Figure 3:

Same graph with only the European sites/fauna :

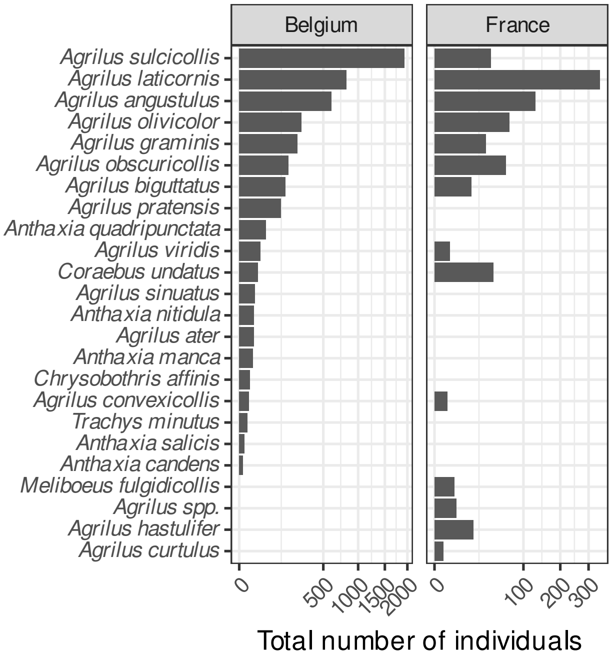

Figure 4:

2.2.2 Standardized number of individuals per trap and per species on different sites

Each point represent the number of individuals of a given species captured in a given trap (all trap types mixed).

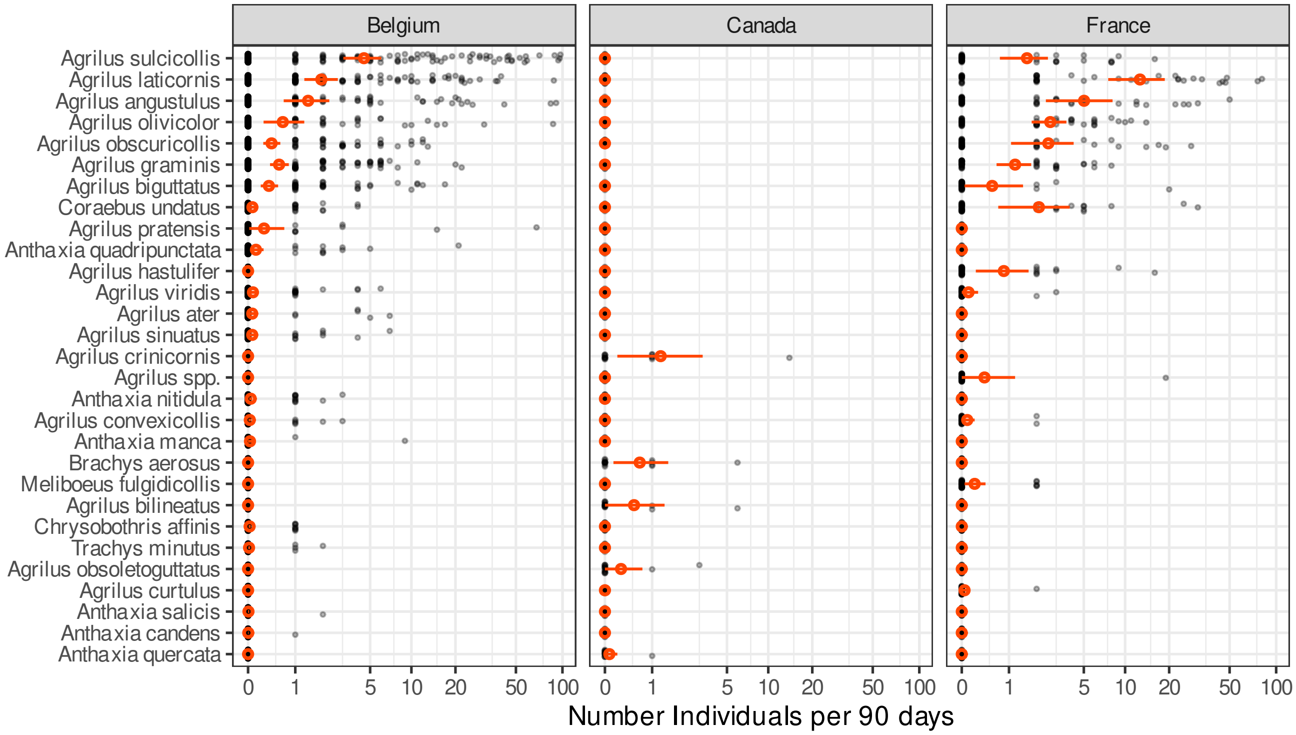

Figure 5:

2.3 Specific composition per tree species

NB : the sampling effort is different between tree species. But the relative abundance can be compared between buprestid species within a given tree species.

Same graph with only the European sites/fauna :

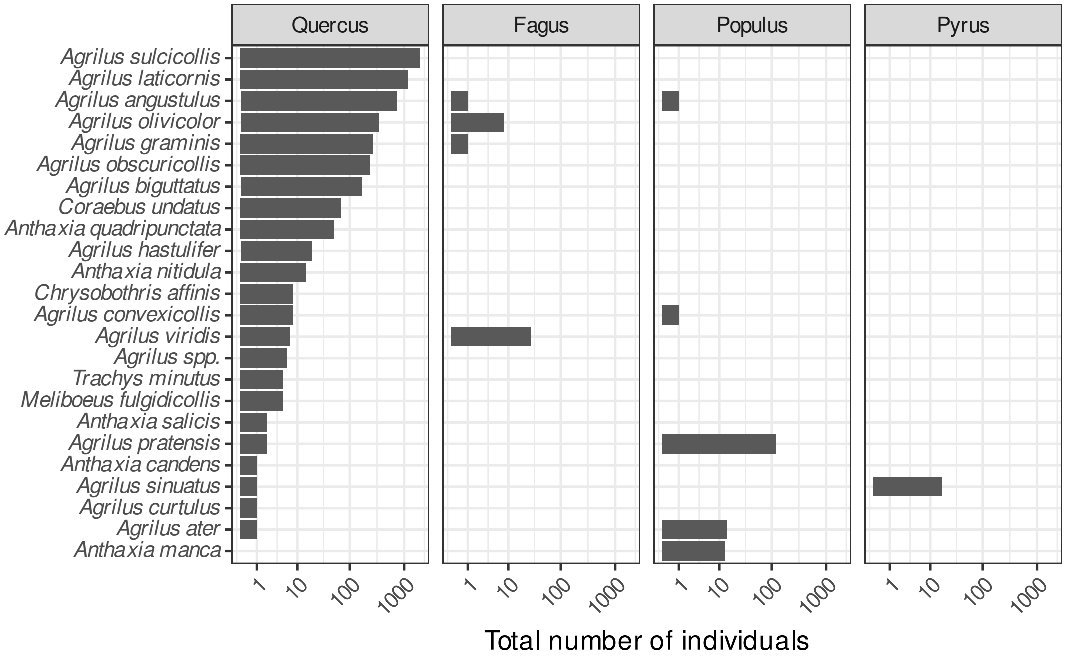
Quercus
Fagus
Populus
Pyrus
Agrilus sulcicollis
Agrilus laticornis
Agrilus angustulus
Agrilus olivicolor
Agrilus graminis
Agrilus obscuricollis
Agrilus biguttatus
Coraebus undatus
Anthaxia quadripunctata
Agrilus hastulifer
Anthaxia nitidula
Chrysobothris affinis
Agrilus convexicollis
Agrilus viridis
Agrilus spp.
Trachys minutus
Meliboeus fulgidicollis
Anthaxia salicis
Agrilus pratensis
Anthaxia candens
Agrilus sinuatus
Agrilus curtulus
Agrilus ater
Anthaxia manca
1
10
100
1000
Total number of individuals

Figure 6:

2.4 Maps

2.4.1 World map

Position of the sites in Belgium, France and Canada

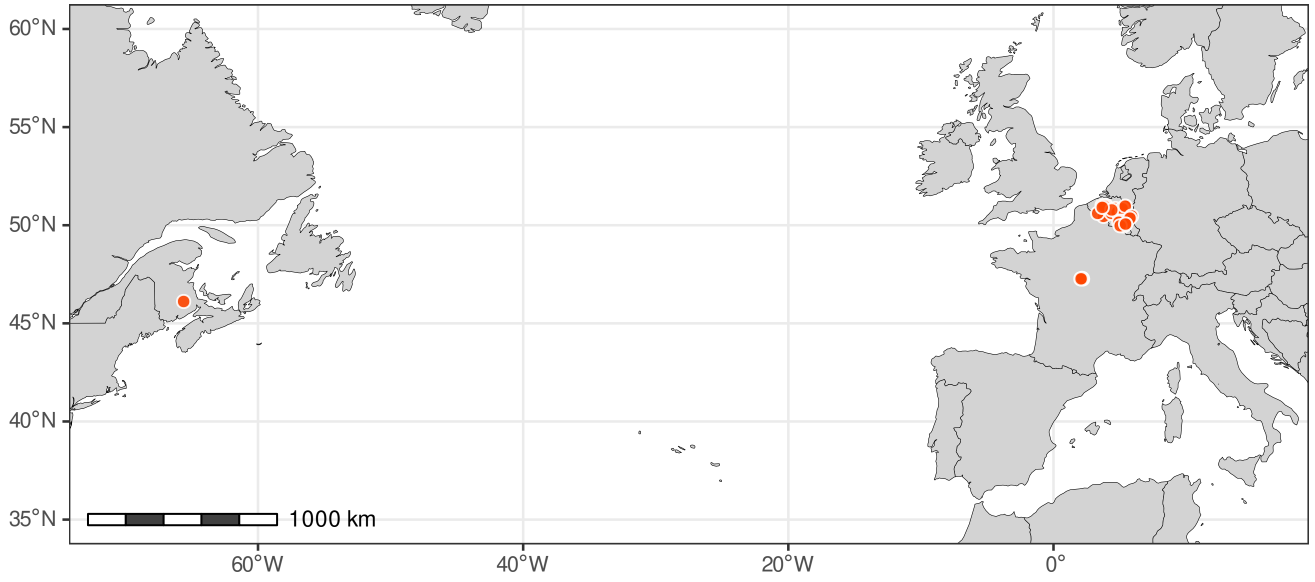

Figure 7:

2.4.2 Belgium

Map showing the distribution of the sites in Belgium for each tree species. All French sites were situated in 6 sites in the Vierzon forest and the Canadian samples came all from the same site near Cranberry Lake.

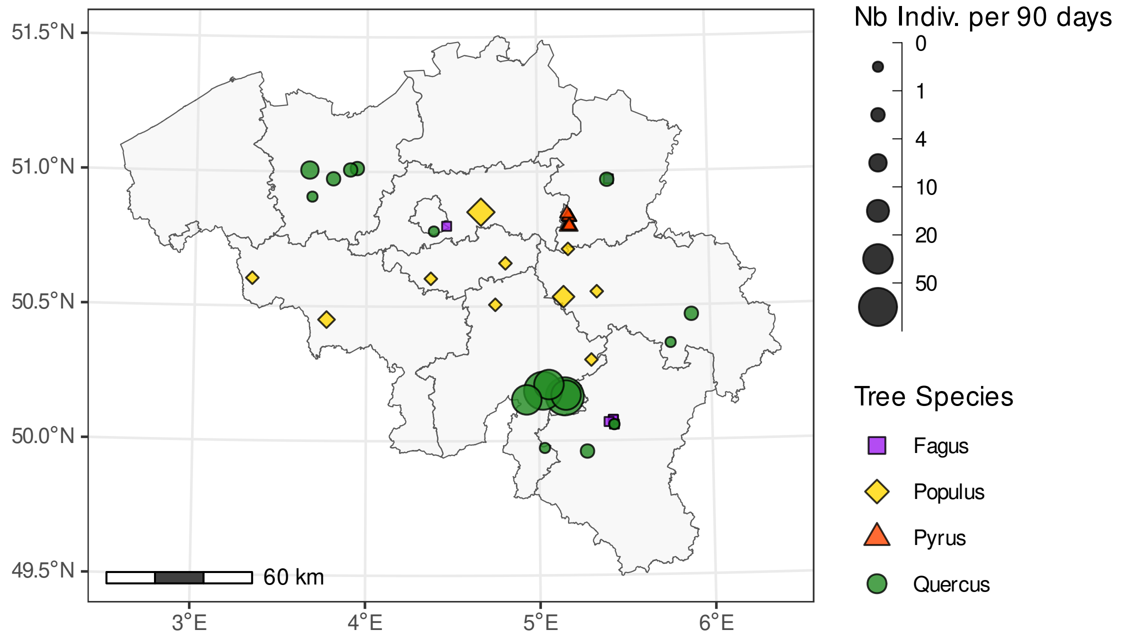

Figure 8:

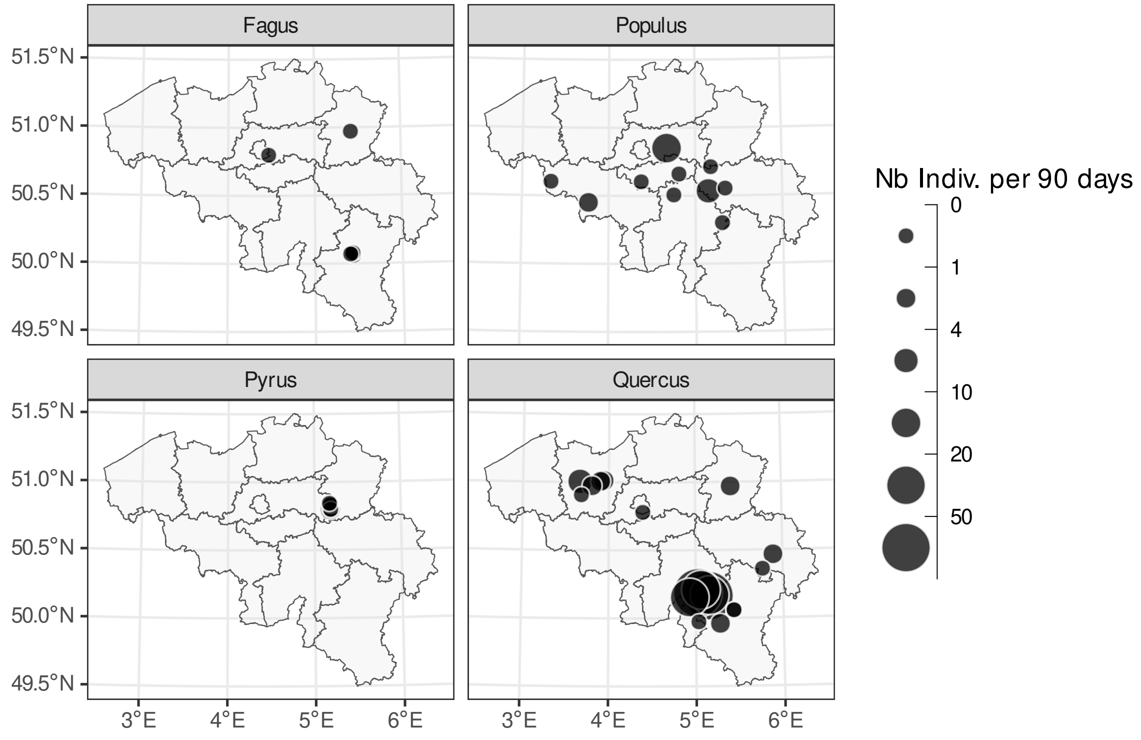

Figure 9:

2.5 Summary table per site

the following table summarizes the data per site used to create these maps.

Country	SiteID	Tree	Total	NbDays	Nb90Days	Date_Start	Date_End	Long	Lat
Belgium	Auderghem_Forêt de Soignes	Fagus	16	112.6	0.92	2022-05-10	2022-09-12	4.468	50.799
Belgium	Daverdisse	Fagus	14	113.4	0.71	2022-05-11	2022-09-13	5.434	50.059
Belgium	Tenneville_C	Fagus	2	99.2	0.36	2021-06-08	2021-09-23	5.429	50.077
Belgium	Barrière Mathieu	Fagus	6	115.4	0.27	2022-05-11	2022-09-13	5.429	50.077
Belgium	Bokrijk	Fagus	0	106.5	0.00	2022-05-04	2022-09-05	5.419	50.970
Belgium	Tenneville_D	Fagus	0	89.3	0.00	2021-06-08	2021-09-23	5.404	50.069
Belgium	Oud_Heverlee	Populus	141	92.2	10.75	2022-05-04	2022-09-05	4.669	50.851
Belgium	Envoz	Populus	5	55.0	4.28	2022-05-04	2022-07-16	5.148	50.535
Belgium	Mons	Populus	3	40.0	3.38	2022-07-22	2022-08-31	3.768	50.451
Belgium	Heure	Populus	0	35.0	0.00	2022-07-27	2022-08-31	5.307	50.300
Belgium	Huy	Populus	0	35.0	0.00	2022-07-27	2022-08-31	5.342	50.553
Belgium	Isnes	Populus	0	56.0	0.00	2022-05-18	2022-07-20	4.751	50.506
Belgium	Nivelles	Populus	0	51.0	0.00	2022-05-18	2022-07-08	4.375	50.604
Belgium	Orcq	Populus	0	40.0	0.00	2022-07-22	2022-08-31	3.333	50.603
Belgium	Perwez	Populus	0	44.0	0.00	2022-05-18	2022-07-08	4.811	50.660
Belgium	Rosoux	Populus	0	49.0	0.00	2022-05-04	2022-06-22	5.177	50.712
Belgium	Heibergweg straat	Pyrus	14	88.4	1.84	2021-06-07	2021-09-16	5.183	50.798
Belgium	tuinbouwschool achter	Pyrus	2	88.4	0.18	2021-06-07	2021-09-16	5.189	50.831
Belgium	tuinbouwschool voor	Pyrus	1	88.4	0.09	2021-06-07	2021-09-16	5.183	50.833
Belgium	Bevingen_industrie	Pyrus	0	84.0	0.00	2022-06-03	2022-08-26	5.184	50.800
Belgium	Bevingen_rechts	Pyrus	0	84.0	0.00	2022-06-03	2022-08-26	5.184	50.800
Belgium	Guvelingen_achter	Pyrus	0	84.0	0.00	2022-06-03	2022-08-26	5.189	50.830
Belgium	Guvelingen_voor	Pyrus	0	84.0	0.00	2022-06-03	2022-08-26	5.183	50.833
Belgium	Heibergweg industrie	Pyrus	0	77.2	0.00	2021-06-07	2021-09-16	5.187	50.798
Belgium	Metsterenweg	Pyrus	0	88.4	0.00	2021-06-07	2021-09-16	5.174	50.843
Belgium	Jamblinne	Quercus	1243	110.8	84.23	2022-05-12	2022-09-02	5.150	50.165
Belgium	Houyet	Quercus	843	109.0	56.75	2022-05-13	2022-09-02	5.024	50.186
Belgium	Rochefort	Quercus	1179	104.1	46.22	2021-06-04	2022-09-02	5.157	50.169
Belgium	Custinne	Quercus	475	112.0	31.81	2022-05-13	2022-09-02	5.059	50.209
Belgium	Finneveaux	Quercus	373	111.9	25.08	2022-05-12	2022-09-02	4.930	50.153
Belgium	De_Pinte	Quercus	20	126.0	7.14	2022-05-06	2022-09-09	3.663	51.006
Belgium	Bokrijk	Quercus	42	106.5	3.95	2022-05-04	2022-09-05	5.410	50.969
Belgium	Berlare	Quercus	11	127.0	3.90	2022-05-05	2022-09-09	3.943	51.012
Belgium	Wetteren	Quercus	46	100.2	3.64	2022-05-05	2022-09-09	3.903	51.007
Belgium	Libin	Quercus	18	102.4	1.57	2021-06-04	2021-09-16	5.277	49.960
Belgium	Spa	Quercus	12	92.2	1.19	2021-06-07	2021-09-15	5.891	50.466
Belgium	Gontrode	Quercus	3	127.0	1.06	2022-05-05	2022-09-09	3.803	50.974
Belgium	Haute Bodeux	Quercus	11	98.6	0.99	2021-06-07	2021-09-15	5.768	50.361
Belgium	Tenneville_E	Quercus	6	99.2	0.56	2021-06-08	2021-09-23	5.434	50.059
Belgium	Sclassin	Quercus	5	102.2	0.44	2021-05-20	2021-09-16	5.032	49.973
Belgium	Uccle_Forêt de Soignes	Quercus	4	116.2	0.22	2022-05-10	2022-09-12	4.393	50.779
Belgium	Tenneville	Quercus	4	116.2	0.18	2022-05-11	2022-09-13	5.434	50.059
Belgium	Zingem	Quercus	0	126.0	0.00	2022-05-06	2022-09-09	3.680	50.907
Canada	Cranberry Lake	Quercus	33	65.0	3.05	2022-07-04	2022-09-07	-65.610	46.113
France	Vierzon_179	Quercus	176	47.4	47.96	2022-05-31	2022-07-28	2.119	47.266
France	Vierzon_70	Quercus	195	51.0	41.59	2022-05-31	2022-07-28	2.155	47.288
France	Vierzon_249	Quercus	176	51.0	34.57	2022-05-31	2022-07-28	2.068	47.265
France	Vierzon_11	Quercus	89	43.8	25.83	2022-05-31	2022-07-28	2.193	47.260
France	Vierzon_236	Quercus	63	58.0	24.44	2022-05-31	2022-07-28	2.081	47.269
France	Vierzon_71	Quercus	71	51.0	14.51	2022-05-31	2022-07-28	2.155	47.288
France	Vierzon_234	Quercus	9	44.0	4.60	2022-06-14	2022-07-28	2.081	47.269

2.6 Conclusions

Because most species have been captured in very low quantities or in very few sites, most data items are "0" values and it will be difficult to exploit the data from these rare species (excepted in terms of species diversity).

We should probably limit ourselves to the 7 most common Agrilus species (all oak species) when we want a species by species analysis : "Agrilus sulcicollis", "Agrilus laticornis", "Agrilus angustulus", "Agrilus olivicolor", "Agrilus graminis", "Agrilus obscuricollis", "Agrilus biguttatus"

Overall, we captured 4814 Buprestidae specimens, including 4669 of the genus Agrilus. We collected 2220 samples from 382 traps spread over 46 sites in France and Belgium. On average, each trap remained active for 92.2 days. Most captures took place in June - July and declined in August. The number of catches varied a lot between sites (range of the number of Buprestidae captured per trap per 90 days: 0 to 400, median = 0, average = 11.6). Over the two years of study, the traps captured 23 different species (20 in Belgium and 12 in France, where the sampling effort was lower). The seven most abundant species (A.sulcicollis, A.laticornis, A.angustulus, A.olivicolor, A.graminis, A.obscuricollis, A.biguttatus) accounted for 92.7% of the total catches. In Belgium, the most abundant species was Agrilus sulcicollis with 1925 specimens captured (5.2 specimens/trap/90 days). This species occurred much less frequently in France (ranking 6th with 19 specimens captured, i.e. 1.03 specimens/trap/90 days). In France, A. laticornis was the most frequent species with 123 specimens (5.2 specimens/trap/90 days). The highest number of species was found in oak stands (21 species), while we found only Agrilus sinuatus in pear orchards. The catches were particularly poor in pear orchards: we only captured 17 specimens of Agrilus sinuatus in 2021 and no specimen at all in 2022 despite a confirmed presence by beating. For oak stands, the fauna from Belgium and France was quite similar, with a few species found only in traps from France (A. hastulifer, A. curtulus, Meliboeus fulgidicollis).

3 Main dataset: Comparison of the effect of different trap types on captures

3.1 Effect of the decoys on the number of catches

For 3 type of traps (bottle green, Fan-trap green, Fan-trap yellow), we had systematically the same trap at the same position (hanging on top of each other) with or without a decoy composed of a dead Agrilus planipennis.

However in certain sites the catches from the traps with or without decoy have been pooled and cannot be compared with the others.

This is why we are going to test here if there is an effect of the presence of the decoys. If not we will pool the catches of the same traps types with or without decoys for all sites.

The area of the bottle and Fan-traps being much smaller than the area of the multifunnel and MULTz the resulting comparisons will probably be more "fair"

3.1.1 Do we capture more Buprestidae on comparable traps when we add a decoy ?

On the following graph the gray lines represent the total captures of a pair of similar traps from the same tree with or without decoy. The number of individual has been standardized to have a comparable number of trapping days (90 days). The red dots represent the average and the bars represent the 95% confidence interval (bootstrap).

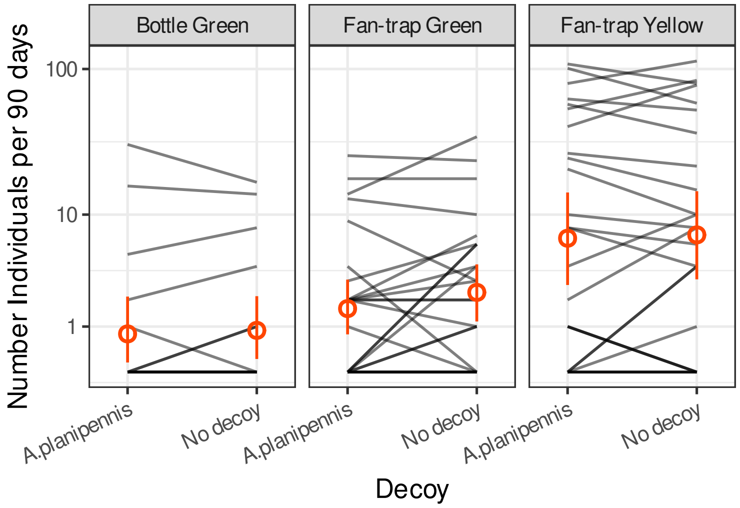

Figure 10:

We can formally test the hypothesis of differences between traps with or without decoy (controlling for the type of Trap and their interaction + 2 random effects : Site and Tree) :

We use a linear mixed model with the log(x+1) transformed number of catches * 90 days / nbr of Days. ie we use the corrected number of catches per trap.

```
lmer(log1p(Nb_Indiv_90_Days) ~ Trap * Trap_Decoy + (1|SiteID) + (1|TreeID), data = tmp)
```

The residual plots of this model looks very coherent with a gaussian model.

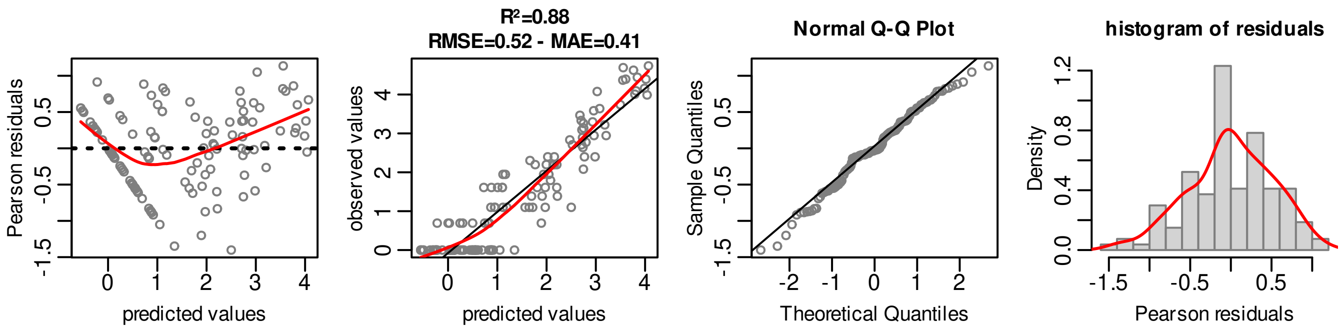

Figure 11:

The Anova table shows no interaction trap x decoy nor any main decoy effect while the trap has a higly significant effect

Table 5: Analysis of Deviance Table (Type II Wald F tests with Kenward-Roger df)

	F	Df	Df.res	Pr(>F)
**Trap**	26.42	2	77.77	0.000000
**Trap_Decoy**	1.37	1	82.54	0.244584
**Trap:Trap_Decoy**	0.39	2	82.54	0.677462

Note in the summary the high value for the random component of the Site : there is a large between site variation...

```
## Linear mixed model fit by REML ['lmerMod']
## Formula: log1p(Nb_Indiv_90_Days) ~ Trap * Trap_Decoy + (1 | SiteID) +      (1 | TreeID)
##    Data: tmp
##
## REML criterion at convergence: 339.5
##
## Scaled residuals:
##      Min       1Q   Median       3Q      Max
## -2.27983 -0.49617 -0.04304  0.60247  1.84696
##
## Random effects:
##  Groups   Name        Variance Std.Dev.
##  TreeID   (Intercept) 0.1925   0.4387
##  SiteID   (Intercept) 1.1570   1.0756
##  Residual             0.3783   0.6151
## Number of obs: 134, groups:  TreeID, 48; SiteID, 22
##
## Fixed effects:
##                                       Estimate Std. Error t value
## (Intercept)                           0.863045   0.377993   2.283
## TrapFan-trap Green                   -0.171676   0.408680  -0.420
## TrapFan-trap Yellow                   0.882763   0.408680   2.160
## Trap_DecoyNo decoy                    0.053465   0.210970   0.253
## TrapFan-trap Green:Trap_DecoyNo decoy   0.192915   0.273448   0.705
## TrapFan-trap Yellow:Trap_DecoyNo decoy -0.002426   0.273448  -0.009
```

Another possibility would be to use a Poisson Mixed model with the raw total numbers per trap with the log of the number of days as an offset. :

`glmer(Nb_Indiv ~ Trap * Trap_Decoy + offset(log(Nb_Days)) + (1|SiteID) + (1|TreeID), data = tmp, family = "poisson")`

The residual plots are a bit weirder but the overdispersion is reasonable (1.8). The conclusions are similar.

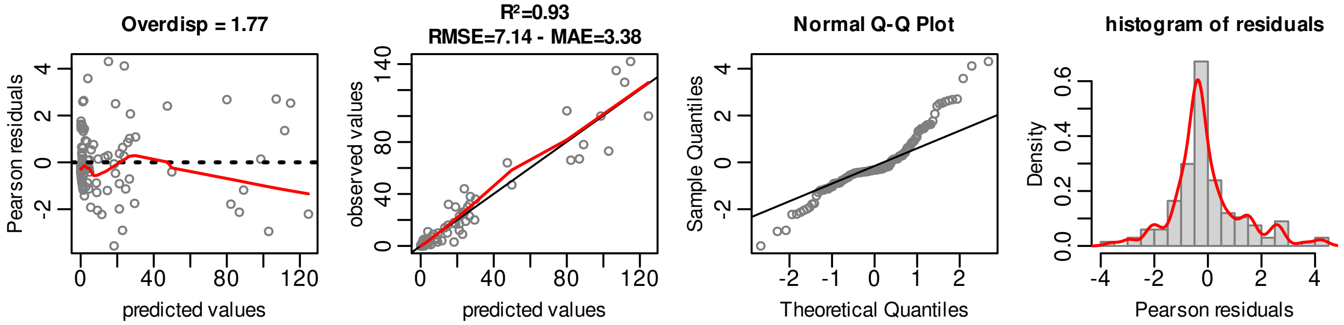

Figure 12:

Table 6: Analysis of Deviance Table (Type II Wald chisquare tests)

	Chisq	Df	Pr(>Chisq)
**Trap**	546.50	2	0.00
**Trap__Decoy**	0.79	1	0.37
**Trap:Trap__Decoy**	5.96	2	0.05

3.1.2 Are some species more attracted by decoys than others ?

The results of the previous section could be masked by specific responses to the decoys : some species might be attracted while others are not. We test this hypothesis here we a subset of species for which we have enough data (on Belgian and French Quercus sites only).

On the following graph the gray lines represent the total captures of a pair of similar traps from the same tree with or without decoy. The number of individual has been standardized to have a comparable number of trapping days (90 days). The red dots represent the average and the bars represent the 95% confidence interval (bootstrap).

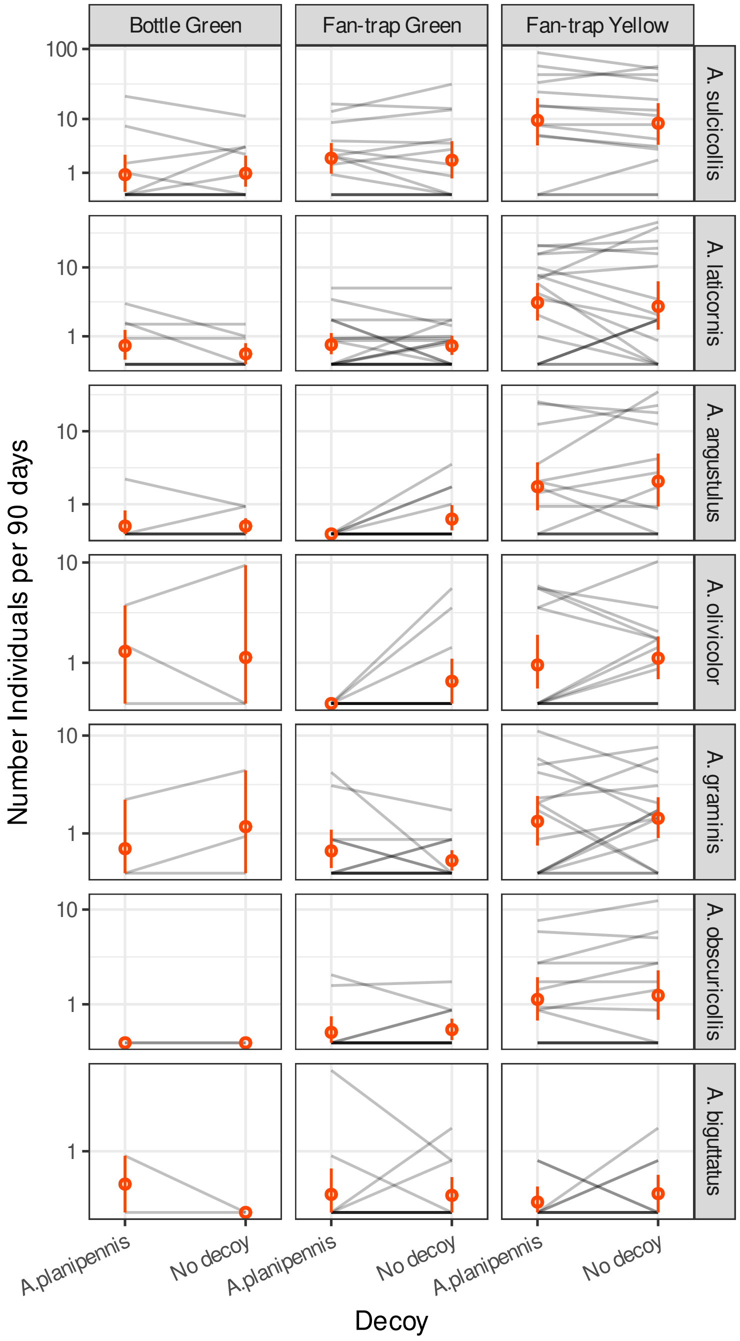

Figure 13:

We test these hypothesies with the following model : `lmer(log1p(Nb_Indiv_90_Days) ~ Trap * Trap_Decoy * Species + (1|SiteID) + (1|TreeID), data = tmp)`

NB : the residual plots show that the distribution is not as good as when we pool all species. However a Poisson mixed model of that complexity seems to systematically crash and on the other end, the amount of data is reasonable (central limit theorem) and the results are very clear (p-values either very high or very low).

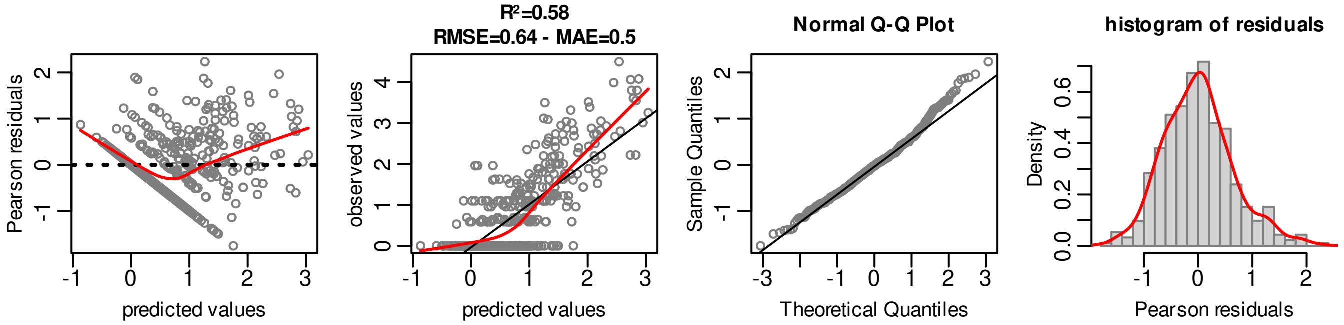

Figure 14:

Interpretation The Anova table shows no interaction trap x decoy x species nor decoy x species –> we have no evidence that the effect of the decoy could be different for some of the species.

We have highly significant Trap and Species main effect –> the number of captures are different between trap types (whatever the presence of decoys) and also different between species (some species are more often captured than others whatever the trap type).

We also have a highly significant Trap x Species interaction : this means that the differences between trap types are not of the same magnitude for the different species. From what we see on the graph : the differences between trap types for rare species like A. biguttatus seem to be very limited (they are close to 0 for all trap types) while there are clearly more catches in yellow fan traps for more common species.

Table 7: Analysis of Deviance Table (Type II Wald F tests with Kenward-Roger df)

	F	Df	Df.res	Pr(>F)
**Trap**	52.31	2	69.61	0.000000
**Trap__Decoy**	0.28	1	383.13	0.597745
**Species**	30.49	6	398.53	0.000000
**Trap:Trap__Decoy**	0.10	2	383.13	0.908431
**Trap:Species**	3.21	12	401.27	0.000209
**Trap__Decoy:Species**	0.45	6	383.13	0.845888
**Trap:Trap__Decoy:Species**	0.23	12	383.13	0.996717

3.1.3 Are males or females differently attracted by decoys ?

NB : the sex has been identified only on the catches from CRAW (Belgium) and INRAE (France). These sites are the ones with the most numerous captures so this is OK but it means also that we remove a lot of 0 values when we work on this subset of data. This could explain some differences in the averages computed here and in the other sections.

3.1.3.1 Species by species We can test if the sex has an influence on the captures for our 7 target species. We get a rather complex model with 4 fixed effects (and all interactions because we are precisely interested by these interactions) and 2 random effects (Site and Tree within the site) : `lmer(log1p(Nb) ~ Trap * Trap_Decoy * Species * Sex + (1|SiteID) + (1|TreeID), data = tmp_sex)`

Again the residual plots show that the distribution of the residuals deviates slightly from the needed Gaussian but we can make the same remark as in the previous section (too complex for a Poisson model, central limit theorem + results very clear).

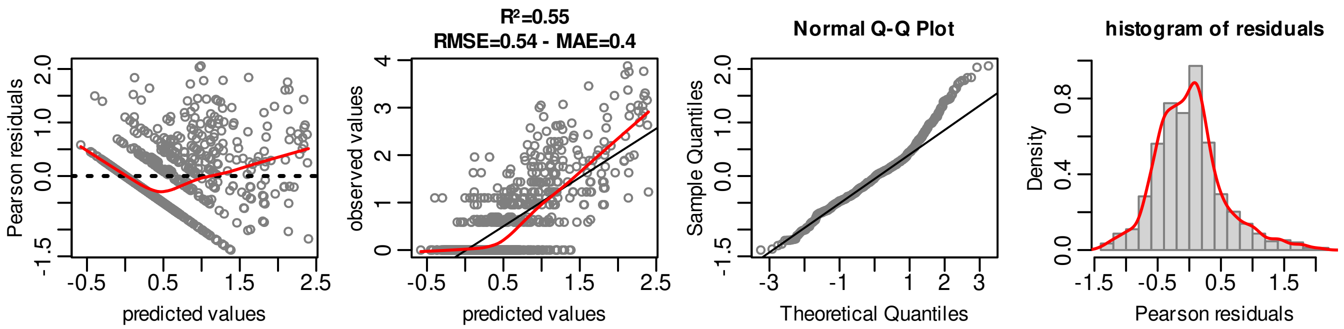

Figure 15:

None of the effects including the sex or the decoys are significant. So we cannot find any evidence in favor of a difference of attractivity to decoys between the sexes.

Table 8: Analysis of Deviance Table (Type II Wald F tests with Kenward-Roger df)

	F	Df	Df.res	Pr(>F)
**Trap**	91.69	2	67.92	0.000000
**Trap__Decoy**	0.10	1	749.01	0.755756
**Species**	55.89	6	763.52	0.000000
**Sex**	0.34	1	749.01	0.562239
**Trap:Trap__Decoy**	0.13	2	749.01	0.880534
**Trap:Species**	7.55	12	768.65	0.000000
**Trap__Decoy:Species**	0.69	6	749.01	0.656957
**Trap:Sex**	0.42	2	749.01	0.654183
**Trap__Decoy:Sex**	0.01	1	749.01	0.932030
**Species:Sex**	1.88	6	749.01	0.081325
**Trap:Trap__Decoy:Species**	0.20	12	749.01	0.998453
**Trap:Trap__Decoy:Sex**	0.50	2	749.01	0.607947
**Trap:Species:Sex**	1.01	12	749.01	0.441526
**Trap__Decoy:Species:Sex**	0.33	6	749.01	0.923664
**Trap:Trap__Decoy:Species:Sex**	0.26	12	749.01	0.994181

We can make a graphical representation of the data. Again no obiousvisual differnces between the sexes or between traps with or without decoys.

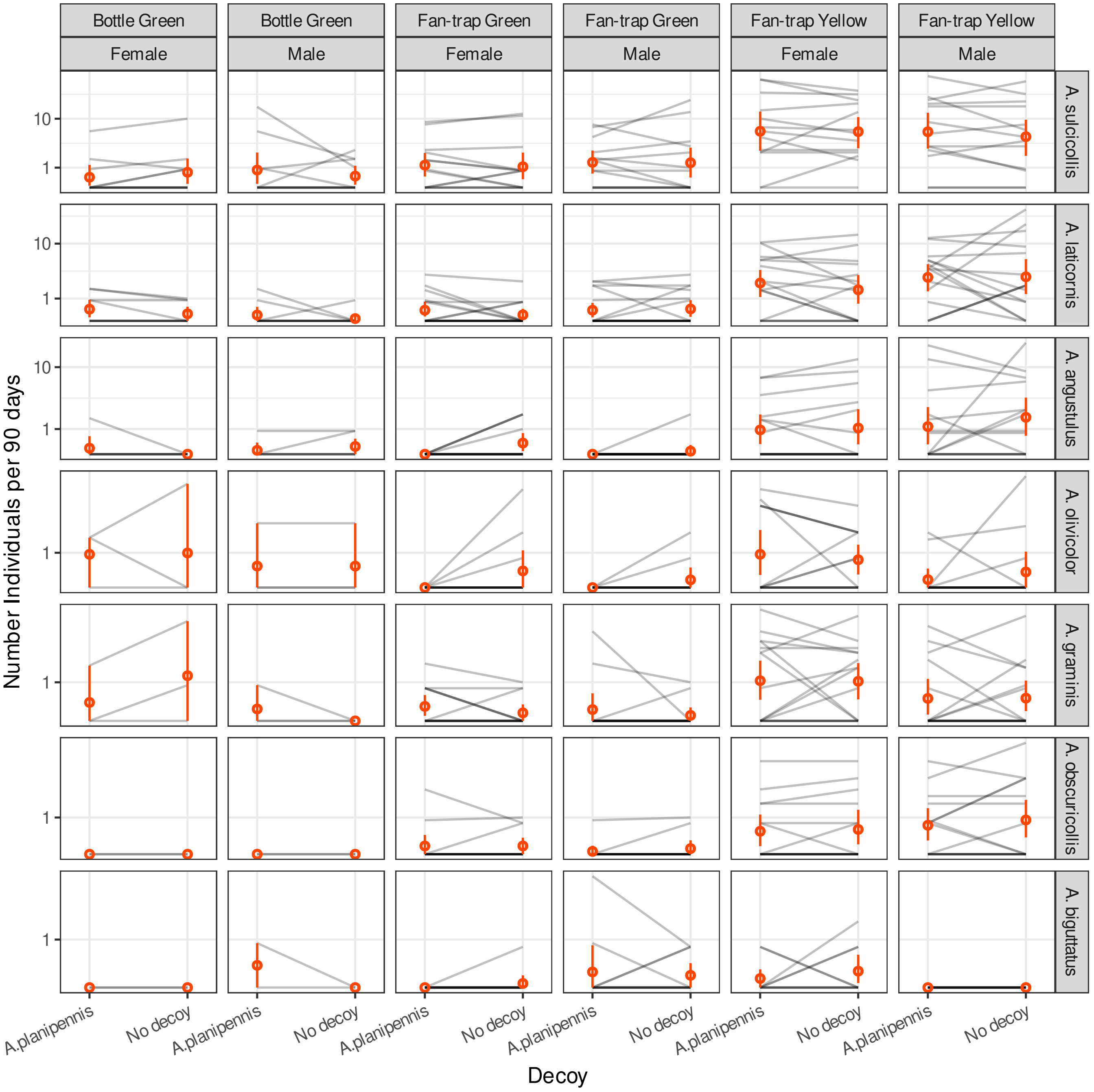

Figure 16:

3.1.3.2 All species grouped Here we use the data for all species grouped by sex (only for the CRAW and INRAE datasets and for pairs of bottle of Fan-Traps with or without decoy).

On the following graph the gray lines represent the total captures of a pair of similar traps from the same tree with or without decoy. The number of individual has been standardized to have a comparable number of trapping days (90 days). The red dots represent the average and the bars represent the 95% confidence interval (bootstrap).

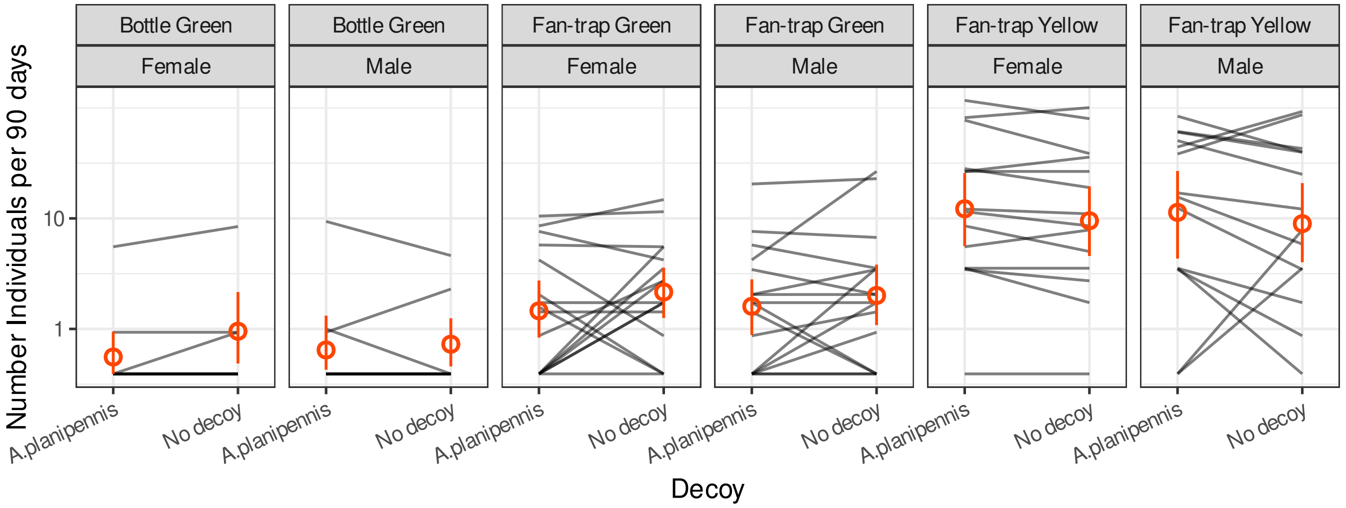

Figure 17:

We use the following gaussian mixed model : `m <- lmer(log1p(Nb) ~ Trap * Trap_Decoy * Sex + (1|SiteID) + (1|TreeID), data = tmp_sex)`

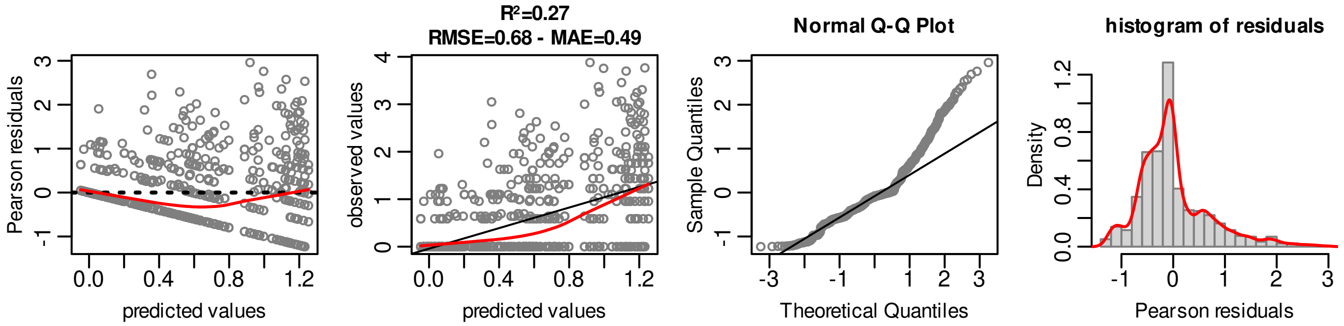

Figure 18:

Again, none of the effects including the sex or the decoys are significant. So we cannot find any evidence in favor of a difference of attractivity to decoys between the sexes.

Table 9: Analysis of Deviance Table (Type II Wald F tests with Kenward-Roger df)

	F	Df	Df.res	Pr(>F)
**Trap**	58.22	2	67.69	0.000000
**Trap_Decoy**	0.07	1	822.15	0.798261
**Sex**	0.23	1	822.15	0.633925
**Trap:Trap_Decoy**	0.14	2	822.15	0.873622
**Trap:Sex**	0.16	2	822.15	0.848920
**Trap_Decoy:Sex**	0.00	1	822.15	0.944128
**Trap:Trap_Decoy:Sex**	0.33	2	822.15	0.715444

3.1.4 Conclusions

We could not find any evidence of the effect of decoys on the number of captures. Therefore, we consider that we can safely group the catches of the pairs of traps with and without decoys for the rest of the analysis.

So,from now on the traps labelled as fan-traps or bottle traps are the pooled data of the pairs of traps with and without decoys.

3.2 Effect of trap type on the number of catches

3.2.1 What is the effect of trap type on the total number of individuals caught ?

We use a mixed model of the form = `lmer(log1p(Nb_Indiv_90_Days) ~ Trap_Type + Tree_Species + Year + (1|SiteID) + (1|TreeID), data = tmp)`

The response is the total number of individuals per trap *90 / Number of days of trapping (log(x+1) transformed). We use The Trap Type, Tree species (Fagus or Quercus) and Year as fixed effects and the SiteID and TreeID (nested in SiteID) as random effects.

NB : an equivalent Poisson model with number of trapping days as offset fails systematically…

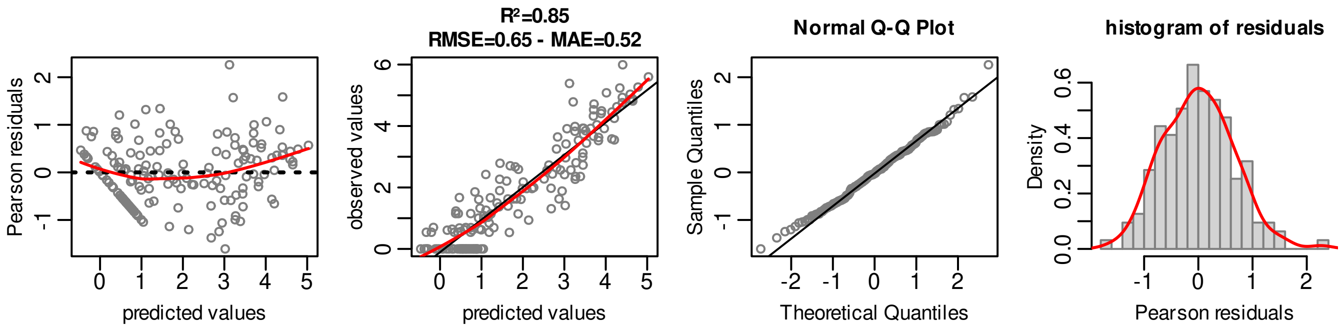

Figure 19:

The Anova table shows a highly significant Trap type effect, and also a more marginal effect of the tree species (Fagus vs Quercus) but no year effect

Table 10: Analysis of Deviance Table (Type II Wald F tests with Kenward-Roger df)

	F	Df	Df.res	Pr(>F)
**Trap_Type**	12.01	4	110.29	0.000000
**Tree_Species**	5.19	1	23.12	0.032223
**Year**	0.82	1	54.99	0.369305

In this model, the variance components of sites and tree random effects are rather high –> there is a lot of variation between sites but also between the trees on which the traps are placed

Variance_Component	Variance	Pct_Variance
TreeID	0.180	8.70
SiteID	1.316	63.77
Residual	0.568	27.53

The following graph provides a graphical summary of the model.

The gray lines represent traps from the same tree. In red : the average and 95% bootstrap Confidence Interval. The compact letter display shows which pairwise comparisons are significantly different from each other (with p-value correction). The fan-traps or bottle traps with and without decoys have been pooled together.

Note that there is no significant difference between the bottle traps and the fan-traps but this could be due to the fact that these two type of traps where not present the same year so it is difficult to compare them with confidence

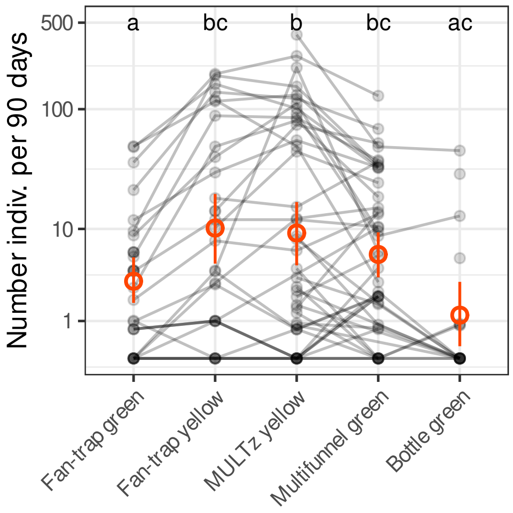

Figure 20:

Here is the detail of the pairwise comparisons :

```
##
##   Simultaneous Tests for General Linear Hypotheses
##
## Multiple Comparisons of Means: Tukey Contrasts
##
##
## Fit: lmer(formula = log1p(Nb_Indiv_90_Days) ~ Trap_Type + Tree_Species +
##     Year + (1 | SiteID) + (1 | TreeID), data = tmp)
##
## Linear Hypotheses:
##                                         Estimate Std. Error z value Pr(>|z|)
## Fan-trap yellow - Fan-trap green == 0      0.9526     0.2027   4.700  < 0.001 ***
## MULTz yellow - Fan-trap green == 0         1.2042     0.1977   6.090  < 0.001 ***
## Multifunnel green - Fan-trap green == 0    0.7593     0.1961   3.871  < 0.001 ***
## Bottle green - Fan-trap green == 0         0.3095     0.3028   1.022  0.83789
## MULTz yellow - Fan-trap yellow == 0        0.2517     0.1936   1.300  0.67984
## Multifunnel green - Fan-trap yellow == 0  -0.1933     0.1921  -1.007  0.84542
## Bottle green - Fan-trap yellow == 0       -0.6430     0.3002  -2.142  0.19316
## Multifunnel green - MULTz yellow == 0     -0.4450     0.1668  -2.667  0.05566 .
## Bottle green - MULTz yellow == 0          -0.8947     0.2558  -3.498  0.00404 **
## Bottle green - Multifunnel green == 0     -0.4497     0.2611  -1.722  0.40681
## ---
## Signif. codes:  0 '***' 0.001 '**' 0.01 '*' 0.05 '.' 0.1 ' ' 1
## (Adjusted p values reported -- single-step method)
```

Note also that there are plenty of sites where we have very few individuals and this probably tend to decrease artificially the differences between traps because if there are really very few individuals present the traos will be very limited in the total captures possible. (NB : remember that we have already removed all trees in which no individuals of each species were captured).

This is for example what we obtain if we keep only the trees with more than 5 individuals captured (a rather arbitrary choice). The results are similar if we chose other arbitrary thresholds like 3, 5, 7 or 10.

Now the Hungarian MULTz traps have significantly more captures than the multifunnel green while the yellow fan-traps are intermediate (no significant difference between fan-traps yellow and multifunnel green or

MULTz). The green fan-traps have significantly less captures than these 3 type of traps. The green bottle traps captures are only significantly lower than the MULTz. However we had very few traps with captures in bottle traps and the fan traps have been placed on the field during a different year which makes the comparison not totally fair.

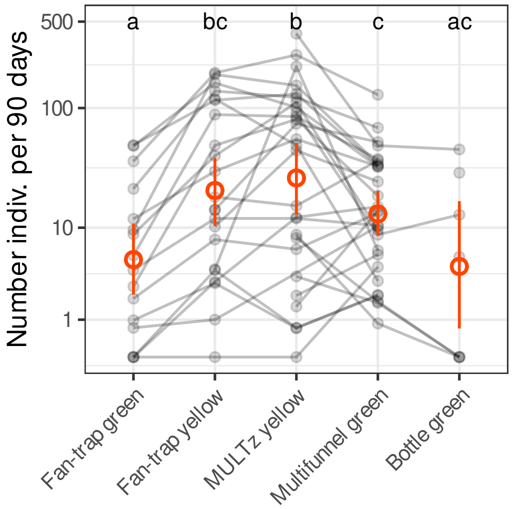

Figure 21:

Detail of the pairwise comparisons with p-value correction :

```
##
##   Simultaneous Tests for General Linear Hypotheses
##
## Multiple Comparisons of Means: Tukey Contrasts
##
##
## Fit: lmer(formula = log1p(Nb_Indiv_90_Days) ~ Trap_Type + Tree_Species +
##     Year + (1 | SiteID) + (1 | TreeID), data = tmp)
##
## Linear Hypotheses:
##                                           Estimate Std. Error z value Pr(>|z|)
## Fan-trap yellow - Fan-trap green == 0      1.2662     0.2684   4.718  < 0.001 ***
## MULTz yellow - Fan-trap green == 0         1.6356     0.2651   6.169  < 0.001 ***
## Multifunnel green - Fan-trap green == 0    1.0165     0.2618   3.882  < 0.001 ***
## Bottle green - Fan-trap green == 0         0.1974     0.4788   0.412  0.99341
## MULTz yellow - Fan-trap yellow == 0        0.3694     0.2391   1.545  0.51462
## Multifunnel green - Fan-trap yellow == 0  -0.2498     0.2361  -1.058  0.81715
## Bottle green - Fan-trap yellow == 0       -1.0688     0.4650  -2.299  0.13488
## Multifunnel green - MULTz yellow == 0     -0.6191     0.2153  -2.876  0.02979 *
## Bottle green - MULTz yellow == 0          -1.4382     0.4277  -3.363  0.00631 **
## Bottle green - Multifunnel green == 0     -0.8191     0.4277  -1.915  0.29238
## ---
## Signif. codes:  0 '***' 0.001 '**' 0.01 '*' 0.05 '.' 0.1 ' ' 1
## (Adjusted p values reported -- single-step method)
```

3.2.2 What is the relative importance of trap type, site and tree to explain the number of catches ?

We use a model with only random effects for an easy decomposition of the variance. We use the log of the number of individuals per 90 days as response and the trap type, site and tree as random effects.

The variation between sites is by far more imporrtant than the variation between trees or between trap types

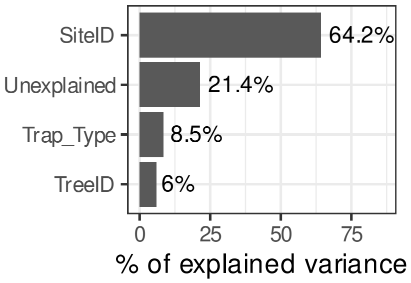

Figure 22:

```
## Linear mixed model fit by REML ['lmerMod']
## Formula: log1p(Nb_Indiv_90_Days) ~ (1 | Trap_Type) + (1 | SiteID) + (1 |      TreeID)
##    Data: tmp
##
## REML criterion at convergence: 458.9
##
## Scaled residuals:
##      Min       1Q   Median       3Q      Max
## -2.11972 -0.64660 -0.04528  0.56909  3.06772
##
## Random effects:
##  Groups    Name        Variance Std.Dev.
##  TreeID    (Intercept) 0.1579   0.3974
##  SiteID    (Intercept) 1.6983   1.3032
##  Trap_Type (Intercept) 0.2251   0.4744
##  Residual              0.5658   0.7522
## Number of obs: 158, groups:  TreeID, 54; SiteID, 26; Trap_Type, 5
##
## Fixed effects:
##             Estimate Std. Error t value
## (Intercept)   1.7467     0.3444   5.071
```

3.2.3 What is the impact of the area of the trap ?

The traps used have rather different catching areas and we can suppose that this can have an impact on the number of catches. However we have only limited ways to test explicitly this hypothesis with our dataset. To do that we would need similar trap types with varying areas in our experimental setup. We can standardize the catches by the area of the trap but by doing that we would "force" or "impose" on our data a linear effect of the area without really knowing its real effect. So this section is just showing what happens to our results when we correct of the trap size but this should not be used as is.

Dimensions of the traps :

- Bottle traps ~ trapeze with 15cm base x 8cm base x 15 cm height x 2 sides x 2 pairs of traps on top of each other = 690 cm^2
- Fan-traps = 15x20 cm rectangle x 2 sides x 2 pairs of traps hanging on top of each other = 1200 cm^2
- MULTz traps: 23x15 cm x 2 sides x 4 pieces = 2760 cm^2
- Multifunnel green traps: they are a cylinder but we will consider a projection as if they were "flat" –> 19 cm diameter x 88cm height (11 pieces x 8cm) and x 2 sides = 3344 cm^2

NB : Imrei et al 2020 DOI: 10.1093/forestry/cpz071. used an area of 11 160 cm2 for 12 funnel of Lindgren funnel traps

We use a statistical analysis approach identical to the previous section. We just divided the catches by the area of the trap and then multiplied that value by 2000 cm^2 to have a common trapping area reference

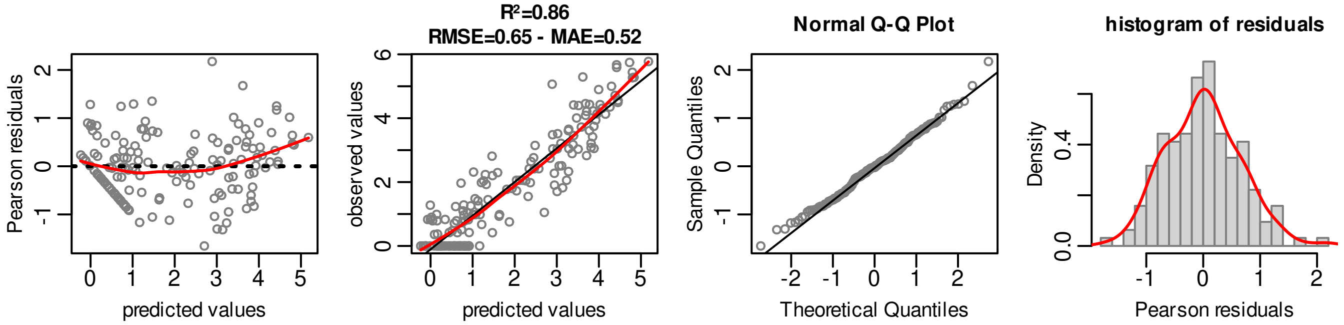

Figure 23:

The Anova table shows a highly significant Trap type effect and of the tree species (Fagus vs Quercus) but no year effect

Table 12: Analysis of Deviance Table (Type II Wald F tests with Kenward-Roger df)

	F	Df	Df.res	Pr(>F)
**Trap_Type**	9.51	4	110.18	0.000001
**Tree_Species**	5.36	1	23.15	0.029868
**Year**	0.19	1	54.63	0.666615

The following graph provides a graphical summary of the model.

Now the yellow traps are capturing significantly more than the other types. The yellow fan traps capture the most but these catches are not significantly different from the MULTz.

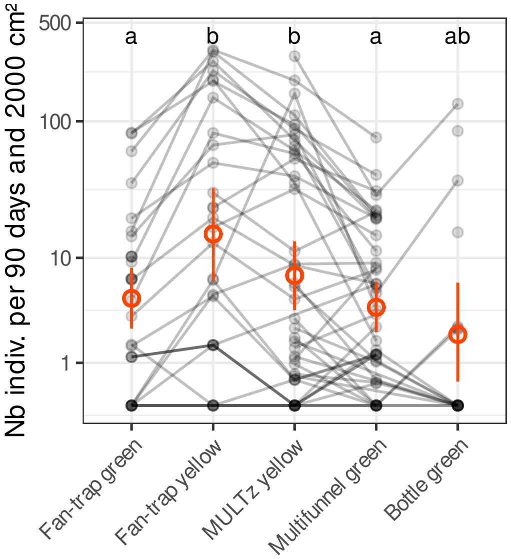

Figure 24:

Here is the detail of the pairwise comparisons :

```
##
##   Simultaneous Tests for General Linear Hypotheses
##
## Multiple Comparisons of Means: Tukey Contrasts
##
##
## Fit: lmer(formula = log1p(Nb_Indiv_90_Days) ~ Trap_Type + Tree_Species +
##     Year + (1 | SiteID) + (1 | TreeID), data = tmp)
##
## Linear Hypotheses:
##                                          Estimate Std. Error z value Pr(>|z|)
## Fan-trap yellow - Fan-trap green == 0      1.0213     0.2038   5.012  < 0.001 ***
## MULTz yellow - Fan-trap green == 0         0.6778     0.1988   3.409  0.00559 **
## Multifunnel green - Fan-trap green == 0    0.1064     0.1972   0.539  0.98219
## Bottle green - Fan-trap green == 0         0.2512     0.3045   0.825  0.91876
## MULTz yellow - Fan-trap yellow == 0       -0.3435     0.1946  -1.765  0.38129
## Multifunnel green - Fan-trap yellow == 0  -0.9149     0.1930  -4.740  < 0.001 ***
## Bottle green - Fan-trap yellow == 0       -0.7701     0.3017  -2.552  0.07512 .
## Multifunnel green - MULTz yellow == 0     -0.5715     0.1677  -3.408  0.00557 **
## Bottle green - MULTz yellow == 0          -0.4266     0.2571  -1.659  0.44594
## Bottle green - Multifunnel green == 0      0.1449     0.2625   0.552  0.98060
## ---
## Signif. codes:  0 '***' 0.001 '**' 0.01 '*' 0.05 '.' 0.1 ' ' 1
## (Adjusted p values reported -- single-step method)
```

3.2.4 Are some species more attracted by certain types of traps ?

We use a mixed model of the form = `lmer(log1p(Nb_Indiv_90_Days) ~ Trap_Type*Species + (1|SiteID) + (1|TreeID), data = tmp)`

The response is the total number of individuals of each species per trap *90 / Number of days of trapping (log(x+1) transformed). We use The Trap Type, Agrilus species and their interaction as fixed effects and the SiteID and TreeID as random effects. The tree species was not included here because we kept only the Quercus sites from the dataset (beacuse the target species are living mostly on Quercus). The year was excluded to simplify the model

NB : an equivalent Poisson model with number of trapping days as offset fails systematically…

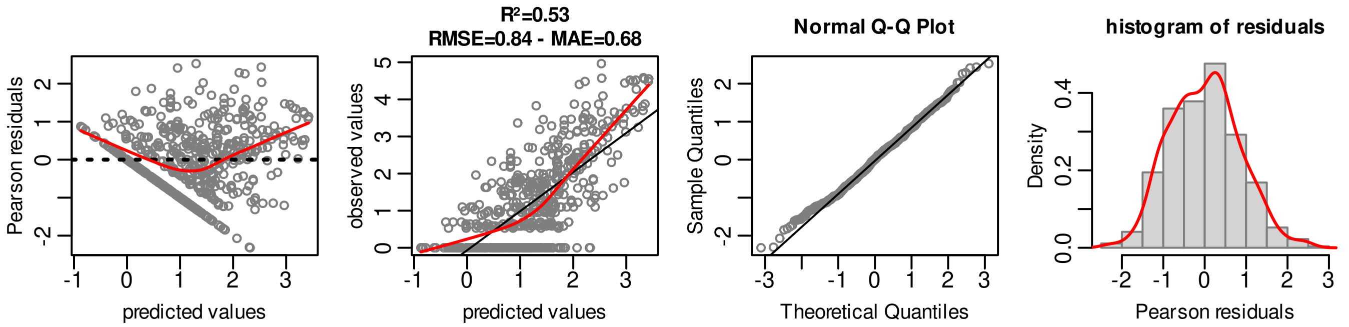

Figure 25:

The Anova table shows a highly significant Trap type x species effect. This means that there are differences in the amount of captures between trap types but that these differences are not the same depending on the Agrilus species.

Table 13: Analysis of Deviance Table (Type II Wald F tests with Kenward-Roger df)

	F	Df	Df.res	Pr(>F)
**Trap_Type**	32.03	4	464.00	0.000000
**Species**	23.16	6	479.93	0.000000
**Trap_Type:Species**	1.69	24	468.24	0.021929

The following graph provides a graphical summary of the model.

On the following graph the gray lines represent the total captures for each species in a given trap from the same tree. So the lines are relating directly comparable captures from different types of traps. The number of individuals has been standardized to have a comparable number of trapping days (90 days). The red dots represent the average and the bars represent the 95% confidence interval (bootstrap).

Trap types sharing the same letter are not significantly different. The lettres should only be compared within a species (you cannot compare the trap types between species using these lettres). To obtain these letters we recomputed a separate mixed model for each species then performed all pairwise comparisons with p-value corrections (for multiple testing). We used this approach because it is much more easy to obtain the letters display in an automated manner with this approach. Some people might however criticize this approach (see below).

Interpretation : The differences between trap types are more or less similar for the 6 most common species but A. biguttatus show a slightly different pattern. For the 6 most common species, the captures tend to

be more abundant in the 2 yellow traps (Fan-Traps and MUTLTz) and lower in the green fan-traps and to a lower extend in the multifiunnel green ones. The captures in the green bottle traps are often very low but most of the time the differences with the other trap types is not significant (probably due to several reasons : the number of replicates is low, they were never places in the same sites as the Fan-Traps,. . .). The differences between traps tend to be less obvious as the global abundance of the species tend to decrease.

In A. biguttatus however the highest captures occured in the multifunnel green trap and the green and yellow Fan-traps captured significantly less individuals, while the difference with the yellow MULTz is not significant.

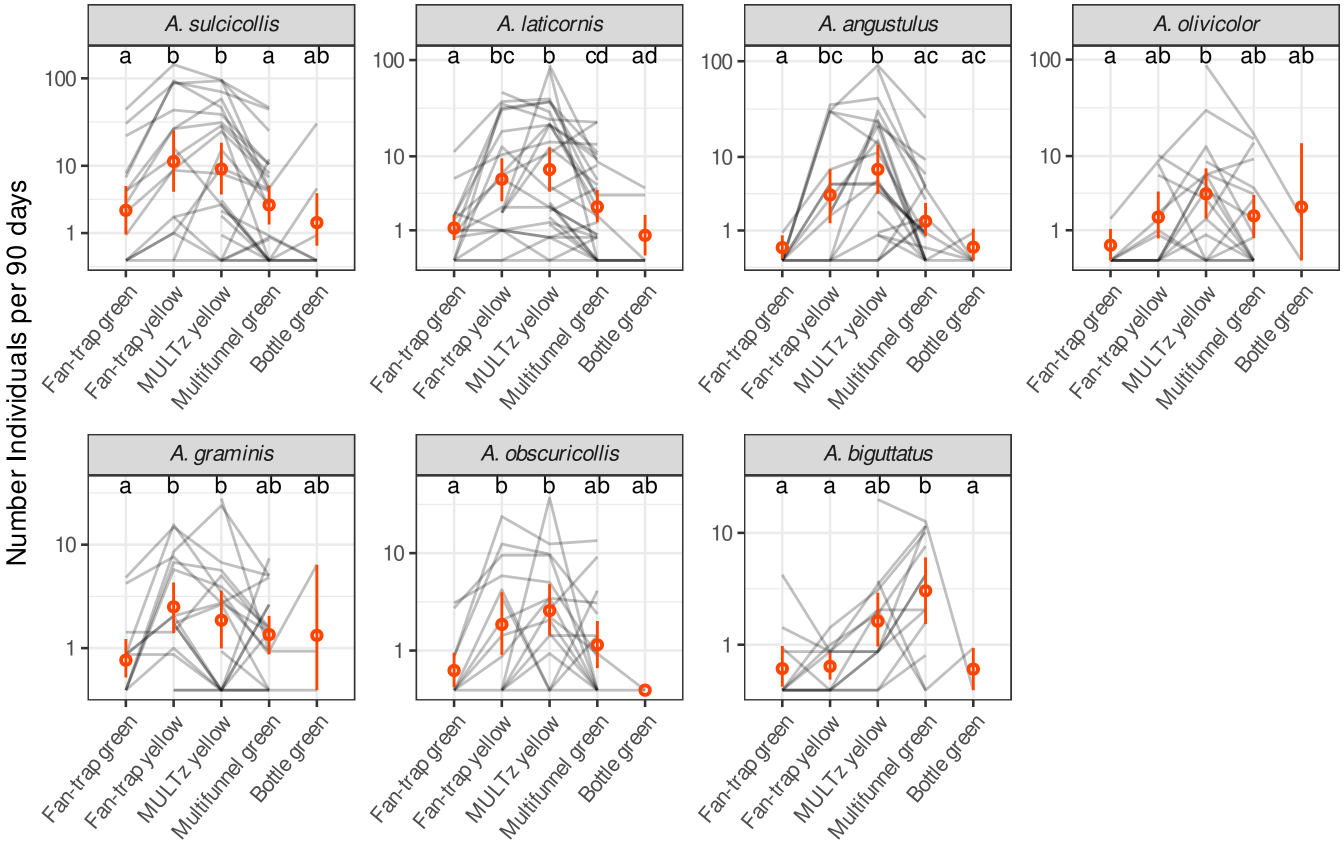

Figure 26:

When we compute the pairwise comparisons with a separate model for each species, the p-value correction is done only for these 10 pairwise comparison within each species while in reality these 10 comparisons are repeated for the 7 species.

It is possible to write manually a contrast matrix comparing the traps within each species and based on the full model with `Trap_type * Species` as fixed effects. In that case the p-value correction is done on all 70 pairwise comparisons together and some people might consider this as “more correct”. The higher the number of comparison the stronger the p-value correction and the lower the statistical power.

One should keep in mind that the choice of the p-value correction method (here a “Tukey like” single-step method) is rather arbitrary and choosing other methods will provide other results. The choice is always a matter of which kind of error you favor : do you prefer more false negative (type II error) or more false positives (type I error) ? For example, with a Bonferoni correction, the correction will be even stronger and you will have more false negatives. So, in the end, the previous approach with a separate model for each

species might be considered as a reasonable compromise in my opinion and I think that few people with bother to tediously build manually a 35 x 70 contrast matrix (as I did below) with a relatively high risk of errors…

The following table shows the results of all 70 pairwise comparisons computed with a manually build contrast matrix. It could be possible to derive a compact letter display from this table but this is rather tedious and must be done entirely manually.

You can see that with this approach we "lost" all significant differences for the 4 most rare species : A.olivicolor, graminis, obscuricollis, biguttatus.

```
##
##   Simultaneous Tests for General Linear Hypotheses
##
## Fit: lmer(formula = log1p(Nb_Indiv_90_Days) ~ -1 + Trap_Type:Species +
##     (1 | SiteID) + (1 | TreeID), data = tmp)
##
## Linear Hypotheses:
##                                                             Estimate Std. Error z value Pr(>|z|)
## A.sulcicollis : Fan-trap green - Fan-trap yellow == 0      -1.227997   0.307256  -3.997    <0.01 **
## A.sulcicollis : Fan-trap green - MULTz yellow == 0         -1.235100   0.284896  -4.335    <0.01 ***
## A.sulcicollis : Fan-trap green - Multifunnel green == 0    -0.290217   0.286630  -1.013   1.0000
## A.sulcicollis : Fan-trap green - Bottle green == 0         -0.232114   0.369969  -0.627   1.0000
## A.sulcicollis : Fan-trap yellow - MULTz yellow == 0        -0.007103   0.283543  -0.025   1.0000
## A.sulcicollis : Fan-trap yellow - Multifunnel green == 0    0.937779   0.285267   3.287   0.0567 .
## A.sulcicollis : Fan-trap yellow - Bottle green == 0         0.995883   0.369198   2.697   0.3075
## A.sulcicollis : MULTz yellow - Multifunnel green == 0       0.944883   0.255374   3.700   0.0131 *
## A.sulcicollis : MULTz yellow - Bottle green == 0            1.002986   0.335586   2.989   0.1435
## A.sulcicollis : Multifunnel green - Bottle green == 0       0.058103   0.339309   0.171   1.0000
## A.laticornis : Fan-trap green - Fan-trap yellow == 0       -1.159831   0.274511  -4.225    <0.01 **
## A.laticornis : Fan-trap green - MULTz yellow == 0          -1.501973   0.256962  -5.845    <0.01 ***
## A.laticornis : Fan-trap green - Multifunnel green == 0     -0.644206   0.256962  -2.507   0.4586
## A.laticornis : Fan-trap green - Bottle green == 0          -0.203260   0.376509  -0.540   1.0000
## A.laticornis : Fan-trap yellow - MULTz yellow == 0         -0.342142   0.257891  -1.327   0.9995
## A.laticornis : Fan-trap yellow - Multifunnel green == 0     0.515625   0.257891   1.999   0.8732
## A.laticornis : Fan-trap yellow - Bottle green == 0          0.956572   0.377495   2.534   0.4360
## A.laticornis : MULTz yellow - Multifunnel green == 0        0.857767   0.234174   3.663   0.0152 *
## A.laticornis : MULTz yellow - Bottle green == 0             1.298714   0.354271   3.666   0.0148 *
## A.laticornis : Multifunnel green - Bottle green == 0        0.440947   0.354271   1.245   0.9998
## A.angustulus : Fan-trap green - Fan-trap yellow == 0       -1.231383   0.322761  -3.815    <0.01 **
## A.angustulus : Fan-trap green - MULTz yellow == 0          -2.004215   0.301027  -6.658    <0.01 ***
## A.angustulus : Fan-trap green - Multifunnel green == 0     -0.761689   0.303014  -2.514   0.4544
## A.angustulus : Fan-trap green - Bottle green == 0          -0.443340   0.430128  -1.031   1.0000
## A.angustulus : Fan-trap yellow - MULTz yellow == 0         -0.772832   0.293207  -2.636   0.3533
## A.angustulus : Fan-trap yellow - Multifunnel green == 0     0.469694   0.295213   1.591   0.9911
## A.angustulus : Fan-trap yellow - Bottle green == 0          0.788043   0.425090   1.854   0.9397
## A.angustulus : MULTz yellow - Multifunnel green == 0        1.242526   0.266546   4.662    <0.01 ***
## A.angustulus : MULTz yellow - Bottle green == 0             1.560875   0.397063   3.931    <0.01 **
## A.angustulus : Multifunnel green - Bottle green == 0        0.318349   0.401618   0.793   1.0000
## A.olivicolor : Fan-trap green - Fan-trap yellow == 0       -0.607829   0.342260  -1.776   0.9628
## A.olivicolor : Fan-trap green - MULTz yellow == 0          -1.110158   0.332972  -3.334   0.0488 *
## A.olivicolor : Fan-trap green - Multifunnel green == 0     -0.604265   0.332972  -1.815   0.9523
## A.olivicolor : Fan-trap green - Bottle green == 0          -0.383248   0.590154  -0.649   1.0000
## A.olivicolor : Fan-trap yellow - MULTz yellow == 0         -0.502330   0.319345  -1.573   0.9924
## A.olivicolor : Fan-trap yellow - Multifunnel green == 0     0.003563   0.319345   0.011   1.0000
## A.olivicolor : Fan-trap yellow - Bottle green == 0          0.224581   0.582988   0.385   1.0000
## A.olivicolor : MULTz yellow - Multifunnel green == 0        0.505893   0.305853   1.654   0.9848
## A.olivicolor : MULTz yellow - Bottle green == 0             0.726910   0.573958   1.266   0.9998
## A.olivicolor : Multifunnel green - Bottle green == 0        0.221017   0.573958   0.385   1.0000
## A.graminis : Fan-trap green - Fan-trap yellow == 0         -0.891825   0.328448  -2.715   0.2944
## A.graminis : Fan-trap green - MULTz yellow == 0            -0.693701   0.317001  -2.188   0.7408
## A.graminis : Fan-trap green - Multifunnel green == 0       -0.457971   0.317001  -1.445   0.9979
## A.graminis : Fan-trap green - Bottle green == 0            -0.561225   0.586827  -0.956   1.0000
## A.graminis : Fan-trap yellow - MULTz yellow == 0            0.198124   0.303456   0.653   1.0000
## A.graminis : Fan-trap yellow - Multifunnel green == 0       0.433855   0.303456   1.430   0.9983
## A.graminis : Fan-trap yellow - Bottle green == 0            0.330600   0.579997   0.570   1.0000
## A.graminis : MULTz yellow - Multifunnel green == 0          0.235730   0.289308   0.815   1.0000
## A.graminis : MULTz yellow - Bottle green == 0               0.132476   0.567672   0.233   1.0000
## A.graminis : Multifunnel green - Bottle green == 0         -0.103254   0.567672  -0.182   1.0000
## A.obscuricollis : Fan-trap green - Fan-trap yellow == 0    -0.840495   0.353429  -2.378   0.5745
```

```
## A.obscuricollis : Fan-trap green - MULTz yellow == 0       -1.111631   0.343721  -3.234   0.0681 .
## A.obscuricollis : Fan-trap green - Multifunnel green == 0  -0.512756   0.343721  -1.492   0.9966
## A.obscuricollis : Fan-trap green - Bottle green == 0        0.070540   0.701114   0.101   1.0000
## A.obscuricollis : Fan-trap yellow - MULTz yellow == 0      -0.271136   0.326966  -0.829   1.0000
## A.obscuricollis : Fan-trap yellow - Multifunnel green == 0  0.327739   0.326966   1.002   1.0000
## A.obscuricollis : Fan-trap yellow - Bottle green == 0       0.911036   0.693471   1.314   0.9996
## A.obscuricollis : MULTz yellow - Multifunnel green == 0     0.598875   0.315266   1.900   0.9222
## A.obscuricollis : MULTz yellow - Bottle green == 0          1.182171   0.683801   1.729   0.9731
## A.obscuricollis : Multifunnel green - Bottle green == 0     0.583296   0.683801   0.853   1.0000
## A.biguttatus : Fan-trap green - Fan-trap yellow == 0       -0.009223   0.372826  -0.025   1.0000
## A.biguttatus : Fan-trap green - MULTz yellow == 0          -0.640032   0.358231  -1.787   0.9601
## A.biguttatus : Fan-trap green - Multifunnel green == 0     -1.103972   0.358231  -3.082   0.1090
## A.biguttatus : Fan-trap green - Bottle green == 0           0.405664   0.696794   0.582   1.0000
## A.biguttatus : Fan-trap yellow - MULTz yellow == 0         -0.630809   0.366009  -1.723   0.9741
## A.biguttatus : Fan-trap yellow - Multifunnel green == 0    -1.094749   0.366009  -2.991   0.1424
## A.biguttatus : Fan-trap yellow - Bottle green == 0          0.414888   0.701067   0.592   1.0000
## A.biguttatus : MULTz yellow - Multifunnel green == 0       -0.463940   0.349756  -1.326   0.9995
## A.biguttatus : MULTz yellow - Bottle green == 0             1.045696   0.688642   1.518   0.9955
## A.biguttatus : Multifunnel green - Bottle green == 0        1.509636   0.688642   2.192   0.7375
## ---
## Signif. codes:  0 '***' 0.001 '**' 0.01 '*' 0.05 '.' 0.1 ' ' 1
## (Adjusted p values reported -- single-step method)
```

3.2.5 Are males or females differently attracted by certain type of traps ?

3.2.5.1 Species by species We use the following model to test for the effect of sex : `lmer(log1p(Nb) ~ Trap_Type * Species * Sex + (1|SiteID) + (1|TreeID), data = tmp_sex)`

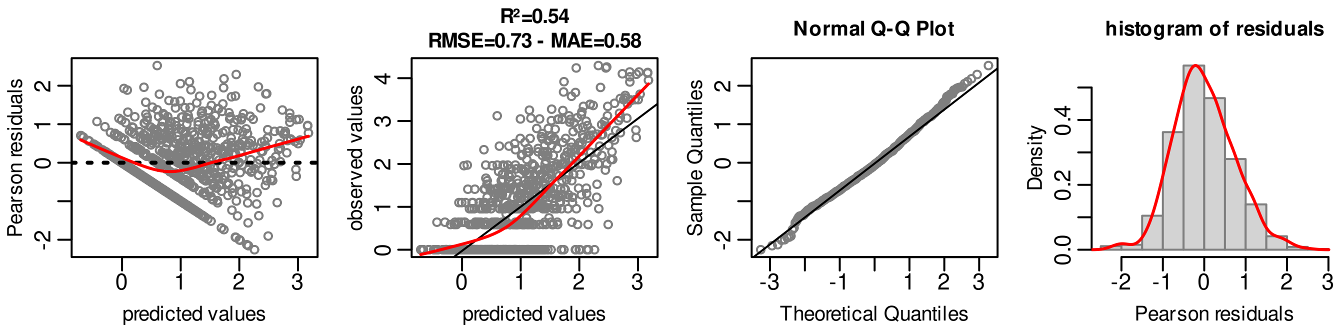

Figure 27:

All effects involving the sex are not significant except for `Species x Sex`. In theory this would mean that there are differences in captures between males and females depending on the Agrilus species (but independent of the Trap Type). However when comparing males vs females catches for all combinations of Species and trap type (with p-value correction for multiple testing, see below), none are significantly different.

So we found no evidence that one type of trap might attract more the males or the females.

Table 14: Analysis of Deviance Table (Type II Wald F tests with Kenward-Roger df)

	F	Df	Df.res	Pr(>F)
**Trap__Type**	56.43	4	776.80	0.000000
**Species**	50.28	6	830.21	0.000000
**Sex**	0.76	1	814.42	0.384891
**Trap__Type:Species**	4.05	24	824.47	0.000000
**Trap__Type:Sex**	1.30	4	814.42	0.266531
**Species:Sex**	3.06	6	814.42	0.005708
**Trap__Type:Species:Sex**	0.81	24	814.42	0.720595

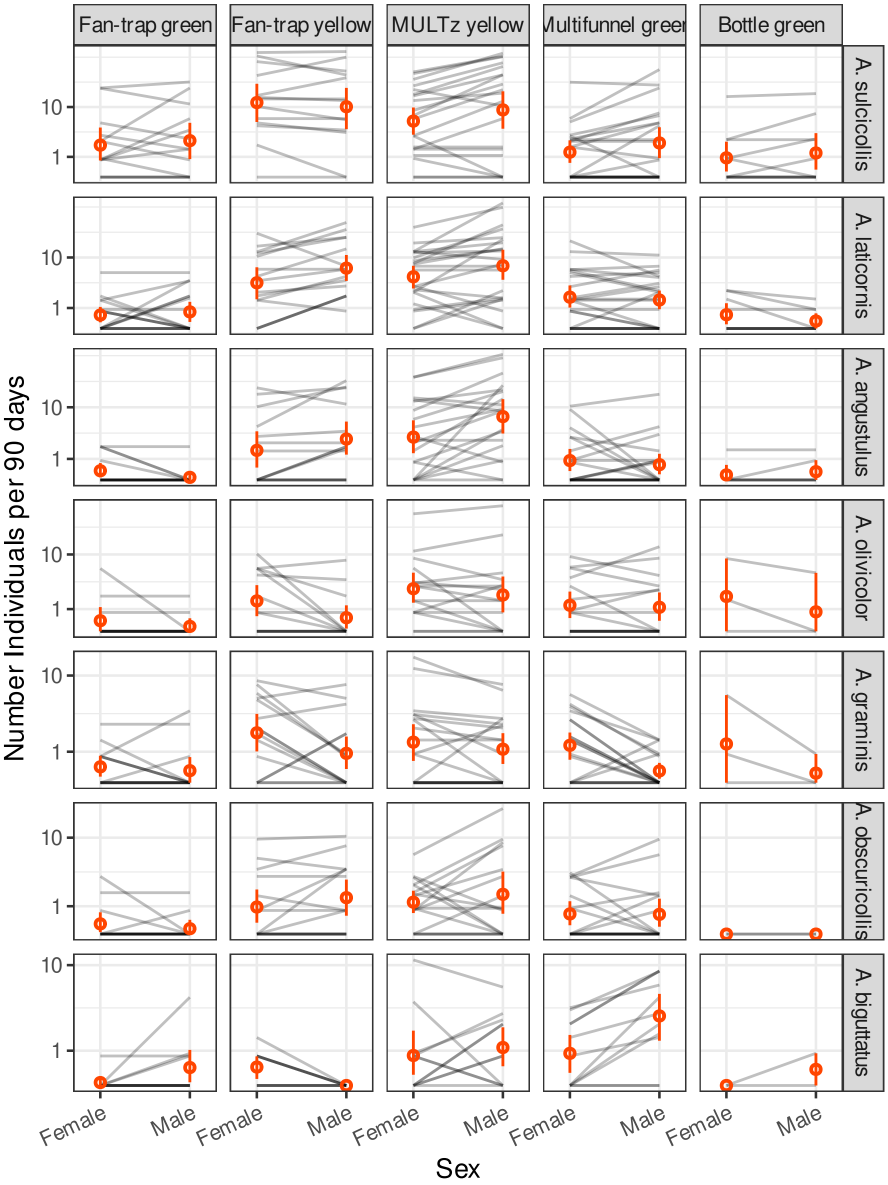
Fan-trap green
Fan-trap yellow
MULTz yellow
Multifunnel green
Bottle green
A. sulcicollis
A. laticornis
A. angustulus
A. olivicolor
A. graminis
A. obscuricollis
A. biguttatus
Number Individuals per 90 days
10
1
Female
Male
Sex

Figure 28:

Multiple comparisons :

```
##
##   Simultaneous Tests for General Linear Hypotheses
##
## Fit: lmer(formula = log1p(Nb) ~ -1 + Sex:Trap_Type:Species + (1 |
##     SiteID) + (1 | TreeID), data = tmp_sex)
##
## Linear Hypotheses:
##                                                        Estimate Std. Error z value Pr(>|z|)
## A.sulcicollis - Fan-trap green : Female - Male == 0      -0.153065   0.301084  -0.508    1.000
## A.sulcicollis - Fan-trap yellow : Female - Male == 0      0.142421   0.301084   0.473    1.000
## A.sulcicollis - MULTz yellow : Female - Male == 0        -0.382334   0.242742  -1.575    0.986
## A.sulcicollis - Multifunnel green : Female - Male == 0   -0.315277   0.242742  -1.299    0.999
## A.sulcicollis - Bottle green : Female - Male == 0        -0.166311   0.343289  -0.484    1.000
## A.laticornis - Fan-trap green : Female - Male == 0       -0.105783   0.271394  -0.390    1.000
## A.laticornis - Fan-trap yellow : Female - Male == 0      -0.494489   0.280294  -1.764    0.941
## A.laticornis - MULTz yellow : Female - Male == 0         -0.372152   0.231445  -1.608    0.982
## A.laticornis - Multifunnel green : Female - Male == 0     0.094953   0.231445   0.410    1.000
## A.laticornis - Bottle green : Female - Male == 0          0.213335   0.361858   0.590    1.000
## A.angustulus - Fan-trap green : Female - Male == 0        0.218391   0.301084   0.725    1.000
## A.angustulus - Fan-trap yellow : Female - Male == 0      -0.374768   0.290132  -1.292    1.000
## A.angustulus - MULTz yellow : Female - Male == 0         -0.676265   0.249048  -2.715    0.207
## A.angustulus - Multifunnel green : Female - Male == 0     0.135758   0.249048   0.545    1.000
## A.angustulus - Bottle green : Female - Male == 0         -0.106976   0.443184  -0.241    1.000
## A.olivicolor - Fan-trap green : Female - Male == 0        0.178190   0.327313   0.544    1.000
## A.olivicolor - Fan-trap yellow : Female - Male == 0       0.524092   0.301084   1.741    0.949
## A.olivicolor - MULTz yellow : Female - Male == 0          0.195646   0.280294   0.698    1.000
## A.olivicolor - Multifunnel green : Female - Male == 0     0.063142   0.280294   0.225    1.000
## A.olivicolor - Bottle green : Female - Male == 0          0.480276   0.626757   0.766    1.000
## A.graminis - Fan-trap green : Female - Male == 0          0.085341   0.301084   0.283    1.000
## A.graminis - Fan-trap yellow : Female - Male == 0         0.459480   0.280294   1.639    0.976
## A.graminis - MULTz yellow : Female - Male == 0            0.157615   0.255872   0.616    1.000
## A.graminis - Multifunnel green : Female - Male == 0       0.573887   0.255872   2.243    0.586
## A.graminis - Bottle green : Female - Male == 0            0.653365   0.626757   1.042    1.000
## A.obscuricollis - Fan-trap green : Female - Male == 0     0.119590   0.313378   0.382    1.000
## A.obscuricollis - Fan-trap yellow : Female - Male == 0   -0.232419   0.290132  -0.801    1.000
## A.obscuricollis - MULTz yellow : Female - Male == 0      -0.195562   0.271394  -0.721    1.000
## A.obscuricollis - Multifunnel green : Female - Male == 0  0.009936   0.271394   0.037    1.000
## A.obscuricollis - Bottle green : Female - Male == 0       0.000000   0.767617   0.000    1.000
## A.biguttatus - Fan-trap green : Female - Male == 0       -0.298750   0.343289  -0.870    1.000
## A.biguttatus - Fan-trap yellow : Female - Male == 0       0.367406   0.361858   1.015    1.000
## A.biguttatus - MULTz yellow : Female - Male == 0         -0.159298   0.327313  -0.487    1.000
## A.biguttatus - Multifunnel green : Female - Male == 0    -0.745248   0.327313  -2.277    0.554
## A.biguttatus - Bottle green : Female - Male == 0         -0.320927   0.767617  -0.418    1.000
## (Adjusted p values reported -- single-step method)
```

3.2.5.2 All species grouped We compare here the captures between the sexes of all species grouped (only for the CRAW and INRAE samples, ie only in Quercus sites, because sex is not available for other sub datasets).

We use a mixed model of the form = `lmer(log1p(Nb_Indiv_90_Days) ~ Trap_Type * Sex+ (1|SiteID) + (1|TreeID), data = tmp)`

The response is the total number of individuals per trap *90 / Number of days of trapping (log(x+1) transformed). We use The Trap Type, Sex and their interaction as fixed effects and the SiteID and TreeID as random effects.

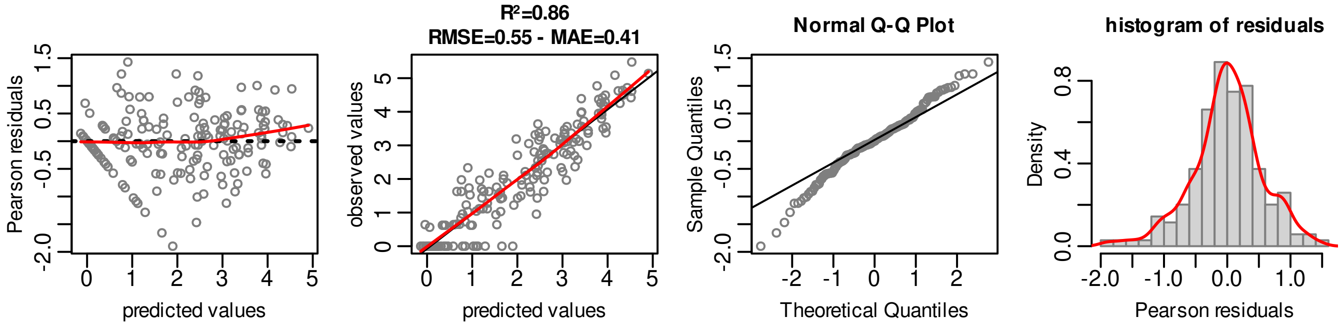

Figure 29:

The Anova table shows no significant effect of the sex nor the interaction of the tree species (Fagus vs Quercus) but no year effect

Table 15: Analysis of Deviance Table (Type II Wald F tests with Kenward-Roger df)

	F	Df	Df.res	Pr(>F)
**Trap_Type**	30.57	4	145.72	0.000000
**Sex**	1.33	1	130.46	0.250743
**Trap_Type:Sex**	0.77	4	130.46	0.547311

The following graph provides a graphical summary of the model.

The gray lines represent traps from the same tree. In red : the average and 95% bootstrap Confidence Interval.

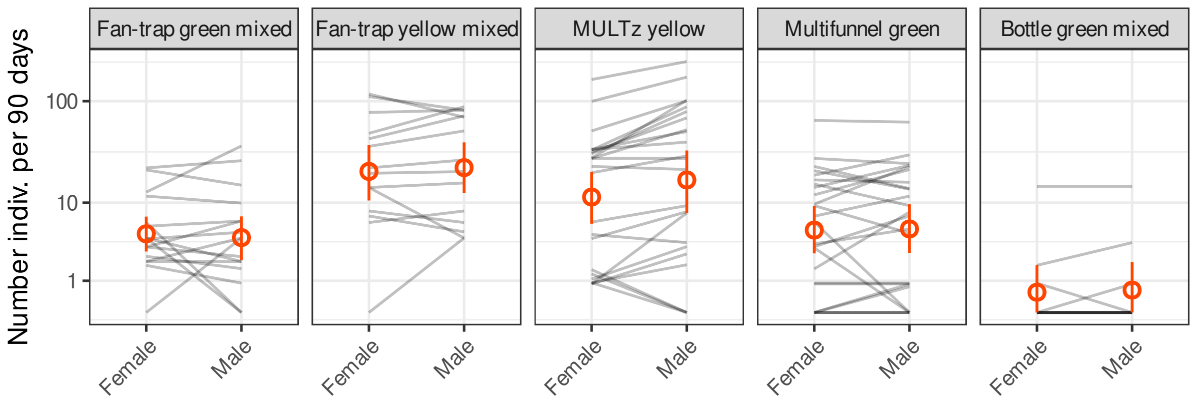
Fan-trap green mixed
Fan-trap yellow mixed
MULTz yellow
Multifunnel green
Bottle green mixed
Number indiv. per 90 days
100
10
1
Female
Male

Figure 30:

3.3 Effect of trap type on number of species caught

3.3.1 Number of species vs number of individuals or trapping duration

There is a clear asymptotic between the number of species and the number of individuals captured.

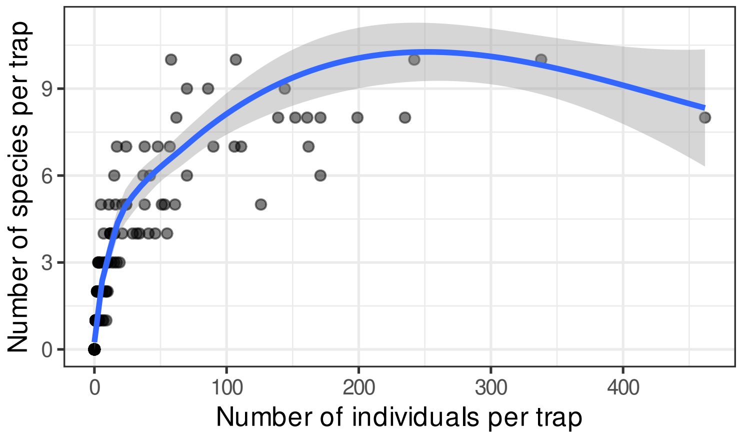

Figure 31:

With a log scale on the x axis the relation becomes linear. . .

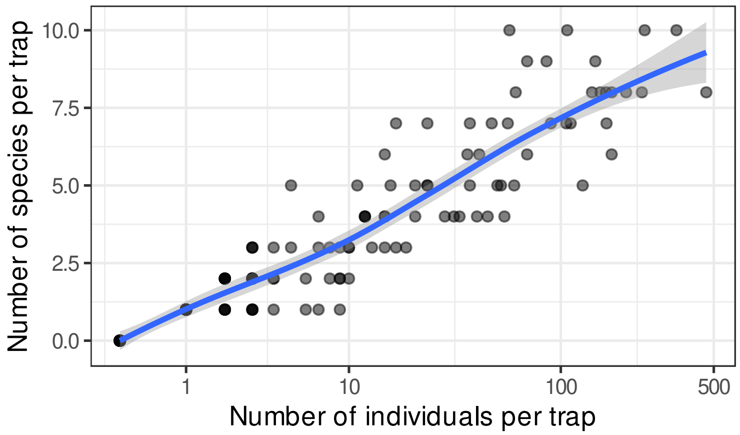

Figure 32:

The pearson correlation coefficient value is large. NB the test here is not strictly speaking valid because the traps are not independent (grouped by sites, tree,. . .)

```
##
##  Pearson's product-moment correlation
##
## data:  log1p(tmp$Nb_Indiv) and tmp$Nb_Sp
## t = 34.244, df = 156, p-value < 2.2e-16
## alternative hypothesis: true correlation is not equal to 0
## 95 percent confidence interval:
##  0.9179765 0.9554486
## sample estimates:
##       cor
## 0.9394609
```

We could fit a linear mixed model but this is a bit overkill as the correlation is very obvious here… NB the random component of the tree is estimated to be 0 and causes boundary warnings –> removed from this model.

```
## Linear mixed model fit by REML ['lmerMod']
## Formula: Nb_Sp ~ log1p(Nb_Indiv) + (1 | SiteID)
##    Data: tmp
##
## REML criterion at convergence: 443
##
## Scaled residuals:
##     Min      1Q  Median      3Q     Max
## -2.4192 -0.4368  0.0920  0.1892  3.9538
##
## Random effects:
##  Groups   Name        Variance Std.Dev.
##  SiteID   (Intercept) 0.05246  0.2290
##  Residual             0.89046  0.9436
## Number of obs: 158, groups:  SiteID, 26
##
## Fixed effects:
##                 Estimate Std. Error t value
## (Intercept)     -0.11613    0.12911  -0.899
## log1p(Nb_Indiv)  1.52998    0.05059  30.244
##
## Correlation of Fixed Effects:
##             (Intr)
## lg1p(Nb_In) -0.723
```

```
## Analysis of Deviance Table (Type II Wald F tests with Kenward-Roger df)
##
## Response: Nb_Sp
##                      F Df Df.res    Pr(>F)
## log1p(Nb_Indiv) 853.73  1 36.321 < 2.2e-16 ***
## ---
## Signif. codes:  0 '***' 0.001 '**' 0.01 '*' 0.05 '.' 0.1 ' ' 1
```

But there is no clear correlation with the number of trapping days.

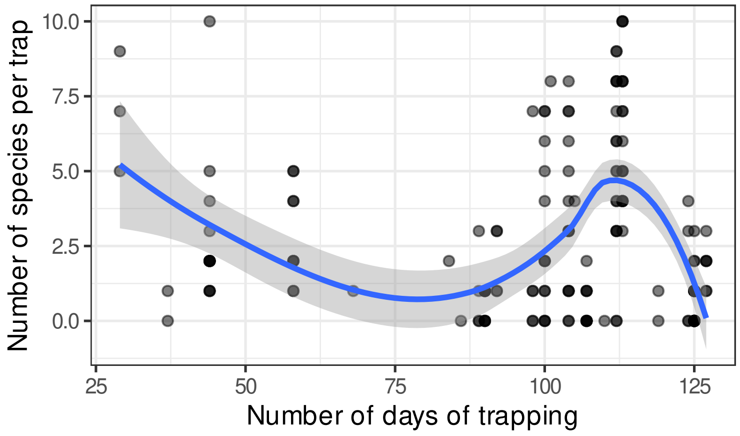

Figure 33:

3.3.2 Number of species vs trap type

We use a mixed model of the form = `lmer(log1p(Nb_Sp) ~ Trap_Type + Tree_Species + Year + (1|SiteID) + (1|TreeID), data = tmp)`

The response is the total number of species per trap (log(x+1) transformed). We use The Trap Type, Tree species (Fagus or Quercus) and Year as fixed effects and the SiteID and TreeID as random effects.

NB : an equivalent Poisson model with number of trapping days as offset fails systematically...

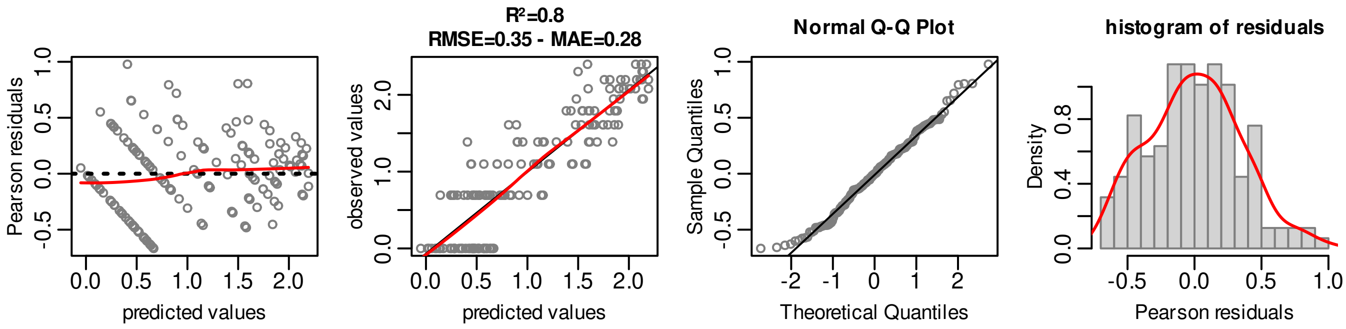

Figure 34:

The Anova table shows a highly significant Trap type effect, and also a more marginal effect of the tree species (Fagus vs Quercus) but no year effect

Table 16: Analysis of Deviance Table (Type II Wald F tests with Kenward-Roger df)

	F	Df	Df.res	Pr(>F)
**Trap_Type**	6.48	4	111.54	0.000100
**Tree_Species**	5.40	1	22.92	0.029415
**Year**	0.72	1	56.15	0.399141

The following graph provides a graphical summary of the model.

The gray lines represent traps from the same tree. In red : the average and 95% bootstrap Confidence Interval. The compact letter display shows which pairwise comparisons are significantly different from each other (with p-value correction).

The green fantraps captured less species on average than the yellow fan-traps, MULTz and Multifunnel green. Note that there is no significant difference between the bottle traps and the fan-traps but this could be due to the fact that these two type of traps where not present the same year so it is difficult to compare them with confidence

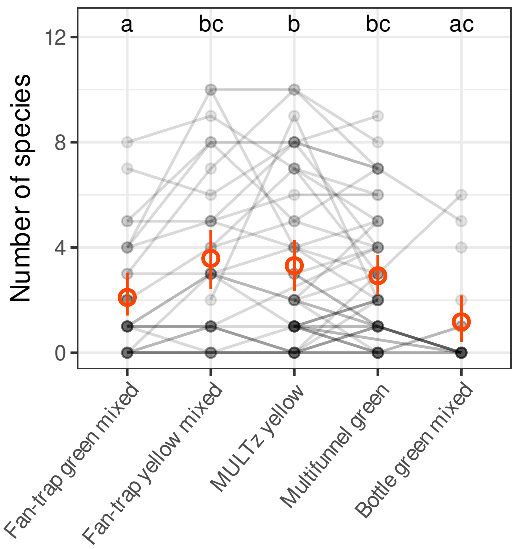

Figure 35:

Here is the detail of the pairwise comparisons :

```
##
##   Simultaneous Tests for General Linear Hypotheses
##
## Multiple Comparisons of Means: Tukey Contrasts
##
##
## Fit: lmer(formula = log1p(Nb_Sp) ~ Trap_Type + Tree_Species + Year +
##     (1 | SiteID) + (1 | TreeID), data = tmp)
##
## Linear Hypotheses:
##                                                   Estimate Std. Error z value Pr(>|z|)
## Fan-trap yellow mixed - Fan-trap green mixed == 0  0.32982    0.10410   3.168  0.01248 *
## MULTz yellow - Fan-trap green mixed == 0           0.38048    0.10150   3.748  0.00156 **
## Multifunnel green - Fan-trap green mixed == 0      0.31784    0.10070   3.156  0.01294 *
## Bottle green mixed - Fan-trap green mixed == 0    -0.03965    0.15565  -0.255  0.99901
## MULTz yellow - Fan-trap yellow mixed == 0          0.05066    0.10041   0.505  0.98613
## Multifunnel green - Fan-trap yellow mixed == 0    -0.01198    0.09962  -0.120  0.99995
## Bottle green mixed - Fan-trap yellow mixed == 0   -0.36947    0.15495  -2.385  0.11330
## Multifunnel green - MULTz yellow == 0             -0.06264    0.08650  -0.724  0.94819
## Bottle green mixed - MULTz yellow == 0            -0.42013    0.13184  -3.187  0.01180 *
## Bottle green mixed - Multifunnel green == 0       -0.35749    0.13452  -2.658  0.05712 .
## ---
## Signif. codes:  0 '***' 0.001 '**' 0.01 '*' 0.05 '.' 0.1 ' ' 1
## (Adjusted p values reported -- single-step method)
```

Summary table with the average number of species caotured per trap type :

Trap_Type	Avg_Nb_Sp	SD
Fan-trap green mixed	2.103	2.144
Fan-trap yellow mixed	3.586	3.179
MULTz yellow	3.310	3.181
Multifunnel green	2.927	2.592
Bottle green mixed	1.176	1.944

3.4 Effect of trap type on the probability of detection of each species

Instead of working with the abundance, we use the presence/absence of each species in each trap over the whole season.

We use a generalized linear mixed model with binomial distribution of the form = `glmer(Presence ~ Trap_Type*Species + (1|SiteID) + (1|TreeID) ,family = binomial)`

We had convergence problems. So the solution we found was 1) to remove the bottle traps form the analysis and 2) change the optimizer and number of iterations

Note that the sampling effort was not uniform for all traps so we should ideally add the number of trapping days as covariate. But when we do that, the model never converges. So we didn't include this co-variate. The previous graphs have shown that the number of sapling days does not seem to be correlated with number of species anyways.

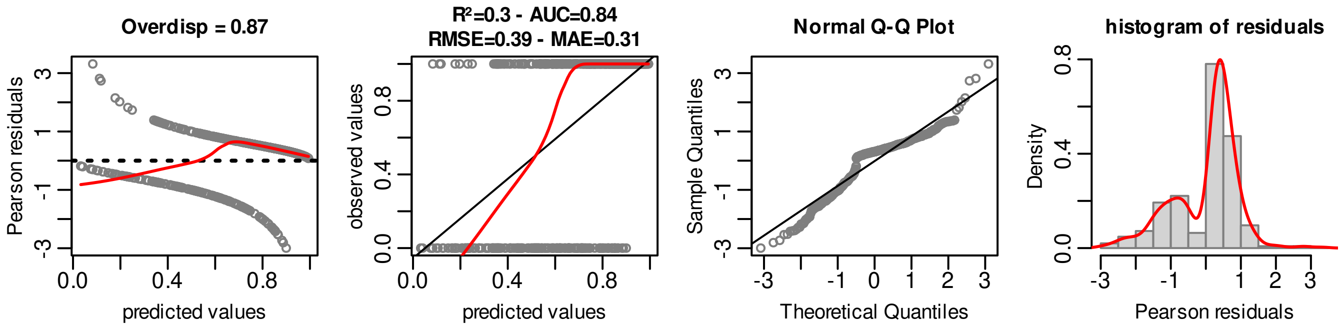

Figure 36:

The analysis of deviance table shows highly significant Trap type and species main effects but no interaction. This results tends to mean that some traps have higher probability to capture the species present and that some of the species are more frequent. But the difference between traps does not depend on the species

Table 18: Analysis of Deviance Table (Type II Wald chisquare tests)

	Chisq	Df	Pr(>Chisq)
**Trap_Type**	37.37	3	0.0000
**Species**	24.42	6	0.0004
**Trap_Type:Species**	18.17	18	0.4442

The flowing graph shows a summary of this model with all pairwise comparisons between traps whatever the species. Each gray line corresponds to one of the 7 most common species. The values are the % of traps in which each of these species has been detected (present at least once during the whole season).

NB : the bottle traps have no letter because we had to remove this treatment from the analysis to avoid model convergence problems…

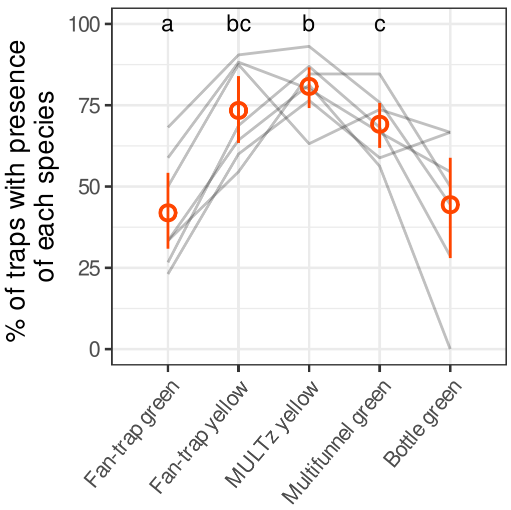

Figure 37:

If we perform all pairwise comparison between traps separately for each species most comparisons are not significant anymore but this is most likely due to a lack of statistical power relative to the grouped model and this is not really necessary because the trap type x species interaction was not significant.

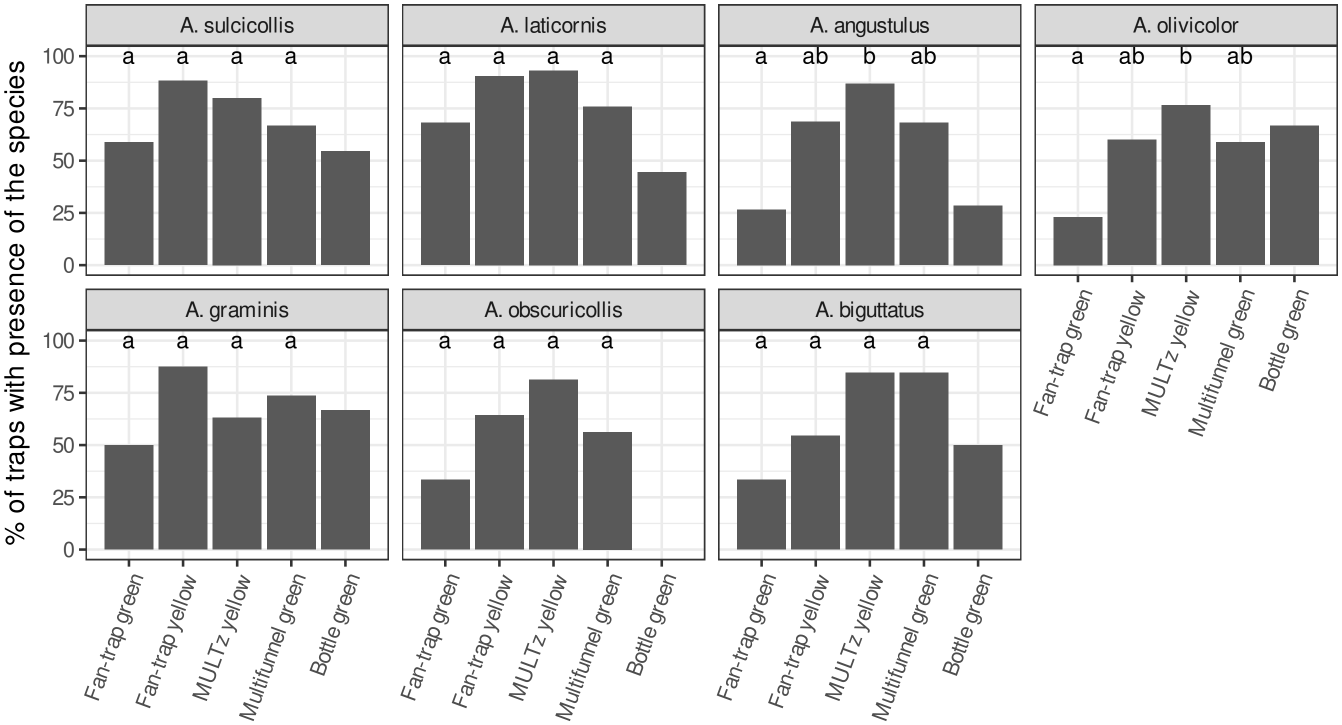

Figure 38:

3.5 How robust are our conclusions if we change the statistical approach or the data ?

Reviewer#1 of our manuscript was worried by the fact that our experimental design was an incomplete randomized bloc design (i.e. not all trap types were present in each tree at each period). He/she also suggested that it might be better to use models with Poisson distribution.

Our dataset is complex and there are plenty of valid ways to analyse it. The most important question is to understand if a different statistical approach would change the general conclusions of the paper.

We perform in this section a robustness analysis : we change the statistical approach, use a subset of the full dataset and see how much it impacts our conclusions.

As suggested by the review we used a subset of the data, keeping only the trees and the dates in which all trap types available for a given year were present (ie we dropped all the trees and dates for which we had missing data). Then we applied various statistical approaches : repeating the analysis of the paper, dropping the bottle traps, performing a separate analysis for each year,... We also replicated the analysis with Poisson or Negative binomial models when possible (these more complex models struggle to converge in some situations).

tl;dr : our conclusions are robust to the statistical approach and to the subset of the data used.

3.5.1 Gaussian Mixed Model on a subset of the data with only the complete blocs

3.5.1.1 Total number of individuals caught .

This is just a replicate of th analysis of the paper but on a subset of the data. We use a mixed model of the form = `lmer(log1p(Nb_Indiv_90_Days) ~ Trap_Type + Tree_Species + Year + (1|SiteID) + (1|TreeID), data = tmp)`

The residual plots show that a gaussian model is a very good approximation of this data

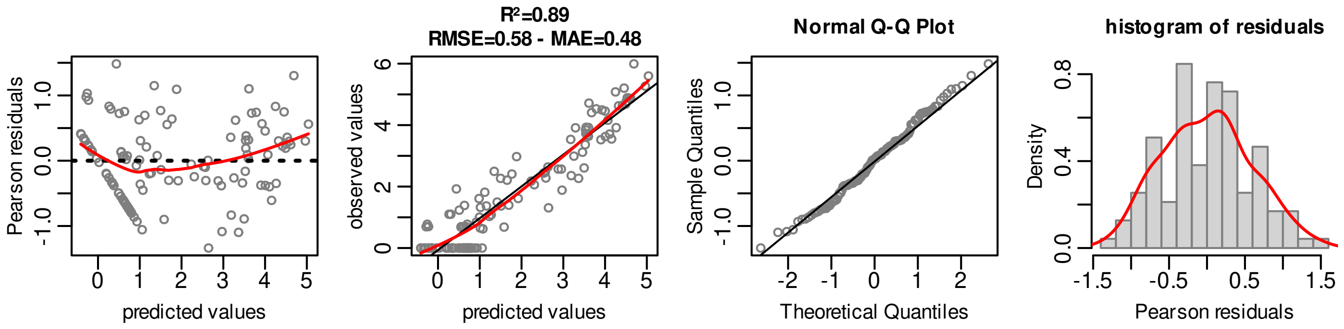

Figure 39:

The Anova table shows a highly significant Trap type effect, and also a more marginal effect of the tree species (Fagus vs Quercus) but no year effect

Table 19: Analysis of Deviance Table (Type II Wald F tests with Kenward-Roger df)

	F	Df	Df.res	Pr(>F)
**Trap_Type**	11.68	4	82.03	0.000000
**Tree_Species**	2.97	1	15.46	0.104671
**Year**	0.00	1	31.27	0.997268

The following graph provides a graphical summary of the model.

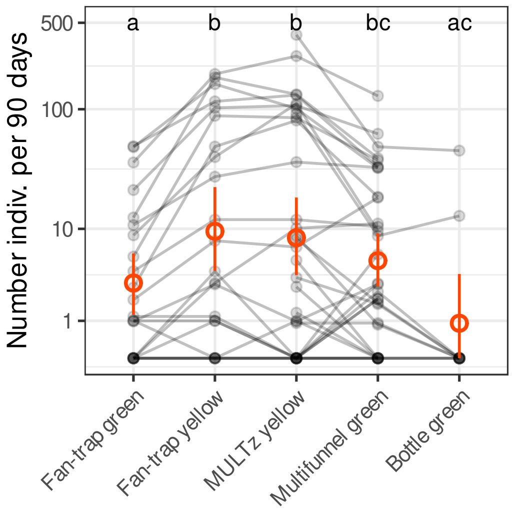

Figure 40:

3.5.1.2 Total number of individuals caught without the bottle traps .

What happens if we drop the bottle traps ?

NB : the comparison between the bottle traps and the fan-traps has higher uncertainty because these traps were never present in the same year and same blocs at the same time. Also the bottle traps captured very few individuals, so we check here what happens if we drop them from the pairwise comparisons.

Table 20: Analysis of Deviance Table (Type II Wald F tests with Kenward-Roger df)

	F	Df	Df.res	Pr(>F)
**Trap_Type**	10.31	3	73.21	0.000010
**Tree_Species**	3.17	1	15.47	0.094792
**Year**	0.01	1	32.94	0.922441

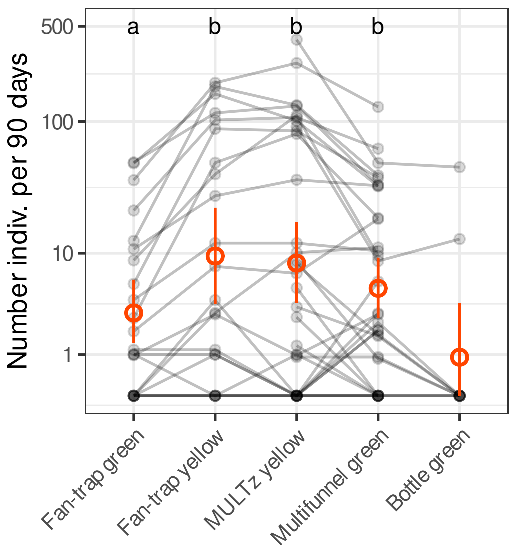

Figure 41:

–> same results

3.5.1.3 Total number of individuals caught and each year analysed separately .

OK but what happens if we analyse both years separately in order to avoid having both the bottle traps and fan-traps involved in the pairwise comparisons while these type of traps were placed only in different years and different sites ?

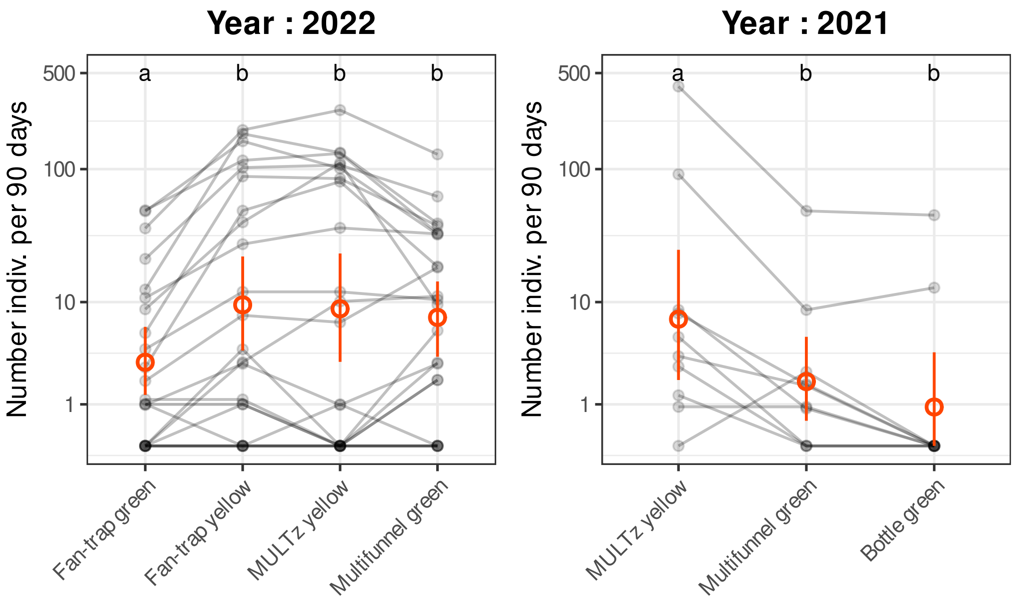

Figure 42:

3.5.2 Poisson/Negative binomial model on a subset of the data with only the complete blocs

Instead of a gaussian model on log transformed counts standardized on 90 days, we could use a Poisson model on the raw counts, with an offset on the log number of trapping days. With the full dataset this kind of model was systematically failing (convergence problem etc). Here we managed to obtain a converging model but only after removing the "year" effect.

3.5.2.1 Total number of individuals caught

The model has the following structure :

`glmer(Nb_Indiv ~ Trap_Type  + Tree_Species + (1|SiteID) + (1|TreeID) + offset(log(Nb_Days)), family = poisson)` However the overdesipersion is rather high : 4.85

We also managed to fit with this subset of the data a negative binomial model which incorporates an overdispersion parameter. The model has the following form :

```
glmer.nb(Nb_Indiv ~ Trap_Type  + Tree_Species + (1|SiteID) + (1|TreeID) + offset(log(Nb_Days)))
```

This model, the Trap_Type effect is highly significant and the Tree_Species effect is not significant (but close to the usual 0.05 significance level) The year effect could not be tested (including it makes the model crash)

Table 21: Analysis of Deviance Table (Type II Wald chisquare tests)

	Chisq	Df	Pr(>Chisq)
**Trap_Type**	84.97	4	0.0000
**Tree_Species**	2.88	1	0.0897

We can then compute all pairwise multiple comparisons as done before and summarize the results on the graph :

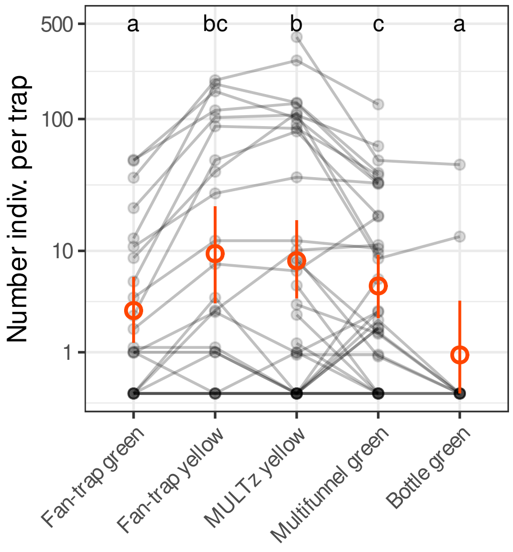

Figure 43:

3.5.2.2 Total number of individuals caught without the bottle traps

OK but what happens if we do not use the bottle traps in the pairwise comparisons ?

This model, the Trap_Type effect is highly significant and the Tree_Species effect is not significant (but close to the usual 0.05 significance level) The year effect could not be tested (including it makes the model crash)

Table 22: Analysis of Deviance Table (Type II Wald chisquare tests)

	Chisq	Df	Pr(>Chisq)
**Trap_Type**	60.10	3	0.0000
**Tree_Species**	2.99	1	0.0836

We can then compute all pairwise multiple comparisons as done before and summarize the results on the graph :

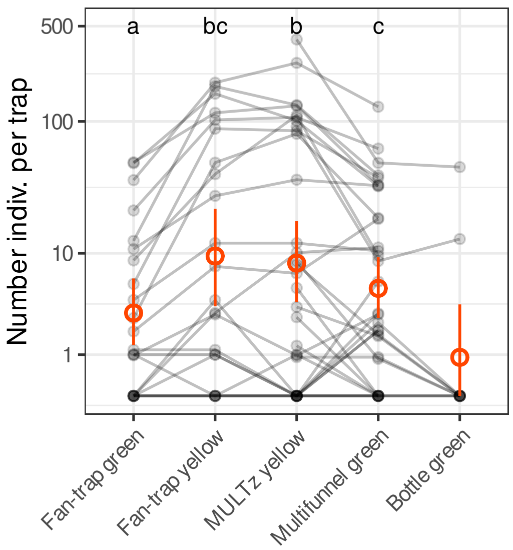

Figure 44:

3.5.2.3 Total number of individuals caught and each year analysed separately .

OK, but what happens if analyse each year separately ??

We tried many approaches but none of the models converged whatever the optimizer, the number of iterations and even by simplifying the model to the bare minimum (no TreeID random effects, no offset, etc.)

3.5.3 Trap catches for different species

3.5.3.1 Gaussian model on a subset of the data with only the complete blocs .

We repeat our approach from the main paper for the comparison of the 7 most common species but on the subset of the data

We use a mixed model of the form (on the subset of data)= `lmer(log1p(Nb_Indiv_90_Days) ~ Trap_Type*Species + (1|SiteID) + (1|TreeID), data = tmp)`

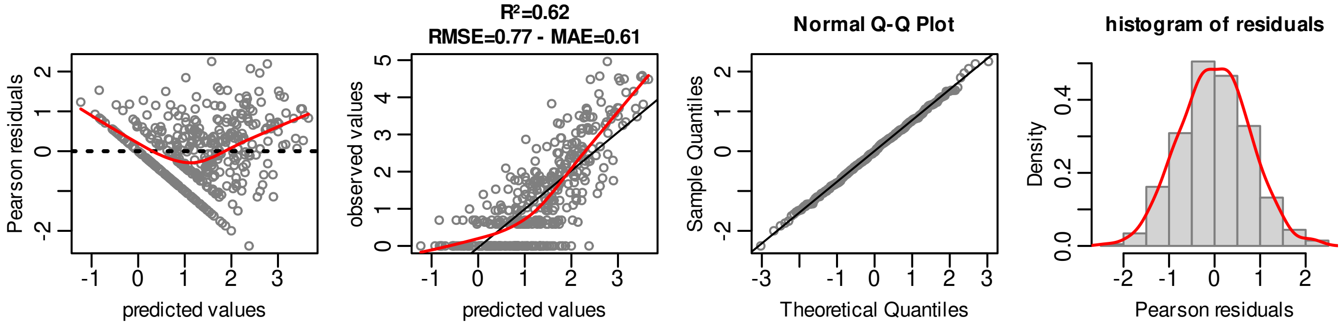

Figure 45:

The Anova table shows a highly significant Trap type x species effect as with the full dataset

Table 23: Analysis of Deviance Table (Type II Wald F tests with Kenward-Roger df)

	F	Df	Df.res	Pr(>F)
**Trap__Type**	33.46	4	357.95	0.000000
**Species**	26.30	6	359.31	0.000000
**Trap__Type:Species**	2.15	24	350.99	0.001623

The following graph provides a graphical summary of the model. The general pattern is very similar to the one obtained with the full dataset, in particular the difference between A.biguttatus and the other species.

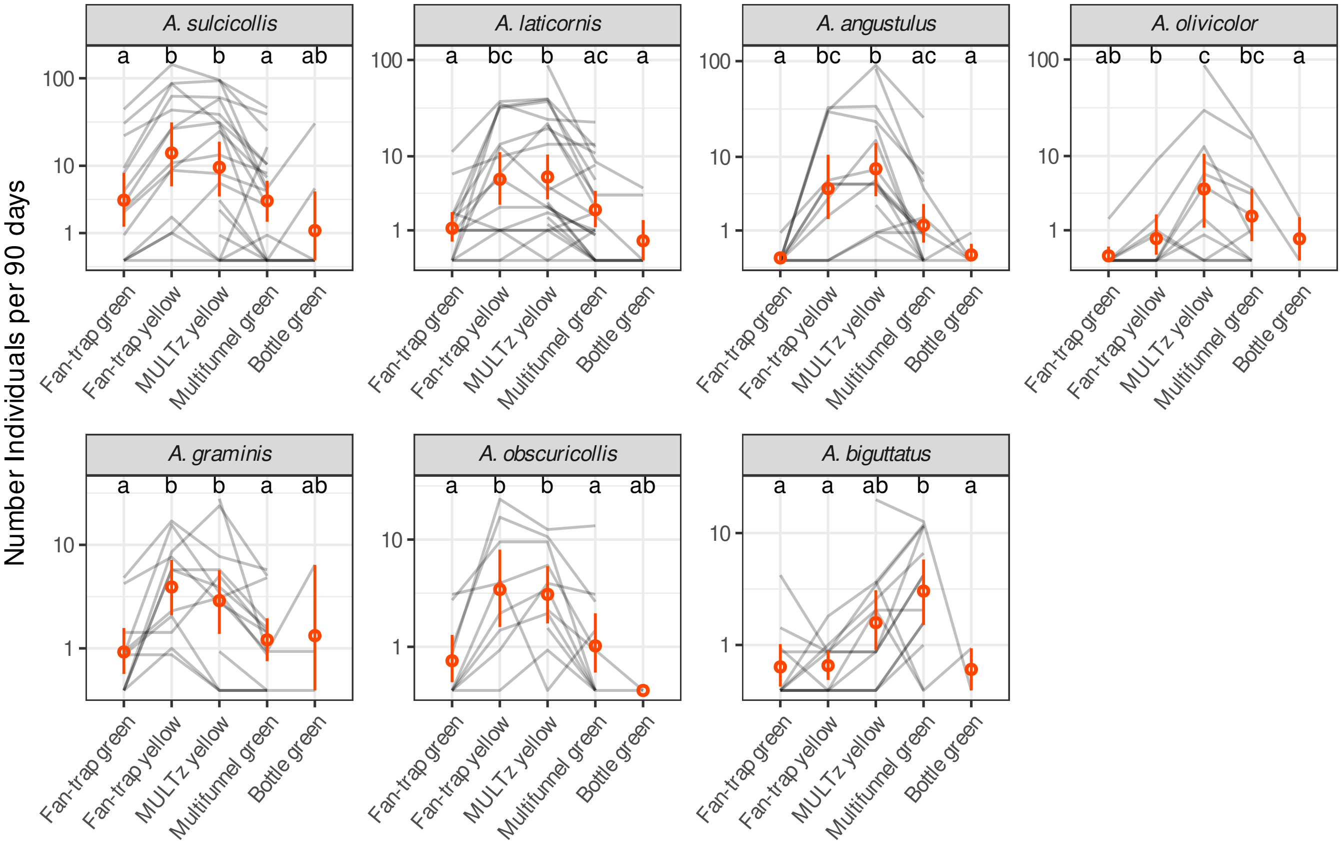

Figure 46:

3.5.3.2 Poisson / Negative binomial model on a subset of the data with only the complete blocs .

A Poisson model fails to converge or is nearly unidentifiable and is overdispersed anyways (overdisp ~6).

A negative binomial model takes ages to compute and finally fails to converge even when changing the optimizer and increasing the number of iteration.

We didn't tested the models for each species separately

3.5.4 General conclusions of the robustness analysis

Globally, whatever the statistical approach and even on a small subset of the full dataset, the patterns are very similar to the ones obtained with the full dataset.

Our conclusions are very robust.

For the total catches :

- Green fan-traps catches are always significantly lower than the yellow fan traps, MULTz and multifunnel
- Yellow fan-traps and MULTz catches are very similar and never significantly different
- Bottle traps captured significantly lower numbers than MULTz

The only case where we can have small differences in the statistical tests depending on the analysis is for the multifunnel traps. The pattern is however always the same : the multifunnel tend to capture slightly

less than the MULTz (and to a lower extend than the yellow fan-traps) but with a lot of variation. As a consequence the difference is statistically significant with some analyses and not with others. In the paper we took a conservative approach and simply declared the the catches tended to be lower but generally not significant.

For the species level analysis the conclusions are also very similar to the gloabl analysis. Most species follow a similar pattern (more captures in the yellow traps) with the notable exception of A. biguttatus which seems to be clearly more attracted by the multifunnel green traps.

4 Examination a few miscellaneous special cases

4.1 "Pale Yellow" Fan-traps

Beside the two main trap colors : vivid "yellow" and "green", a few traps have a "Pale yellow" color. This is due to the fact that the wrong yellow color was used to paint the first traps. So at first the pale yellow and green fan-traps were placed on each tree (2 trees per site) of the 10 sites for ULB team. When the correct yellow paint has become available an additional trap with the same vivid yellow color as for the other teams was placed in all trees along with the pale yellow and green ones.

The pale yellow traps captured only 3 specimens over 8 trapping periods of ~2 weeks for all 10 traps. –> to simplify the experimental design, we have simply dropped these traps in the rest of the analysis

4.2 Does (3Z)-hexanol chemical lure have an effect on the captures ?

We performed a small preliminary test with this chemical lure (not a pheromone though) supposed to be attractive for *Agrilus planipennis*.

This is a small separate experiment on sites different from the rest of the study.

Three multifunnel traps have been baited with (3Z)-hexanol. They have been placed in 3 different sites and paired with a non baited multifunnel trap on the same site (but a different tree). All 6 traps catches have been collected 4 times.

On the following graph, each line relates the trap catches from the same site (sub-graphs) at the same date (ie comparable catches).

There is no obvious differences. However it is rather difficult to conclude with only 3 traps of each. One of the sites had also 0 captures.

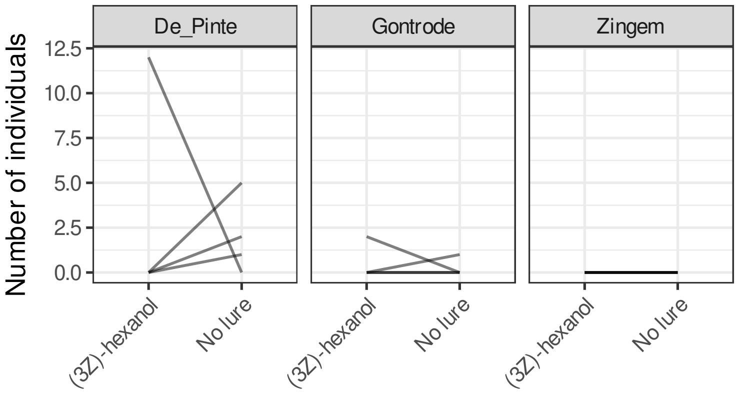

Figure 47:

Very basic linear mixed model of the log(x+1) transformed catches vs type of lure + TrapID as random effect (all more complicated model failed to converge). No significant difference but statistical power very low. . . .

Model : `lmer(log1p(Nb_Indiv) ~ Trap_Lure + (1|TrapID), data = tmp)`

```
## Analysis of Deviance Table (Type II Wald F tests with Kenward-Roger df)
##
## Response: log1p(Nb_Indiv)
##                F Df Df.res Pr(>F)
## Trap_Lure 0.0238  1      4 0.8849
```

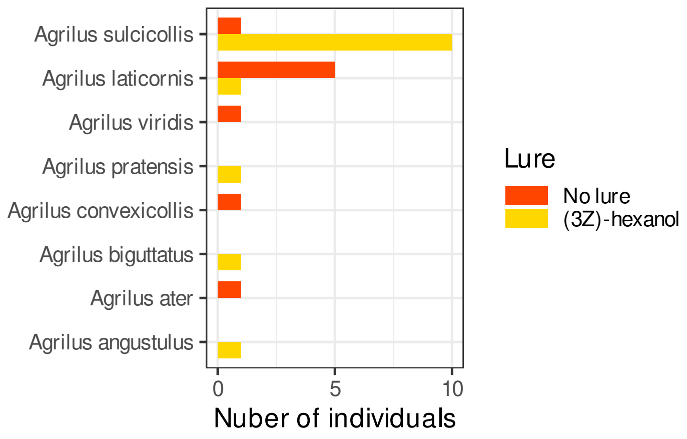

Figure 48:

4.3 Captures in Pear trees orchards

In Pear orchards we captured as expected only the target species : *Agrilus sinuatus.* However we captured only 17 individuals in 2021 and 0 in 2022 despite their presence being attested by visual observations (beating tray).

Note that most of the captures comes from only one of the 5 sites of this year and that many captures happened rather late in the season (end August - September).

The multifunnel traps were received later and trapped during 38 days relative to 101 days for bottle traps and 45-101 days for the MULTz

In 2022 the design was perfectly balanced with 85 days of trapping between early June and end August. The sampling finished only ~ 15 days earlier relative to 2021

SiteID	TreeID	Date	Nb_Days	Species	Trap_Type	Total
Heibergweg straat	4.1	2021-08-23	14	Agrilus sinuatus	Bottle green	1
Heibergweg straat	4.1	2021-09-04	10	Agrilus sinuatus	Bottle green	1
Heibergweg straat	4.1	2021-09-13	7	Agrilus sinuatus	Bottle green	3
Heibergweg straat	4.1	2021-08-13	7	Agrilus sinuatus	MULTz yellow	1
Heibergweg straat	4.1	2021-08-23	14	Agrilus sinuatus	Multifunnel green	2
Heibergweg straat	4.1	2021-09-13	7	Agrilus sinuatus	Multifunnel green	1
Heibergweg straat	4.2	2021-08-23	14	Agrilus sinuatus	Bottle green decoy	2
Heibergweg straat	4.2	2021-08-13	7	Agrilus sinuatus	MULTz yellow	1
Heibergweg straat	4.2	2021-08-23	14	Agrilus sinuatus	MULTz yellow	1
Heibergweg straat	4.3	2021-08-23	14	Agrilus sinuatus	Multifunnel green	1
tuinbouwschool achter	1.1	2021-06-12	10	Agrilus sinuatus	MULTz yellow	1
tuinbouwschool achter	1.2	2021-06-12	10	Agrilus sinuatus	MULTz yellow	1
tuinbouwschool voor	2.1	2021-06-21	7	Agrilus sinuatus	MULTz yellow	1

The differences between trap types seem to be limited. Based on the number of individuals captured corrected by the number of days of trapping , the bottle traps seems less efficient but this is based on very few real catches.

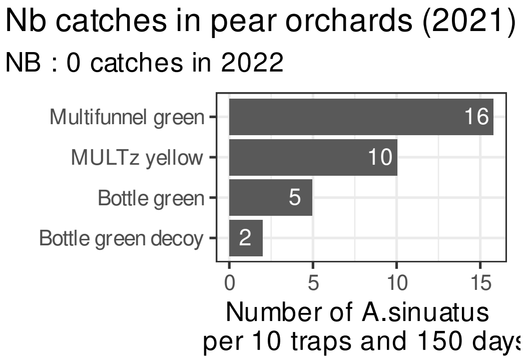

Figure 49:

Trap_Type	Nb_Indiv	Nb_Days	Nb_Indiv_1500_Days
Bottle green	5	1515	4.950
Bottle green decoy	2	1515	1.980
Multifunnel green	4	380	15.789
MULTz yellow	6	898	10.022

Conclusions : based on these 2 years of captures, the usage of traps for the surveillance of *Agrilus sinuatus* in pear orchards seems to be of limited interest while the small size of the trees allows the use of the beating tray for standardized counts.

Because this *Pyrus* dataset is very different form the rest and with very few catches, these pear sites have not not been included in the rest of the analysis.

4.4 Captures on Poplars

Poplars have a rather different fauna (and the sites were more open, not forests).

As expected the 2 most frequent species are poplars specialists : Agrilus pratensis and Agrilus ater. Anthaxia manca is a rare species living on Ulmus spp. (all 10 specimens collected in the same sample)., Agrilus convexicollis is living on Fraxinus excelsior.

Traps were placed on 11 sites but with very uneven sampling intensities. In most of the sites only one or two MULTz or Multifunnel have been placed for a short period of time.

One site in Oud-Heverlee received a full design with fan traps with or without decoys MULTz and multifunnel and the vast majority of catches are coming from that site. Agrilus ater has just been captured in 2 other sites (8 individuals)

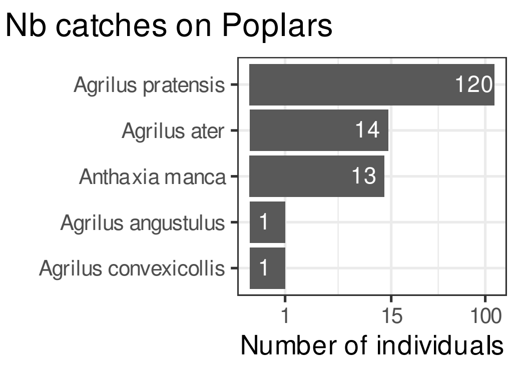

Figure 50:

SiteID	TreeID	TrapID	SampleID	Trap_Type	Species	Total
Envoz	Envoz_53461484	T307	T307_B	MULTz yellow	Agrilus ater	2
Envoz	Envoz_53461484	T306	T306_B	Multifunnel green	Agrilus ater	2
Envoz	Envoz_53461484	T306	T306_D	Multifunnel green	Agrilus ater	1
Mons	Mons_45047669	T323	T323_A	Multifunnel green	Agrilus ater	3
Oud_Heverlee	Oud_Heverlee_T01	Oud_Heverlee_T01_2	ILVO004_B	Fan-trap yellow mixed	Agrilus angustulus	1
Oud_Heverlee	Oud_Heverlee_T01	Oud_Heverlee_T01_2	ILVO004_B	Fan-trap yellow mixed	Agrilus pratensis	3
Oud_Heverlee	Oud_Heverlee_T01	Oud_Heverlee_T01_3	ILVO002_A	MULTz yellow	Agrilus pratensis	20
Oud_Heverlee	Oud_Heverlee_T01	Oud_Heverlee_T01_3	ILVO002_A	MULTz yellow	Anthaxia manca	1
Oud_Heverlee	Oud_Heverlee_T01	Oud_Heverlee_T01_3	ILVO002_D	MULTz yellow	Agrilus ater	1
Oud_Heverlee	Oud_Heverlee_T01	Oud_Heverlee_T01_4	ILVO001_A	Multifunnel green	Agrilus pratensis	87
Oud_Heverlee	Oud_Heverlee_T01	Oud_Heverlee_T01_4	ILVO001_A	Multifunnel green	Anthaxia manca	10
Oud_Heverlee	Oud_Heverlee_T01	Oud_Heverlee_T01_4	ILVO001_B	Multifunnel green	Agrilus pratensis	7
Oud_Heverlee	Oud_Heverlee_T01	Oud_Heverlee_T01_4	ILVO001_B	Multifunnel green	Anthaxia manca	2
Oud_Heverlee	Oud_Heverlee_T01	Oud_Heverlee_T01_4	ILVO001_C	Multifunnel green	Agrilus ater	2
Oud_Heverlee	Oud_Heverlee_T01	Oud_Heverlee_T01_4	ILVO001_D	Multifunnel green	Agrilus ater	3
Oud_Heverlee	Oud_Heverlee_T02	Oud_Heverlee_T02_2	ILVO008_B	Fan-trap yellow mixed	Agrilus pratensis	1
Oud_Heverlee	Oud_Heverlee_T02	Oud_Heverlee_T02_4	ILVO003_A	Multifunnel green	Agrilus pratensis	1
Oud_Heverlee	Oud_Heverlee_T03	Oud_Heverlee_T03_3	ILVO006_A	MULTz yellow	Agrilus convexicollis	1
Oud_Heverlee	Oud_Heverlee_T03	Oud_Heverlee_T03_3	ILVO006_A	MULTz yellow	Agrilus pratensis	1

4.5 Captures in Canada

As expected the Canadian species composition is rather different than the European one. The number of replicates was also not very high in Canada. As a consequence it was not always desirable to include the Canadian data in the global analysis. It is nevertheless very interesting to see how the fan-traps compare to the traditional multifunnel green with a completely different fauna.

There was only 3 different traps in 3 different trees (9 traps in total).

For the total number of captures, the results are more or less in line with what was observed in Europe : the green fan-traps capture less while the yellow fan-traps are equivalent to the green multifunnel. So the yellow fan-traps are also clearly attractive for the non European fauna.

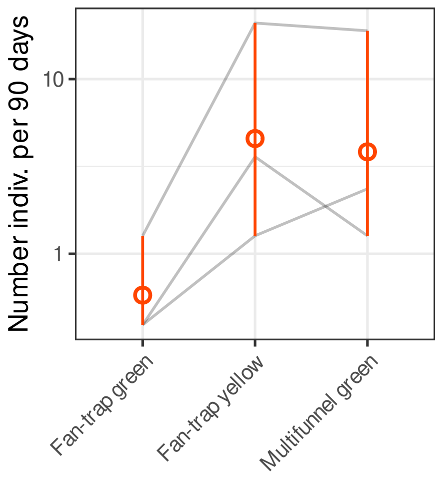

Figure 51:

The total number of captures per species is not very large so the species by species comparison is not easy.

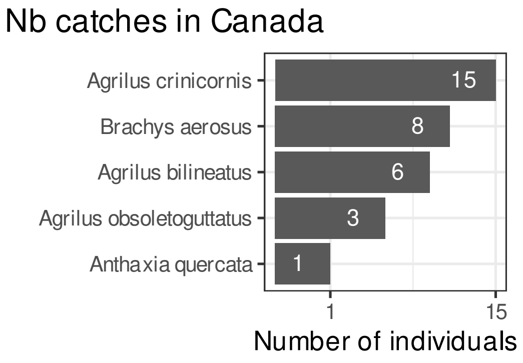

Figure 52:

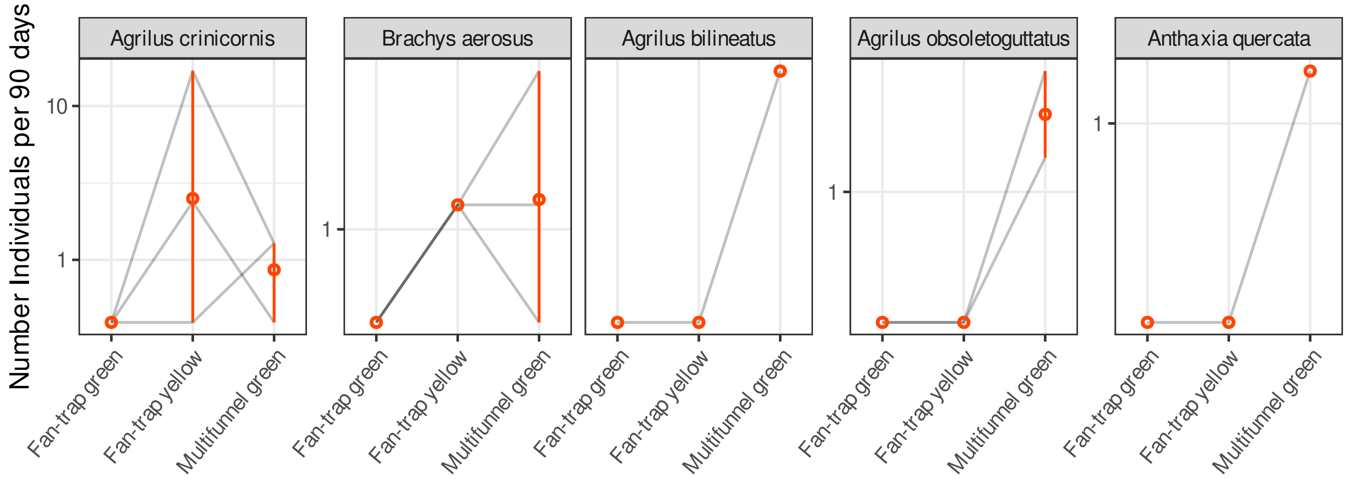

Figure 53:

5 Phenology

5.1 Total catches

Each gray line represents the same trap

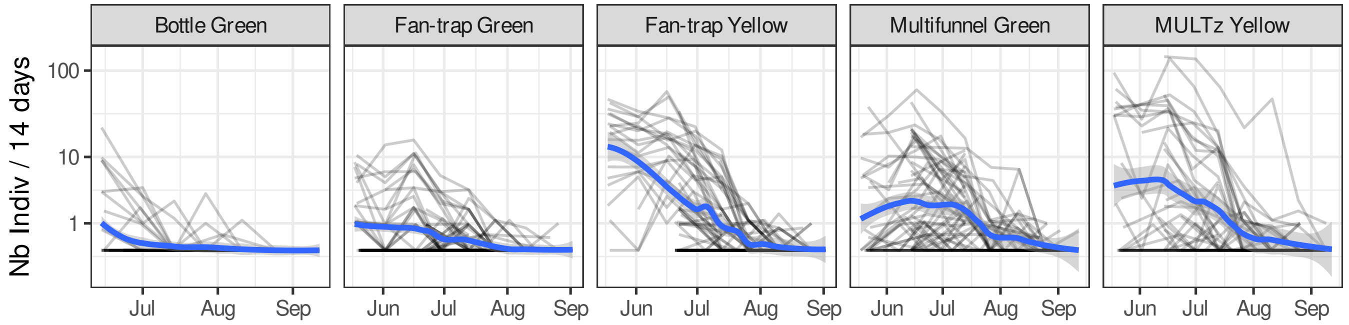

Figure 54:

5.2 Phenology of the most common species

Each line represent the average for each site of the number of beetles captured per 14 days (average of all the traps for this site at a given date).

A.sulcicollis and A.biguttatus seem to fly a bit earlier than the other species

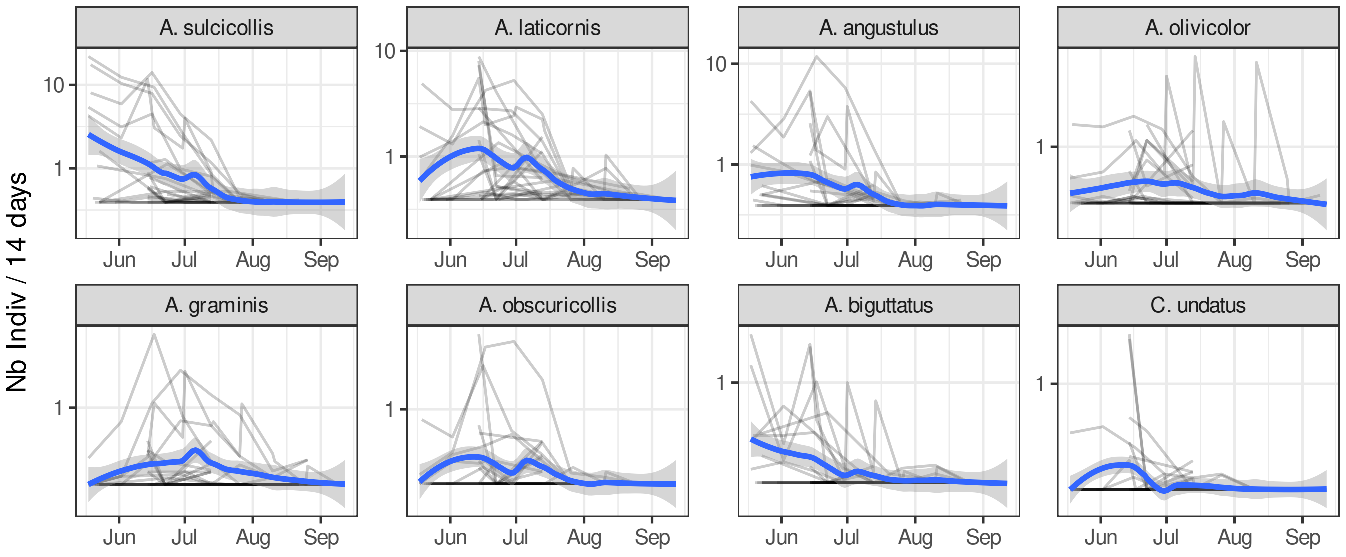

Figure 55:

The previous graph might be not so easy to read and we are more accustomed to bar graphs to represent the phenology. This is not so easy here for 2 reasons : 1) the sampling effort is not constant over time so we need to correct for that but there are different ways to do so and 2) we need to regroup the observations per bins (ie we need to discretise the the sampling date). The problem is that each sample is itself a time period of ~14 days but they are not all the same on all sites.

We make a tentative on the following graph :

1) We divided the number of individuals captures for each species by the number of trapping days, then we averaged that over all sites and all traps for each period
2) We took the median of the sampling date (mid date between the end and start of the sampling session) and attributed it to a period corresponding to the beginning or the end of each month (2 periods per month)

We can clearly see that A.sulcicollis and A.biguttatus were already at their peak (or had even passed it) when we started our sampling while the other species peaked during our sampling period.

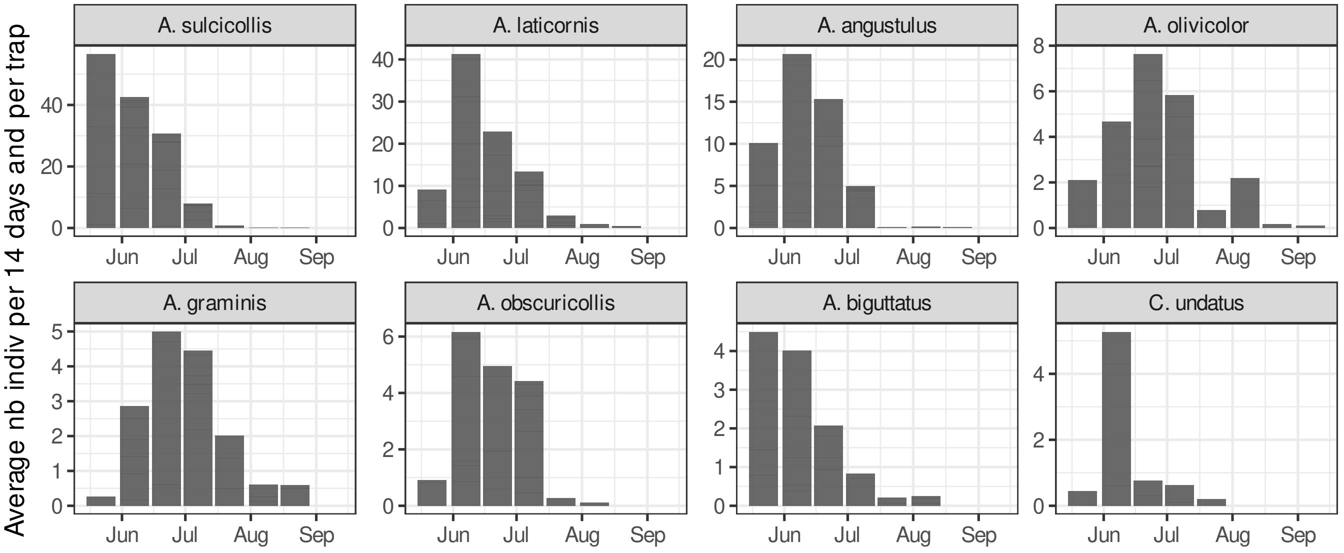

Figure 56:

NB : In 2022, the sampling started 15 days earlier in the oak tree sites from Wallonia (the Belgian sites where we captured the most beetles) than in France. This could explain why the relative number of A.sulcicollis is much lower in France : we probably miss the peak of this early species

Country	SiteID	Year	Date_Start	Date_End
Belgium	Sclassin	2021	2021-05-20	2021-09-16
Belgium	Libin	2021	2021-06-04	2021-09-16
Belgium	Rochefort	2021	2021-06-04	2021-09-16
Belgium	Haute Bodeux	2021	2021-06-07	2021-09-15
Belgium	Spa	2021	2021-06-07	2021-09-15
Belgium	Tenneville_E	2021	2021-06-08	2021-09-23
Belgium	Uccle_Forêt de Soignes	2022	2022-05-10	2022-09-12
Belgium	Tenneville	2022	2022-05-11	2022-09-13
Belgium	Finneveaux	2022	2022-05-12	2022-09-02
Belgium	Jamblinne	2022	2022-05-12	2022-09-02
Belgium	Rochefort	2022	2022-05-12	2022-09-02
Belgium	Custinne	2022	2022-05-13	2022-09-02
Belgium	Houyet	2022	2022-05-13	2022-09-02
France	Vierzon_11	2022	2022-05-31	2022-07-28
France	Vierzon_179	2022	2022-05-31	2022-07-28
France	Vierzon_236	2022	2022-05-31	2022-07-28
France	Vierzon_249	2022	2022-05-31	2022-07-28
France	Vierzon_70	2022	2022-05-31	2022-07-28
France	Vierzon_71	2022	2022-05-31	2022-07-28
France	Vierzon_234	2022	2022-06-14	2022-07-28

6 Spectral analysis of the traps color (reflectance spectra)

Notes :

- Reflectance is a unit less ratio between the quantity of light reflected and the incident quantity
- Measured on a ASD FieldSpec4 spectrometer (Malvern Panalytical, Malvern, United Kingdom)
- The measures have been performed in 2023, on traps that have been placed during several season outside. So their color might have changed a little bit relative to brand new traps.
- We present the average of 5 reflectance measures
- The yellow color used on the graphs is different from the true yellow color of the traps because the true color is too bright and difficult to see on the graphs.
- The green color used on the graphs is relatively similar to the one of the traps
- We added a small color gradient scale showing the theoretical correspondence between wavelengths and "standard" human vision colors

The Multz and Multifunnel are commercial traps with a predetermined color. The fan-traps and bottle traps were painted with two different paints/colors :

- Green paint similar to the the multifunnel green (RAL 6038)
- The yellow paint (yellow fluor spray layer - Motip article number 04022, EAN 8711347040223) was applied over a first layer of plastic primer and a second layer of matt white (all products from Motip, Wolvega, The Netherlands).

Raw values from the spectrometer :

Wavelength corresponding to the maximum Reflectance :

Trap_Type	Wavelength	Reflectance
Bottle-trap green	515	0.418
Fan-trap green	515	0.651
Fan-trap yellow	527	1.234
Multifunnel green	528	0.625
MULTz yellow	545	1.135

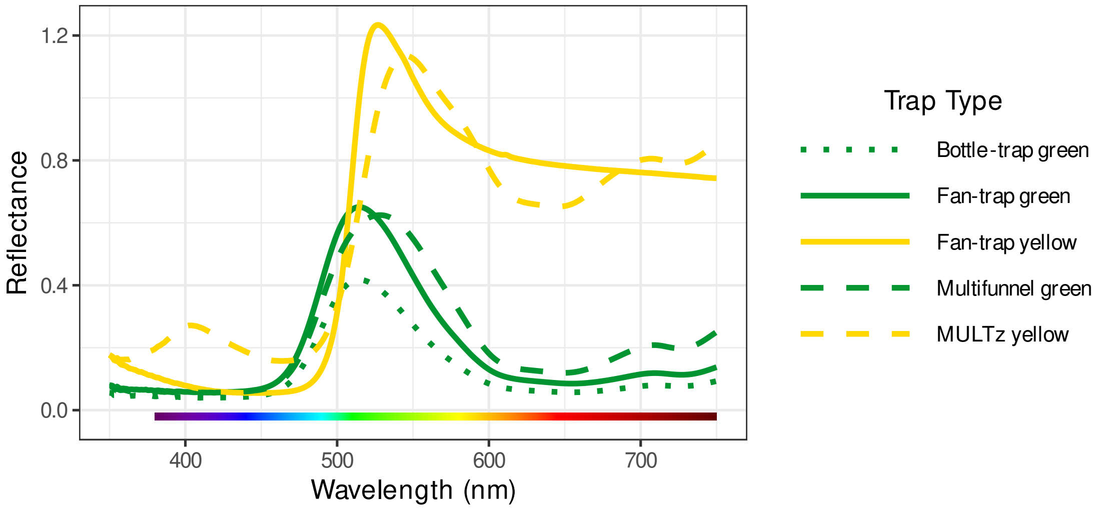

Figure 57:

Corrected values.

We added the absolute value of the minimum when it is negative, then we divided by the maximum reflectance value.

NB: this is similar to the options fixneg = “addmin” and opt=“maximum” in the function pavo::procspec (but we didn’t used that package to reduce unnecessary dependances).

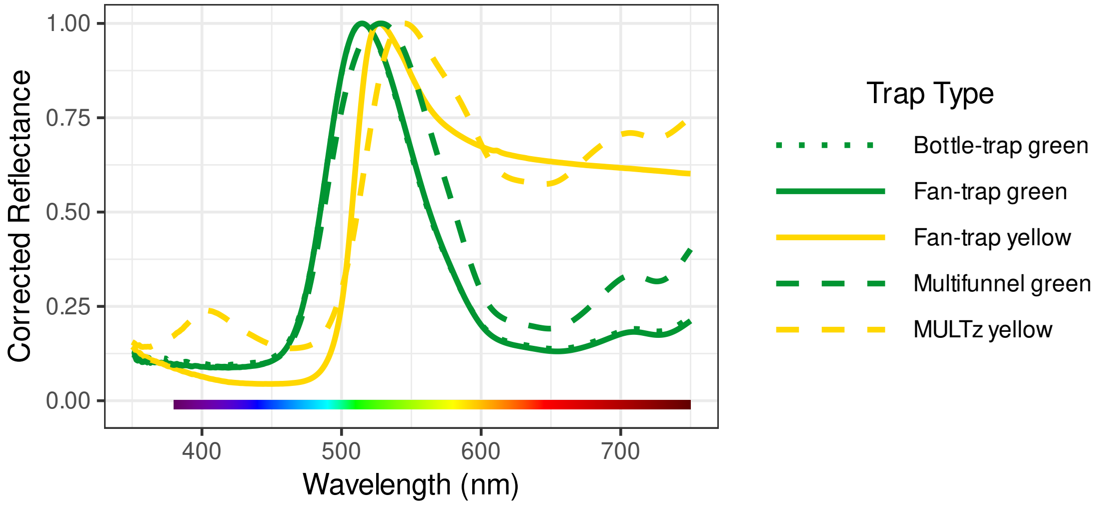

Figure 58:

The variability between replicates is very low (one line = 1 replicate)

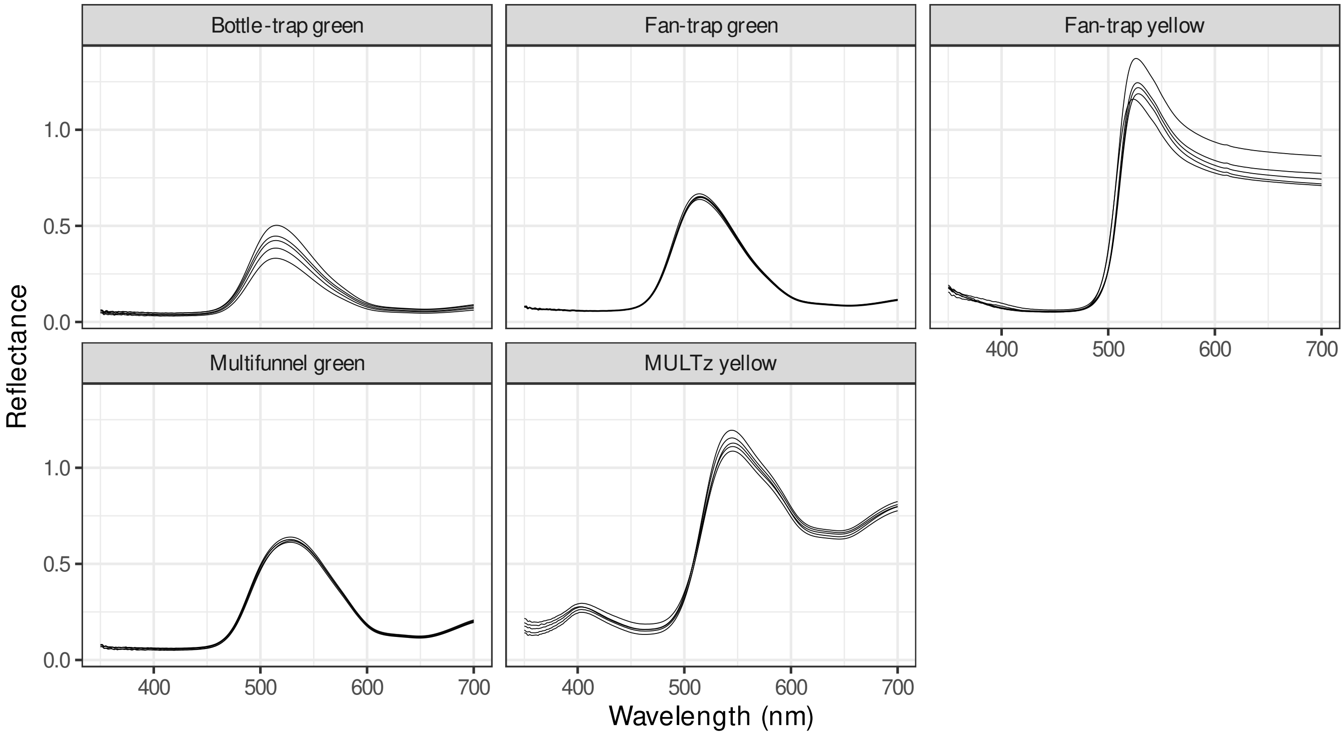

Figure 59:

7 Session Info

```
## R version 4.3.2 (2023-10-31)
## Platform: x86_64-pc-linux-gnu (64-bit)
## Running under: Ubuntu 22.04.3 LTS
##
## Matrix products: default
## BLAS:   /usr/lib/x86_64-linux-gnu/openblas-pthread/libblas.so.3
## LAPACK: /usr/lib/x86_64-linux-gnu/openblas-pthread/libopenblasp-r0.3.20.so;  LAPACK version 3.10.0
##
## locale:
##  [1] LC_CTYPE=en_GB.UTF-8       LC_NUMERIC=C               LC_TIME=en_GB.UTF-8        LC_COLLATE=en_GB.UTF-8
##  [5] LC_MONETARY=en_GB.UTF-8    LC_MESSAGES=fr_BE.UTF-8    LC_PAPER=fr_BE.UTF-8       LC_NAME=C
##  [9] LC_ADDRESS=C               LC_TELEPHONE=C             LC_MEASUREMENT=fr_BE.UTF-8 LC_IDENTIFICATION=C
##
## time zone: Europe/Brussels
## tzcode source: system (glibc)
##
## attached base packages:
## [1] stats     graphics  grDevices utils     datasets  methods   base
##
## other attached packages:
##  [1] patchwork_1.1.3  dplyr_1.1.3      tidyr_1.3.0      ggspatial_1.1.9  sf_1.0-14        taxize_0.9.100   car_3.1-2
##  [8] carData_3.0-5    multcomp_1.4-25  TH.data_1.1-2    MASS_7.3-60      survival_3.5-7   mvtnorm_1.2-3    lme4_1.1-34
## [15] Matrix_1.6-1.1   ggplot2_3.4.4    kableExtra_1.3.4 pander_0.6.5     knitr_1.44
##
## loaded via a namespace (and not attached):
##  [1] DBI_1.1.3          gridExtra_2.3      sandwich_3.0-2     rlang_1.1.1        magrittr_2.0.3     e1071_1.7-13
##  [7] compiler_4.3.2     mgcv_1.9-0         maps_3.4.1         systemfonts_1.0.5  vctrs_0.6.4        rvest_1.0.3
## [13] stringr_1.5.0      httpcode_0.3.0     pkgconfig_2.0.3    crayon_1.5.2       fastmap_1.1.1      backports_1.4.1
## [19] labeling_0.4.3     utf8_1.2.4         rmarkdown_2.25     nloptr_2.0.3       purrr_1.0.2        xfun_0.40
## [25] jsonlite_1.8.7     uuid_1.1-1         broom_1.0.5        parallel_4.3.2     cluster_2.1.5      R6_2.5.1
## [31] stringi_1.7.12     boot_1.3-28.1      rpart_4.1.19       Rcpp_1.0.11        bookdown_0.35      iterators_1.0.14
## [37] zoo_1.8-12         triebeard_0.4.1    base64enc_0.1-3    splines_4.3.2      nnet_7.3-19        tidyselect_1.2.0
## [43] rstudioapi_0.15.0  abind_1.4-5        yaml_2.3.7         codetools_0.2-19   curl_5.1.0         lattice_0.22-5
## [49] tibble_3.2.1       withr_2.5.1        evaluate_0.22      foreign_0.8-85     units_0.8-4        proxy_0.4-27
## [55] xml2_1.3.5         pillar_1.9.0       KernSmooth_2.23-22 checkmate_2.2.0    foreach_1.5.2      generics_0.1.3
## [61] munsell_0.5.0      scales_1.2.1       minqa_1.2.6        class_7.3-22       glue_1.6.2         Hmisc_5.1-1
## [67] tools_4.3.2        data.table_1.14.8  webshot_0.5.5      forcats_1.0.0      grid_4.3.2         bold_1.3.0
## [73] ape_5.7-1          urltools_1.7.3     colorspace_2.1-0   nlme_3.1-163       htmlTable_2.4.1    conditionz_0.1.0
## [79] Formula_1.2-5      cli_3.6.1          fansi_1.0.5        viridisLite_0.4.2  svglite_2.1.1      gtable_0.3.4
## [85] digest_0.6.33      classInt_0.4-10    pbkrtest_0.5.2     crul_1.4.0         htmlwidgets_1.6.2  farver_2.1.1
## [91] htmltools_0.5.6.1  lifecycle_1.0.3    httr_1.4.7
```

8 References

Automatic citation of R and all packages used :

R
R Core Team (2023). *R: A Language and Environment for Statistical Computing*. R Foundation for Statistical Computing, Vienna, Austria. https://www.R-project.org/.

car
Fox J, Weisberg S (2019). *An R Companion to Applied Regression*, Third edition. Sage, Thousand Oaks CA. https://socialsciences.mcmaster.ca/jfox/Books/Companion/.

carData
Fox J, Weisberg S, Price B (2022). *carData: Companion to Applied Regression Data Sets*. R package version 3.0-5, https://CRAN.R-project.org/package=carData.

dplyr
Wickham H, François R, Henry L, Müller K, Vaughan D (2023). *dplyr: A Grammar of Data Manipulation*. R package version 1.1.3, https://CRAN.R-project.org/package=dplyr.

ggplot2
Wickham H (2016). *ggplot2: Elegant Graphics for Data Analysis*. Springer-Verlag New York. ISBN 978-3-319-24277-4, https://ggplot2.tidyverse.org.

ggspatial
Dunnington D (2023). *ggspatial: Spatial Data Framework for ggplot2*. R package version 1.1.9, https://CRAN.R-project.org/package=ggspatial.

kableExtra
Zhu H (2021). *kableExtra: Construct Complex Table with 'kable' and Pipe Syntax*. R package version 1.3.4, https://CRAN.R-project.org/package=kableExtra.

knitr
Xie Y (2023). *knitr: A General-Purpose Package for Dynamic Report Generation in R*. R package version 1.44, https://yihui.org/knitr/.

Xie Y (2015). *Dynamic Documents with R and knitr*, 2nd edition. Chapman and Hall/CRC, Boca Raton, Florida. ISBN 978-1498716963, https://yihui.org/knitr/.

Xie Y (2014). "knitr: A Comprehensive Tool for Reproducible Research in R." In Stodden V, Leisch F, Peng RD (eds.), *Implementing Reproducible Computational Research*. Chapman and Hall/CRC. ISBN 978-1466561595.

lme4
Bates D, Mächler M, Bolker B, Walker S (2015). "Fitting Linear Mixed-Effects Models Using lme4." *Journal of Statistical Software*, *67*(1), 1-48. doi:10.18637/jss.v067.i01 https://doi.org/10.18637/jss.v067.i01.

MASS
Venables WN, Ripley BD (2002). *Modern Applied Statistics with S*, Fourth edition. Springer, New York. ISBN 0-387-95457-0, https://www.stats.ox.ac.uk/pub/MASS4/.

Matrix
Bates D, Maechler M, Jagan M (2023). *Matrix: Sparse and Dense Matrix Classes and Methods*. R package version 1.6-1.1, https://CRAN.R-project.org/package=Matrix.

multcomp
Hothorn T, Bretz F, Westfall P (2008). "Simultaneous Inference in General Parametric Models." *Biometrical Journal*, *50*(3), 346-363.

mvtnorm
Genz A, Bretz F (2009). *Computation of Multivariate Normal and t Probabilities*, series Lecture Notes in Statistics. Springer-Verlag, Heidelberg. ISBN 978-3-642-01688-2.

pander
Daróczi G, Tsegelskyi R (2022). *pander: An R 'Pandoc' Writer*. R package version 0.6.5, https://CRAN.R-project.org/package=pander.

patchwork
Pedersen T (2023). *patchwork: The Composer of Plots*. R package version 1.1.3, https://CRAN.R-project.org/package=patchwork.

sf
Pebesma E, Bivand R (2023). *Spatial Data Science: With applications in R*. Chapman and Hall/CRC. doi:10.1201/9780429459016 https://doi.org/10.1201/9780429459016, https://r-spatial.org/book/.

Pebesma E (2018). "Simple Features for R: Standardized Support for Spatial Vector Data." *The R Journal*, *10*(1), 439-446. doi:10.32614/RJ-2018-009 https://doi.org/10.32614/RJ-2018-009, https://doi.org/10.32614/RJ-2018-009.

survival
Therneau T (2023). *A Package for Survival Analysis in R*. R package version 3.5-7, https://CRAN.R-project.org/package=survival.

Terry M. Therneau, Patricia M. Grambsch (2000). *Modeling Survival Data: Extending the Cox Model*. Springer, New York. ISBN 0-387-98784-3.

taxize
Scott Chamberlain, Eduard Szocs (2013). "taxize - taxonomic search and retrieval in R." *F1000Research*. https://f1000research.com/articles/2-191/v2.

Chamberlain S, Szoecs E, Foster Z, Arendsee Z, Boettiger C, Ram K, Bartomeus I, Baumgartner J, O'Donnell J, Oksanen J, Tzovaras BG, Marchand P, Tran V, Salmon M, Li G, Grenié M (2020). *taxize: Taxonomic information from around the web*. R package version 0.9.98, https://github.com/ropensci/taxize.

TH.data
Hothorn T (2023). *TH.data: TH's Data Archive*. R package version 1.1-2, https://CRAN.R-project.org/package=TH.data.

tidyr
Wickham H, Vaughan D, Girlich M (2023). *tidyr: Tidy Messy Data*. R package version 1.3.0, https://CRAN.R-project.org/package=tidyr.
