# Supplementary material for: Enhancing Buprestidae monitoring in Europe: Trap catches increase with a fluorescent yellow colour but not with the presence of decoys
Source: PLoS One. 2024 Jul 18;19(7):e0307397. doi: 10.1371/journal.pone.0307397 (PMC11257278; doi:10.1371/journal.pone.0307397)
Supplement: Attachment — Submitted filename: Response to Reviewers.docx [file pone.0307397.s003.docx]

**Response to reviewers**

**Reviewer #1:** My review is presented below. I attached the supplementary excel file (with data issues presented - for details see review). Unfortunately, I had to reject the manuscript, because I found major issues related to the experimental design, data handling and statistical analyses. Relative to English - there are some minor issues related to grammar and style (as far as I could find as a non-native speaker), which are also listed in my review.

General answer to reviewer #1

We appreciate the reviewer's efforts in scrutinising our paper, particularly their focus on our raw data. Our commitment to making our dataset openly available, a practice that is uncommon in many studies, allows other scientists to realize the challenges of translating idealised designs into practical field conditions, which may remain hidden if the data are not made publicly available.

The main concern of reviewer #1 seems to be that our block design is incomplete (i.e. that all trap types are not always present in all blocks/trees at the same time). We agree that there are some divergences between the ideal sampling design that we had in mind at the beginning and the field reality. This was shown in a totally transparent manner in the supplements (already in the first pages) and the raw data (that will be made publicly available on publication along with the R code). It is difficult to really explain all the methodological details in the core of a manuscript. However, our open data and open science approach allow the reader to dig as deep as they want: the paper gives an overview (with necessary shortcuts), the supplements give much more details and the raw data + R script allow an exact replication of our analyses.

We have modified the main paper to make it clearer that there were divergences between the planned design and the field reality (lines 215-220).

We believe that this kind of divergences between the theory and the field are difficult to avoid unless one set-up the traps in small numbers in only one or two sites. The problem with this approach is that it is more difficult to extrapolate the results to other sites, other conditions, other species,... In this project, we wanted to evaluate the different trap designs in a broad range of conditions, which meant that several teams had to collaborate and that the design was not always perfectly uniform. We also wish to underline that while having a complete and balanced block design was essential at the time of ANOVA, with modern mixed models we have a statistical tool that is much more robust (and partially built to handle) unequal sample sizes and more complex designs.

However, the most important question here is to understand how much these design imbalances (and some statistical choices) impact the main conclusions of this paper (the most important conclusions being that we found no effect of the decoys on our traps and that the yellow colour seems to be more attractive for several European oak *Agrilus* species).

The impact on the decoy vs no decoy comparison is very limited because we always had pairs of traps (bottle or fan-traps) with or without decoys placed very close to each other or even on top of each other in the same tree. Usually when one trap of one pair was missing, the other was missing too.

The impact on our conclusions concerning the colours (higher catches with yellow traps) should also be relatively limited. The multi-funnel and MULTz differ by their colour but also by their design so it is difficult to attribute the differences in catches only to their difference of colour, as explained in the discussion (even if it most likely contributes to the differences). The only traps that are directly comparable in terms of colour alone are the fan-traps. Again for these traps we always have a pair of identically designed traps that are either green or yellow and hanging in the same tree.

The rare cases where this paired design was not respected was due to inevitable random problems on the field (typically traps detached, destroyed,... or more occasionally handling errors). This caused occasional missing values in the paired design. In essence, these types of problems are stochastic and could add a bit of noise in our data but no systematic bias.

As underlined by the reviewer, the design concerning the comparison between the models of traps is less systematic and has suffered more divergences from the initial "ideal" design that we had conceived. The main reasons are often practical constraints or problems that might diverge between the teams (e.g., certain trap models not available on time, more limited time for trapping, traps that were destroyed and that could not be replaced because our stock was depleted, repeated vandalism on some traps by passers-by, ...)

As recommended by the reviewer we have re-analyzed a subset of the data that is more homogeneous to evaluate how much this impacts the conclusions. We used only the trees and dates for which the complete set of traps used in each year was available (i.e. the lines that are neither blue nor grey in the reviewer’s attached Excel file). We see this as a way to evaluate the robustness of our conclusions. We also tested various statistical approaches when possible, e.g.: Poisson or Negative binomial models, dropping the bottle traps to avoid the comparison with the fan traps, and analyzing the data separately for both years. All these complementary analyses are available in the supplements, section 3.5.

The impact on the conclusions was very limited despite the fact that this approach dropped a considerable amount of data. This demonstrates that our conclusions are very robust to the incompleteness of the design or the statistical approach.

The main divergences between the different approaches concerned the multifunnel green traps. In all analyses, these traps tended to capture slightly fewer specimens than the MULTz (and to a lower extent than the Yellow Fan-Traps), but this difference can be statistically significant or not depending on the statistical approach. This is due to the fact that the size effect is small and the variation between sites is high which decreases the confidence in the estimates. In the paper we took a conservative approach, saying that these traps tend to capture less but that the differences were not significant.

The patterns for the species-level captures remained globally similar, and we can still observe the divergence of *A.biguttatus* which is more often caught by multifunnel green traps.

We hope that these considerable efforts to complement our data analysis will convince reviewer #1 of the robustness of our conclusions, which are firmly grounded in substantial evidence.

In the manuscript, the authors presented the results of the evaluation of different trap types and colours as well as presence of decoys on catches of the jewel beetles in different types of sites.

Introduction provides sufficient information on the current state of knowledge and motivation to initiate further studies.
Unfortunately, experimental design was not well prepared and/or organized/harmonized, thus causing data analyses complicated. Most of shortcomings of the experimental design are presented only in supporting file, but not in the main body of the manuscript, thus the current description of the methods makes an impression that the studies were well developed and conducted, while, in fact, there were many issues. The experimental design have to be described in details to make sure/clear that data were collected and analysed properly.

 My major concerns are:
 1. The period of trap exposure and frequency of inspections presented in line 129 do not correspond to what can be found in further description, Table 1 and file with raw data. Both time of exposure and frequency of inspections varied much depending on year, sites etc.

See the general answer. We have added a paragraph explaining that there are divergence between the final field design -and the planned one

Authors wrote that they used randomised block design, in which trees (blocks) were supposed to be nested in sites, but actual design was very unbalanced. E.g., in 2021, four types of traps were tested in 13 sites. In each site, sets of four trap types were supposed to be hung in the canopy of three trees, however two (Multifunnel green and MULTz yellow) of four trap types were hung in the canopy of two trees, instead of three. In Table 1, the design looks even more complicated: in Fagus sites (two sites) – the number of Multifunnel green traps should be 4 in total (2 sites x 2 traps), while there were only 2 traps; the number of MULTz yellow traps should be also 4, but there were 5. In Quercus sites one could expect 12 traps of Multifunnel, but there were 11. Similar issues were in 2022.

It also appeared that different types of traps within blocks were exposed for different periods, varying in 2021 in Fagus sites from 37 to 107 days, in Pyrus sites – from 38 to 101 days and in Quercus sites – from 91.7 to 100.5 days. Similar issues were in 2022. It is getting even worse, when one checks the file with raw data – there were very few cases when all tested trap types were exposed in the field simultaneously (in the same periods). The authors seem to neglect this and tried to standardise the sampling effort by calculating the catches per day and then multiplying by 90 days, however such approach cannot be used when different traps within a block were exposed in different periods, because flight activity of insects is not constant through the time. It is crucial to analyse data only for the period, during which all compared trap types in a block (a tree) were exposed in the field simultaneously. In the excel file attached to this review, the authors could see what data can be used (in transparent raws) or cannot be used (grey colour) for testing the effect of trap type. Data cannot be used because the number of captured beetles is missing for at least one trap type. Sites/raws in the blue colour can be skipped because of all zero values. In France, all tested trap types were exposed during the same period only in three sites (although green fan traps were hung on other tree than remaining traps) and the period was very short – from 29 June till 28 July. Only after appropriate selection of data, the authors could standardize the catches obtained in Belgium (usually covering period longer than 90 days) by calculating them for the period of 90 days. Data from France that can be included in analyses are available only from the period of 29 days, basically in July, when catches of Agrilus were already much lower than in May and June, thus standardizing them in the same way as the Belgian data would lead to great over/underestimation of catches, depending on Agrilus species.

See our main answer and the robustness analysis in our new version of the supplements (section 3.5)

2. Data from two years cannot be combined in one data set for the analysis, because there were two different trap sets tested, therefore bottle traps cannot be compared with fan traps. Data from two years have to be analysed separately.

The data of both years can be combined but we agree that bottle traps and fan-traps were never present in the same blocks nor in the same year. As a consequence, the paired comparison between both types of traps is taken into account by the mixed models and results in a much more uncertain estimation (higher standard errors). This is probably why the differences between bottle traps and yellow fan traps are not significant despite the relatively large size effect, and we cannot really conclude for these ones. This was explained in the supplements. We have now added a sentence in the caption to attract the attention of the reader on this point.

We also tested the impact of a separate analysis for both years and of dropping the bottle traps from the paired comparisons in our robustness analysis (see supplements section 3.5).

This had little impact on the general conclusions.

 3. Data from traps of the same type but with and without decoy cannot be summed (line 251), but have to be averaged – you compare trap type, not doubled trap type. The issue of trap size have to be included in the discussion. If you have mixed data (not divided depending on the presence of decoy) and you want to include them data into analyses (if the effect of decoy appeared not significant), you have to divide the results by 2.

The definition of what constitutes a “trap” is rather arbitrary. The principle of the “fan-trap” is a modular piece that can be assembled depending on the need. The traps “with decoys” are already a combination of a pair of modular pieces attached together back to back (see on the field pictures). Two pairs of modular pieces were hung on top of each other, one pair with decoys, the other pair without decoys. To us, it makes as much sense to sum the captures of these 4 modular elements as it makes sense to group the captures of the 12 funnels constituting the multifunnel traps, and the 8 modular elements constituting the MULTz traps (4 pairs of elements attach back to back). These are just modular elements hanging on top of each other at exactly the same place in the tree.

This has zero impact on our conclusion of the effect of the colour because the yellow and green fan traps are designed exactly in the same way. Of course, this has an impact on the comparison between trap types but the fact that different trap designs differ by their colour but also by their catching area and several other aspects is discussed at length in the discussion.

 4. Data for testing the effect of decoy on trap catches, effect of trap type on the number of species and probability of detecting each selected insect species have to be checked and carefully selected for analyses considering conditions mentioned in points 1-3.

Please see our general answer above and robustness analysis in the supplements

 5. The number of replications is not sufficient for creating models with many factors and variants/factor, particularly for models including insect species (with 7 species), e.g. trap_type*decoy*species and with more factors and interactions. I would suggest to analyse data for each selected insect species and year separately, using appropriate data (see point 1). Such approach will impose the use of data only from oak stands in the analyses. Testing the effect of any factor on combined catches of all insect species seems unreasonable – in addition to concerns mentioned above, the results will show rather the patterns characterising the most abundant insect species, while the effects on less abundant species will be hidden.

There are several different points raised here

i) If we do not have enough statistical power to test a general model with “trap_type*decoy*species”, we don’t see how we could have enough statistical power to analyse each species separately. It is a very common approach to first test whether the interaction is significant (i.e. with the maximum statistical power) and only if it is significant to explore further which species differ and how. For the decoys/no decoys tests, none of the interactions with species were significant so we simply reported this “test” and proposed a visual representation of the data in order to visualise the “absence” of interaction. For the comparison between trap types, the interactions with species were significant so we indeed proceeded to an analysis species by species (and by definition, we had enough statistical power).

ii) Concerning the interest in studying the total catches: a) we show that the total number of captures is strongly correlated to the number of species. So in a surveillance program where one wants to “detect” as many species as possible, maximizing the number of insects caught is an interesting objective in itself. b) of course, these results could be strongly influenced by the most abundant species. But we do not analyse only the total number of catches, we also perform the analysis species by species where there is evidence that the species differ (i.e. when the interactions with species are significant).

6. I would suggest to use data without log-transformation (pooled number of specimens of a certain species captured during appropriate period – see point 1, then standardized and rounded to integers) and GLMM with either Poisson or negative binomial distribution of residuals for testing the effect of trap type and sex or decoy and sex. When such data are used, you don’t need to account for differences in sampling effort as long as you use results for the period (dates) when all trap types in a block (on a tree) were exposed simultaneously.

It seems to me that you incorrectly specified the GLMM models (lines 226-229). By the way, if you use notation of random factors in the following way: (1|SiteID) + (1|TreeID), it would be useful to make a note in M&M that Tree ID was unique for each site.

We don’t see model specification in lines 226-229 and the notation “ (1|SiteID) + (1|TreeID)” was never used in the core of the paper, only in the supplements. The M&M clearly specifies that the Tree is nested inside the Site (lines 171-172).

Concerning the usage of Poisson or Negative Binomial models, we understand the suggestion to use these models typically used for count data. However, as explained in the M&M, the problem is that these models will work for some of the analyses but not for all because of convergence problems, unidentifiability of the estimated models, etc. When the Poisson or Negative Binomial do converge, they lead to similar conclusions to the Gaussian models. We already included an example at the end of section 3.1.1 of the supplements (for the decoys), and we have now included several other examples in the robustness analysis (sections 3.5.2 and 3.5.3.2). The Gaussian models have the following advantages/characteristics: 1) they work in all cases (--> easier to explain with a unified approach for all questions), 2) the residual plots show that the log-transformed data are very well approximated by a Gaussian model and finally 3) as we have a very reasonable amount of replicates, even slight discrepancies from the Gaussian distribution are of minor importance (cfr central limit theorem).

7. I am not sure why tree species was included in some models.

Because the *Fagus* or *Quercus* genus could have an influence on the results.

8. Probability of detection of different species by different trap types is worth to test probably only for most abundant species. The authors used model (Suppl. Material)
 glmer(Presence ~ Trap_Type*Species + (1|SiteID) + (1|TreeID),family = binomial),
 but it seems to be written in a wrong way. In binomial model, the dependent variable has to be presented as the number of traps with species presence divided by the total number of traps and the model has to include weights = total number of traps, i.e. the model (for each insect species separately) would have to look as following:
 glmer(number of traps with species presence/total number of traps ~ Trap_Type + (1|SiteID) + (1|TreeID), weights= total number of traps, data=data, family= binomial).

Our model specification is correct because we use a special case of the “Binomial” distribution called the “Bernoulli” distribution where the response is binary (presence/absence) rather than a proportion (number of success/number of trials). In the GLM context they are both specified through a “binomial” family (e.g. in function glmer or glm) and in the binary outcome case, the weights are the default value of 1 for all observations.

 9. Lines 392-393 – I am not sure why you analysed correlation between the number of species and the number of insects – there is no information on this point in M&M.

To see if the number of insects caught is a good proxy to determine which trap will capture the highest specific diversity, hence potentially detecting incipient non-native pest species (in a plant-health monitoring context). We didn’t mention the Pearson correlation in the M&M because it is so basic that we don’t see the point of mentioning that in the M&M and then again in the results. We consider that the Pearson correlation is a descriptive statistic as the mean, median, and standard deviation and that it would be overkill to describe such basic statistics in the M&M. Also the supplements and R code are always available if anybody wants to dig deeper.

 10. Results and Discussion will have to be rewritten after the proper analyses using carefully selected data are done.
As shown with the robustness analysis in the supplements (section 3.5), changing the subset of data or statistical approach has no impact on the conclusions

Besides major issues related to the experimental design and data analyses mentioned above, there are also other issues.
 11. In M&M, it is necessary to provide more information on:

1) forests/orchards in each site – age, density, species composition and stand structure (layers, presence of undergrowth/understory) (shorter description in the main body of the manuscript and more details – in Suppl.Info),

2) distance from a trunk where traps were hung – was it comparable for each trap hung in a crown,

3) approximate height of trap location above the ground and how traps were hung in the upper canopy (were all traps in each site hung there),

4) location of trees with traps –interior or edge of a forest/orchard. All this information could be important for understanding/interpreting the results, e.g. differences in species composition and abundance of buprestids in Belgium and France.

The traps were placed as high in the crown of the tree as possible, using a catapult or a very long telescopic arm (typically between 5 and 20 metres, depending on the height of the tree, and lower for the smaller Pyrus trees). The different trap models were grouped on the same tree and, where possible, on the same branch to make their catches as comparable as possible. We have added some details about this in the M&M (lines 141-148).

It would certainly be interesting to study the factors explaining differences in catch rates at the site level or at the tree level. However, it was not the aim of the current study, and it would require a different statistical design. For example, for the site level effect, one should probably use only one type of trap and deploy them on many different sites with a good variation in forest structure. For the tree level effect and trap position effect, one should replicate various positions and trees within the sites.

As it wasn’t the aim of our study, we didn’t collect detailed information on each tree and stand, and we don’t have access to this information retrospectively. This is not necessary to answer our questions: what is the impact of the presence of decoys, what is the impact of trap colour and/or design on the catch rates? We have placed the different trap designs in the same trees, and the variation between sites and trees is captured as random effects (which are typically used to take into account this kind of heterogeneity). The traps were, for example, at different distances from the trunk (obviously when they are on the same branch), but the order and exact placement were random. So even if this had an effect, it would just add some noise to our dataset.

Note also that even if one would be able to demonstrate that the optimal distance of the trap from the trunk is 187cm, in practice, it would be very difficult to place the traps exactly at that distance for two reasons: 1) you are strongly constrained by the branches available on the field and 2) it is very difficult to place exactly where you want a trap at 10-20 m height when using a catapult or a telescopic arm.

12. It is necessary to explain why decoys of A. planipennis (alien species not present in Europe, except Ukraine and western part of Russia) was used for testing the effect of decoy on catches of Agrilus species native to Europe, particularly if one could expect species-specific response.

It is explained in the Introduction (lines 101-107) that decoys can elicit interspecific responses (ref 57, 58 of the revised manuscript), notably for *A. biguttatus* which is more attracted by EAB decoy than by conspecific according to the literature. In a previous study, the use of EAB as a decoy increased the catches of traps for European Agrilus species (ref 46). Furthermore, it would not have been possible to use decoys from different species because it would have increased the number of replicates in unmanageable ways. In the end, we had to choose one species, *A.planipennis* was easy to obtain in large quantities and had already proven to be effective even on European species. The possibility of obtaining the decoys in large quantities is also important if we want the protocol to be used routinely in surveillance programs. 3D-printed decoys could be a solution for that too.

Of course, the results might be different with decoys from other species, but this should be tested in a different study.

 13. Latin names of insects have to be followed by their authors.
This does not seem to be a requirement of the journal Plos One for papers not focussing on taxonomy. There are plenty of papers published by this journal without the authors of Latin names. We have added a table in the supplements (section 2.2) with the author names retrieved from the GBIF backbone taxonomy (with the package taxize).

14. Why the authors focused on buprestids in broad-leaved species? What about the species in conifer forests, e.g. Phaenops cyanea? There was not a word in introduction or in discussion about those species, while the species that are not green might provide some insights into the relationships between colour of trap and the colour of insect and/or host tree.

The reason is double: 1) you have to stop somewhere, you cannot test all species. 2) adding more sites with a different habitat increases even more the difficulty of getting a perfectly balanced design…

For your information, one of the teams involved in this paper is currently testing different trap models in Pine stands, precisely to target *Phaenops cyanea*. But in a different year, with slightly different protocols, etc.

 15. It would be more logical to describe all analyses in one chapter called e.g. Statistical analyses, starting from description of data preparation, then what was tested and how (type of models, model structure, data selection, etc.) and then programs/scripts used for analyses.

We organised the Data analysis section in order to minimize the need for tedious repetitions. So we start from the general information that is valid for most analyses, and then we go into the details. The fact that we publicly provide our R script with a literate programming approach (mixing code, explanations and code output) is a way to document with the highest level of precision possible all the steps that were realized in a sequential way.

 16. In Discussion, I would make a point about the importance of colour and size of different buprestid species on catches in traps of certain colours and with decoys of certain size/colour. This might be useful in explaining low catches of A. sinuatus.
We have added a discussion about the colour of *A.sinuatus* (lines 478-480)

17. I wonder why neither in Results, not in Discussion no attention was paid to close match of main peaks of reflectance in fan-trap yellow and multifunnel green traps that could explain their similarity in catches. I would suggest to move the description of the results of reflectance measurements from M&M to Results and add one figure in the main manuscript to show this match.

The spectral analysis was just a way for us to objectively describe the colour of our traps because we could not find any standardized reference colour for our yellow traps. Despite the proximity of the reflectance peak, the spectra of the green and yellow traps are clearly different (this was already stated in the manuscript, lines 230 – 233 of the revised manuscript).

 18. Lines 477–481 – is it about the results presented in the manuscript or about the results discussed earlier (ref. 46)?

Lines 477-479 (initial version of the ms) are related to reference 46 and 479-481 is about future research where it would be interesting to disentangle in which conditions lures are working.

 19. Files in Supporting Materials, particularly the way how raw data are presented, are not really helpful in understanding what data were used for each analysis. There are much more data than are directly related to the manuscript. Closer look at the raw data showed that there are major issues with experimental design and proper selection of data for analyses is crucial.

The R code is provided so that anyone can reproduce exactly our analyses and know exactly which data has been used for each analysis. We have also added some more information about this in the supplementary report

 20. Figures – the authors should avoid presenting non-significant results in Figures, while all the significant results should be well demonstrated, either in figures or in tables.
We beg to disagree. One of our questions was to test for the effect of decoys. If we get negative results (i.e. non-significant differences), it is important to present them clearly to avoid contributing to the plague of publication bias (drawer effect). The fact that the differences are not significant is clearly stated in the main text and in the figure legend, so that the reader is not misled. The figures presented in the paper are a visual summary of the main conclusions of the paper.

21. I could not find the information about the contribution of each author.

We have fulfilled this information during the submission process. We do not know if this information is available to the reviewers during the review process (it is indicated on PLOS ONE website that “*Contributions will be published with the final article*”.

 22. Explain all abbreviations, e.g. PTFE, GLMM etc. at the first place you introduce them. It would be better to avoid using NB for specifying some information.
Done

There are some grammatical and style issues:
 Line 30 – write “two-week basis” instead of “2-week basis”; numbers 1-9 usually are written in words.

Done
 Line 60 – delete dot after “traps” Done
 Line 138 – MULTz was yellow, not yellow-green Done
 Line 144 and 182 – “with PTFE for dry surface” – I am not sure what it means,

Meaning of the first abbreviation added
 Line 143 and 181 – “attached them by back-to-back couples” - I am not sure what it means

One modular piece was attached to another one by their posterior face. Fig 1B should make that clear (reference to the figure added in the second instance)
 Line 145 – “in the upper canopy” – pictures in Fig. 1 suggest that the traps were not hung in upper canopy – please, provide clear information on where and how the traps were hung (see point 11).

Replaced by “as high as possible in the crown”
 Line 151 – please write more clearly – was each trap type on a different tree or there were some combinations? Data in supplementary file shows that all four trap types were tested in 4 of 5 sites and only in the period from 9 August till 16 September.

In pear orchards, the trees were too small to hang all the traps in the exact same tree. But the traps were hung in trees next to each other and were considered as being part of the same sublocation (called TreeID) nested inside the site.
 Line 161 – I presume that the number of samples means the number of inspections of each trap. Am I write?

Yes, one sample = the insects collected by one trap during one sampling period
 Line 179 – “the” first and second layer

Reviewed by a native speaker : “a” is fine
 Line 210–212 – need corrections – a) residual plots are not used for checking “the conditions of application of the model”; b) “the choice of transformations” – what does it mean?, 3) “(61, 62); see Supplements)” – do not help in understanding what exactly you did, and please correct parentheses.

We follow the approach proposed by the two references cited, i.e. the excellent books of John Fox and Alain Zuur, which consist of evaluating whether the chosen model is a good approximation of the data based on the visual examination of several residual plots (in contrast for example with the usage of Normality or Heteroscedasticity tests that are arguably not a good approach).

Fox J. An R and S Plus companion to applied regression. 1st ed. Thousand Oaks, California, USA: Sage Publications, Inc; 2002. 328 p.

Zuur AF, Ieno EN, Smith GM. Analysing ecological data. 1st ed. New York, USA: Springer-Verlag; 2007. 672 p.

Line 215–216 – please correct style ¬ a) treatment doesn’t catch insects, 2) “specimen of the particular response variable being analysed” – what does it mean?

It means that if the response is for example, Agrilus biguttatus, we remove the trees in which this species has never been captured during the whole season to avoid including locations where the species is not present and where the traps cannot be compared. This corresponds to the blue lines of the reviewer’s provided excel file when working with the total number of individuals caught but adapted according to the response being used in each model.

We modified the formulation and added an example to make this clearer..
 Line 529–532 – 1) structure of sentence has to be corrected, 2) colour closer to – “closer” is not a correct term in this case; you could use “similar”, “corresponds to”, “reflects” or something similar.

Corrected (and using the conditional as these are just hypotheses)

**Reviewer #2:** The manuscript is well written. It is about an extensive series of 36 field experiments in different vegetations to test trapping devices suitable for jewel beetle monitoring. Further, the manuscript includes developing a novel trap device for jewel beetles. Lab measurements and a sufficient review of the up-to-date literature also support the work. Their result helps the understanding of the state of the art of jewel beetle monitoring devices and prove statistical conclusions that I myself had a gut feeling of but had no sufficient data set to prove like the abundance of jewel beetles in different habitats, the importance of the tree on which the monitoring traps are placed, which demonstrates how important trap rotation is within experiments with jewel beetles. Altogether the present work adds to our knowledge in the field of trapping Agrilus jewel beetles.

We thank the reviewer for her/his interest in our research and the very positive comments.

**Reviewer #3:** Overview: This work uses a few different trap designs to test for their ability to capture Agrilus beetles on different tree types in European forests and orchards. The authors collect useful data that indicates how certain species may be caught using these different trap designs. While the data collected is interesting and useful, the presentation of the data falls far short of presenting the information to the reader in an understandable and relevant form.

We thank the reviewer for the critical analysis of our study.

First, there is far too much focus on visual decoys, with little understanding evident as to how they might work in trapping systems. The decoy trap studies referenced by previous authors, make it clear that decoys only work in branch-based traps where the traps consist of small leaf-sized surfaces that are wrapped around terminal branches and placed directly within the live foliage of the trees. Those previous studies showing that decoys can work in these situations, as well as other studies have always found that decoys do not work well in larger stand-alone traps such as funnel traps or prism traps. Thus, the lack of an effect of decoys in this study as the authors designed the experiment is entirely expected and not worthy of such in depth discussion as they dedicate to the topic. It would be better to simply mentioned that the decoys were used on some of the traps and present the statistical support that they did not have a significant effect, without dedicating an entire Figure to this point (Fig 3)

We do not reach the same conclusions when examining the published literature (see table below). There are indeed several studies reporting higher captures associated with decoys on sticky-branch or sticky-leaf traps (eg [1–4]) however these studies do not explicitly compare these results with larger stand-alone traps. We found a single study [5] investigating decoy effect on both sticky-leaf traps placed within the foliage and small-sized stand-alone sticky cards of limited dimensions (10x10 cm and 10x25 cm). The authors argue that sticky-leaf traps performed better than stand-alone sticky cards when adjusting for trap area (although they did not formally compare both trap types), but they also showed that the decoys increased the captures on the stand-alone sticky cards. Only one published study [6] studied the effect of decoys on large white (although in this case the decoys are large silhouette/picture; see table) and purple stand-alone (not hung into trees) sticky traps (40x60 cm) and concluded that a large number of decoys could increase the capture rates of *A. planipennis*/*Agrilus* spp. Rodriguez-Soana et al. [7] also showed that decoys directly pinned on the trunk could elicit mating behaviour, also suggesting that attractivity to decoy is not restricted to branch/foliage environment. So we don’t see clear evidence from the published literature that the decoys could not work on large stand-alone traps. Furthermore, none of these studies have tested a decoy effect on non-sticky traps. To our knowledge, this has only been tested in one very recent study (still in press) from Santoiemma et al. who reported no significant effects of *Agrilus* decoys on abundance or species richness of *Agrilus* spp.captured in non-sticky green intercept panel traps. That study found a positive effect of *A. bilineatus* decoys on catch of only 1 of 25 species tested, *Agrilus geminatus*, and found negative effects of *A. sulcicollis* and *A. laticornis* decoys on catch of *A. sulcicollis* and *A. hastulifer*, respectively. We have added this information in the manuscript (lines 497-501) and the study of Santoiemma et al. (under reference 60).

Finally we also strongly believe that it is very important to report clearly negative results as ours in order to limit the increasing problems of publication bias leading to an over-representation of false positive results and the reproducibility crisis in science [9,10]. This means also that even if decoys had already been tested on stand-alone non-sticky traps, replications are always valuable, particularly in ecology where the environmental and design conditions are rarely strictly identical. In addition, several of the studies mentioned concern *A.planipennis* or the American fauna and evaluating various trapping design approaches in other contexts remains interesting.

| **Target species** | **Decoy** | **Nb. of decoys/trap** | **Type of trap** | **Trap location** | **Results** | **Source** |
| --- | --- | --- | --- | --- | --- | --- |
| *A. planipennis* | Dead EAB | 1 | Sticky-leaf traps | in the canopy | Presence of decoy increases captures in sticky-leaf traps (but no statistical tests) and on coloured cards (no preference for yellow or blue -> captures pooled). No formal comparison between sticky-leaf and coloured cards (authors attempt to compare the mean captures, adjusting for trap area and conclude that sticky leaf traps are far more attractive). | [5] |
| *A. planipennis* | Dead EAB | 4/8 | Yellow/Blue small sticky cards (10x25 cm) | hung to outer branches |  |  |
| *A. cyanescens* | Dead EAB | 4/8 | Yellow/Blue small sticky cards (10x25 cm) | hung to outer branches | Presence of decoy increases captures on blue sticky cards |  |
| *A. subcinctus* | Dead EAB | 4/8 | Yellow/Blue small sticky cards (10x25 cm) | hung to outer branches | No colour/decoy effect |  |
| *A. planipennis* | Dead EAB | 1 | Sticky-leaf traps | in the canopy | Presence of decoy increases captures | [8] |
|  |  | 1 | Green small sticky cards (10x10 cm) | in the canopy | Presence of decoy increases captures but sticky-leaf > green cards | |
| European oak-associated Agrilus | Dead EAB | 1 | Sticky-leaf traps |  | Green branch traps + EAB > Green branch traps > all other trap | [1] |
|  |  | 1 | White/green/violet/Leaf-covered Sticky-branch traps (2*5x9 cm) | |  |  |
| Buprestid beetles | Enlarged purple Agrilus-shaped silhouette (15x55 cm) | 1 | White sticky panel (40x60 cm) | above the ground (with no tree nearby) | No significant differences between traps | [6] |
|  | Enlarged green Agrilus-shaped silhouette (15x55 cm) | 1 | White sticky panel (40x60 cm) | above the ground (with no tree nearby) |  |  |
|  | Enlarged A. planipennis picture (15x55 cm) | 1 | White sticky panel (40x60 cm) | above the ground (with no tree nearby) |  |  |
|  | Dead EAB | 1 | Purple sticky panel traps (40x60 cm) | above the ground (with no tree nearby) |  |  |
|  | Dead EAB | 25 | Purple sticky panel traps (40x60 cm) | above the ground (with no tree nearby) |  |  |
| *Agrilus* spp. |  |  | same design as above for the same study |  | More capture with 25 decoys than with no decoy (for females and for males + females) |  |
| *A. planipennis* |  |  | same design as above for the same study |  | More capture with 25 decoys than with 1 (only for males + females) or with no decoy (for females and for males + females) |  |
| *A. planipennis* | Dead EAB | 1 | Green Sticky-branch traps (2*5x9 cm) |  | Presence of decoy (dead EAB or 3D-printed model) increases captures | [2] |
|  | 3D-printed decoy | 1 | Green Sticky-branch traps (2*5x9 cm) |  |  |  |
| *A. planipennis* | Dead EAB | 1 | Green Sticky-branch traps (2*5x9 cm) | in the canopy | Increased captures of males for traps with decoy and with (Z)-3hexenol-plus-lactone (interaction between odour and decoy) | [3] |
| *A. planipennis* | Dead EAB | 1 | Green Sticky-branch traps (2*5x9 cm) | in the canopy | Increased captures of males for traps in healthy trees | [4] |

Bibliography associated to previous comment

1.	Domingue MJ, Imrei Z, Lelito JP, Muskovits J, Janik G, Csóka G, et al. Trapping of European buprestid beetles in oak forests using visual and olfactory cues. Entomol Exp Appl. 2013;148: 116–129. doi:10.1111/eea.12083

2.	Domingue MJ, Pulsifer DP, Lakhtakia A, Berkebile J, Steiner KC, Lelito JP, et al. Detecting emerald ash borers (Agrilus planipennis) using branch traps baited with 3D-printed beetle decoys. J Pest Sci. 2015;88: 267–279. doi:10.1007/s10340-014-0598-y

3.	Domingue MJ, Andreadis SS, Silk PJ, Ryall KL, Baker TC. Interaction of visual and chemical cues in promoting attraction of *Agrilus planipennis*. J Chem Ecol. 2016;42: 490–496. doi:10.1007/s10886-016-0706-y

4.	Domingue MJ, Berkebile J, Steiner K, Hall LP, Cloonan KR, Lance D, et al. Host condition effects upon Agrilus planipennis (Coleoptera: Buprestidae) captures on decoy-baited branch traps. Eur J Entomol. 2016;113: 438–445. doi:10.14411/eje.2016.057

5.	Lelito JP, Fraser I, Mastro VC, Tumlinson JH, Baker TC. Novel visual-cue-based sticky traps for monitoring of emerald ash borers, *Agrilus planipennis* (Col., Buprestidae). J Appl Entomol. 2008;132: 668–674. doi:10.1111/j.1439-0418.2008.01308.x

6.	Petrice TR, Haack RA, Poland TM. Attraction of *Agrilus planipennis* (Coleoptera: Buprestidae) and other buprestids to sticky traps of various colors and shapes. Gt Lakes Entomol. 2013;46: 13–30.

7.	Rodriguez-Soana CR, Miller JR, Poland TM, Kuhn TM, Otis GW, Turk T, et al. Behaviors of Adult *Agrilus Planipennis* (Coleoptera: Buprestidae). Gt Lakes Entomol. 2018;40. doi:10.22543/0090-0222.2168

8.	Domingue MJ, Lelito JP, Fraser I, Mastro VC, Tumlinson JH, Baker TC. Visual and chemical cues affecting the detection rate of the emerald ash borer in sticky traps: Visual and chemical cues for *Agrilus planipennis* traps. J Appl Entomol. 2013;137: 77–87. doi:10.1111/j.1439-0418.2012.01737.x

9.	Mogil JS, Macleod MR. No publication without confirmation. Nat News. 2017;542: 409. doi:10.1038/542409a

10.	Møller AP, Jennions MD. Testing and adjusting for publication bias. Trends Ecol Evol. 2001;16: 580–586. doi:10.1016/S0169-5347(01)02235-2

It would be a better use of the data, to more present how the different tree types affected the species of insect trapped. The authors do mention that tree type did have a statistically significant effect on total numbers caught. However, it would be very interesting to see which species were caught on the different tree types. The authors focus instead on country rather than tree species to describe the species as they did in figure 2. However, a figure similar to figure 2 showing the different tree species and the distribution of species caught would be more interesting. It would be too much to present in figure form, but a Table listing each combination of tree and trap type with the corresponding numbers of each species caught would also be interesting and helpful for understanding which traps might be best to use for each species.

As requested by the reviewer, we have added a figure illustrating the species composition by tree species in the supplements section 2.2. (and a reference to this figure in the legend of Figure 2 in the paper). Determining which trap type is the best for each species is only feasible for the most abundant species that are all living on oak trees and was done in the trap type comparison (results are illustrated in Figure 5). For the other tree species (Pear trees, and Poplars) some details can be found in the supplements (sections 4.3 and 4.4) but the conclusions (e.g. for Poplars) that can be derived from the data are relatively limited due to the small number of individuals (and sites).

The effect of color is only tested for one trap type (the fan type) This is helpful as well, but as the authors mention, the yellow is better for most species, but not all, so it should not be implied strongly that the yellow traps are optimal. Agrilus biguttatus is a much more economically destructive pest than many of the others described, so it’s preference for green funnel traps may be highly important to a monitoring or surveillance program.

We agree with the reviewer and modified our discussion accordingly. First, we clearly indicate (lines 545-548) that *A. biguttatus* appears to be attracted by the multifunnel green as a whole and not just by the green colour since green fan-traps capture few specimens (and not more than yellow fan-traps). Second, we highlight the fact that multifunnel green is more appropriate to monitor A. biguttatus which is the most damaging species to oak trees in Europe and that the choice of the monitoring equipment should be weighted according to the economic importance of the target species (lines 595-599). We also added that we found a strong correlation between the total number of individuals captured per trap and the number of species. So depending on the objective of the surveillance program one might prefer one type of trap or another (eg targeting specifically *Agrilus biguttatus*, or targetting a broad range of species).

More specific comments on particular sections:
 Title: Very specific conclusions are presented in the title, which are not strongly supported by the data and/or based on a less than optimal design. A broader title without such specific conclusions would be more appropriate, such as “A comparison of trap designs for capturing Agrilus species in multiple forest and orchard settings in Europe”

Our title shortly summarises the main results of our study: we found no decoy effect for the trap types tested (i.e. bottle- and fan-traps) and, for a same trap design (the fan-trap), the fluorescent yellow colour globally performed better than green ones. Of course, this is valid only in the context tested in this study and more nuanced results are described in the abstract/article but this title allows the reader to quickly grasp and remember the main results of our study.

Ln 107: Add here that that decoy-baited traps have only been shown to be effective when designed to be placed on branches within the living foliage. 21, 46 and also Domingue et al., Journal of Pest Science 88, 267-279

This is true but it has never been tested (or at least published) in other contexts (thus not proven to be ineffective with other trap types/placement). We added that “*So far, the decoy effect on capture effectiveness has only been tested with sticky traps, mostly of small size and placed on branches within the living foliage*” with appropriate references.

Ln 108-116: This description of the need for traps to be placed high in the canopy and easily accessible also fits in to the issue of decoys. Because decoys only work in branch traps that can be reached and serviced easily from the ground on low lying branches, they are less convenient. They also only work in sticky designs. Therefore, it would be better to justify your study examining the role of decoys on these multifunnel and fan traps to be sure they are not effective in these situations as the previous literature would also predict that they would not be.

Please see above our previous responses to comments on the effectiveness of decoys.

Figure 1 caption: what type of site, with what tree species is being pictured here in each panel?

We added that traps are hung in an oak tree for panel A. For fan-traps (panel B), they are not hung in a tree, they were hand-held during the picture for illustrative purposes.

Figure 2: Does this include pool all the tree species? It should be explicitly mentioned in the caption.

It indeed includes all specimens captured from all tree species. We added this information to the caption.

Figure 4: It is not necessary to include the word “mixed here” You had demonstrated in figure 3 that pooling the traps with decoys is not an issue. You also do not use the designation in Figure 5. It just confuses the reader to have it here.

We have removed the word “mixed” from all graphs

Ln 441: It would be worth mentioning that branch trap designs might be worth investigating in orchard settings such as this (ref 21&46) if these other trap designs are failing.

We added that sticky traps (e.g. branch traps used in ref 21) may be more appropriate to this species if the failure to capture *A. sinuatus* is linked to its ability to escape non-sticky traps (lines 483-486).

Ln 451-481: the authors spend a disproportionate amount of time discussing previous decoy studies and questioning the statistical validity of these studies and minor methodological issues. They are neglecting to discuss the much more important consideration that the trap design used for branch traps were much different than any of the trap designs used in this study.
 The branch traps place the decoys directly in among the leaves of the tree, which is likely the crucial factor in providing enough visual and olfactory stimuli to allow the decoys to work. In a stand-alone trap placed in trees, hanging far from the branches it seems likely that the mating behavior exhibited by the males in response to the decoys simply cannot be reproduced.

We thank the reviewer for this additional hypothesis and we added it to explain the potential impact of trap position and environment on decoy effect (lines 517-525).

The authors also question the validity of the statistics of the branch trap studies as a result of the failure to account for experiment-wise error. However, the main finding was based on a pooling of all species, which was highly significant. Questioning the significance of decoys for each species here seems to be wondering well outside the scope of this study and what conclusions can be made about decoys from these authors’ results.

Please note that the statistical discussion was only our very last argument and was a comment on the only study concerning the effect of decoys on European species. We believe that underlining the fact that the patterns detected in this unique study have not been replicated in other studies or even within this study (i.e. in the second “olfactory” experiment the decoy effects are not significant even on the results from the pooling of all species) is a clear call for more tests on European species. Note that this is not a critique of this excellent study. False positive happens (even with p-value correction and even if there was no publication bias, they should happen ~ 5% of the time…). We however modified our discussion on the results of Domingue et al. (reference 46) and removed the part on statistics.

Ln 482-521: The authors make an interesting point about the economic and usability advantages of fan traps. Some quantification of the costs of each trap type should be included.

We added cost quantification based on the offers we received for commercial multifunnels (including shipping fees).

**Reviewer #4:** This manuscript reports on field trapping experiments in Belgium and France to compare efficacy of different trap types and colors with or without dead Agrilus planipennis decoys for capturing buprestid species including known pests of oak, beech, and pear trees. Overall the paper is well-written and it provides new information that will improve monitoring surveys for buprestid beetles that are among the most damaging forest pests and highly successful invaders. The experiments were well designed and were replicated across multiple sites per year in Belgium and France, the results are clearly presented and the discussion and conclusions are supported by the data. I don’t have any major concerns with the paper and believe it is suitable for publication with some minor revisions.

We thank the reviewer for the time spent on our manuscript and for his/her positive comments on our study.

1. The abstract (lines 30-31) states that over 2 years, 382 traps were deployed across 46 sites in Belgium and France. However, in the methods it states that 4 trap designs were compared at 13 Belgium sites in 2021 (line 132) with 3 replicates (line 145) per site (13*4*3=156 traps) and 6 trap designs were compared at 23 sites located in Belgium or France (line 166) in 2022 with 2 replicates (line 188) per site (6*23*2=276) for a total of 36 sites (13+23) and 432 (156+276) traps – which does not agree with the totals in the abstract.

It represents the differences between the initial (theoretical) sampling design and the field reality. Due to multiple reasons, some samples could not be included (trap broken/lost, issue with trap installation, shorter monitoring period, …). This was not clearly stated in the initial manuscript and we have added a short paragraph at the end of the MM - Monitoring design section (lines 215-220) to clarify this point. Importantly, these discrepancies had no effect on the general conclusions of our analyses (please see supplements section 3.5 and general response to reviewer#1 for details).

2. It would be helpful to include a little more detail in the methods. Were traps baited with any lures? How were samples collected from the collecting pots – were the contents strained and placed in bags and how were samples stored until counted? Since all trap types were hung in the same tree for each replicate, could there have been any cross attraction or interference? Were the traps hung in different aspects/cardinal directions of the tree canopy? Were trap types rotated around the tree canopy to different aspect positions between collection periods? Canopy position/aspect and condition (open/edge, proximity to adjacent trees) can impact attraction of different species.

We added information on the placement of the traps and collection and sorting of samples at the beginning of the MM - Monitoring design section (lines 141-153). The traps were indeed hung in the same tree and, where possible, on the same branch. This could induce interference between traps (at least for long range attraction because traps were hung at least 80-100 cm away) but this was the only way to expose the traps to the most similar conditions. We tried as much as possible to place traps on well spaced and sun exposed branches (but this was not always on the south facing side). Trap types were placed at random on each tree and the position of each trap was kept until the end of the field season (it takes a lot of time to properly hang so many traps in the canopy). Canopy aspect and tree condition were not recorded (but we never hung traps in solitary trees).

3. Spectral data for the traps were measured (line 191-203) but are not discussed in the results or discussion. How does the spectral reflectance of the different traps compare to electro-retinogram studies (if available) or leaf color?

The spectral analysis was just a way for us to objectively describe the colour of our traps because we could not find any standardized reference colour for our yellow traps. According to Domingue et al. (2016), the reflectance peaks of the yellow traps are a bit closer to the maximum reflectance of *A. angustulus* males and leaves of *Quercus alba* (N-American oak species, thus not a natural host of *A. angustulus*, *A. biguttatus* and *A. sulcicollis*), while the maximum reflectance of green traps are closer to the reflectance of wild-type green *A. planipennis*. Reflectance peaks of both *A. biguttatus* and *A. sulcicollis* fall quite far from our traps

Domingue MJ, Lelito JP, Myrick AJ, Csóka G, Szőcs L, Imrei Z, Baker TC. 2016. Differences in spectral selectivity between stages of visually-guided mating approaches in a buprestid beetle. Journal of Experimental Biology:jeb.137885. DOI: 10.1242/jeb.137885.

4. Why were trap catches standardized per 90 day period instead of the 2 week sampling period between collections?

Because we used the sum of catches from all periods and that 90 days was close to the median of the total number of catching days (so it was the closest to the true value of most sites). This also provides larger numbers that are easier to read on graphs (basically you don’t need to pay attention to the number after the decimal separator). Of course, multiplying by 90 days or by 14 days changes absolutely nothing in terms of statistical analysis.

5. Consider including statistics for the statement on line 266-267 that year was not included in the model because it seemed to have little effect. The statistics for the non-significant effect of year are provided at line 339.

We added a reference to the model using the total number of individuals across all species in which the year effect was not significant.

6. What multiple comparison procedure was used to compare all pairwise comparisons (line 270).

We used the default single-step method of the “multcomp” package as specified in the next line (271 in the initial manuscript). This is also totally transparent from the R code provided that will be made publicly available.

7. Any idea why yellow/green color might be less attractive to A sinuatus in pear orchards, given that pears are yellowish green in color? (line 441)

If traps are attractive because they mimic mate colours (rather than foliage for feeding), the bronze colouration of *A. sinuatus* may explain a lower attractiveness toward green/yellow traps. The low attractiveness of yellow/green traps could also be due to the general conditions (exposure, contrast) that are very different between orchards and forest stands rather than to colour only. We added these hypotheses lines 478-483. Furthermore, the green/yellow colours may actually be attractive to *A. sinuatus* but this species may be better able to escape the traps (recent trials with yellow sticky traps were more successful in catching). This is also stated in the manuscript (lines 483-486).

8. Does A. sinuatus fly at lower heights in pear orchards that typically have shorter trees than forests? (line 446)

Host plants of *A. sinuatus* are of small height (*Pyrus communis*, *Crataegus* spp., *Sorbus* spp.) and we assume that specimens in pear orchards fly at tree height since they effectively feed and reproduce on cultivated pear trees.

9. What about testing decoys of the target species rather than A. planipennis? (line 451)

This could certainly be interesting, but we could not test that in the framework of this study. We used *A.planipennis* because it was easy to obtain in large quantities and it has been shown in the literature to also attract European species. The possibility of obtaining the decoys in large quantities is an important criterion if the trapping system has to be deployed at a large scale for a surveillance program. Some teams have tested 3D printed decoys that could be a good solution if it works.

10. Recommend changing “profitable” to “cost effective” (line 518) profitable implies the traps will generate income (make money)

Done

11. Was there a balanced sex ratio for all species (line 522)
We tested a model including the species and the sex (see supplements section 3.2.5.1). In that model the sex:species interaction is significant (p = 0.005) meaning that the sex ratio varies between species. However, none of the interactions with the trap_type effect was significant (meaning that if the sex ratio was different between species, it remained comparable between trap types for a given species). The differences are relatively subtle and can be seen in Figure 28 in the supplements (section 3.2.5.1). We caught slightly more males in some species, but for *A.graminis*, we captured slightly more females. Note that the sex main effect is not significant, meaning that if we ignore the species the differences in sex ratio between species cancel each other. We have modified this paragraph accordingly.

12. Recommend changing “pay off” to “increase detection and captures” or “optimize detection and captures” (line 539) - again "pay off" suggests monetary gain.
Done

13. Consider dropping the word “interestingly” in several places - it is subjective.

Done

14. Consider changing passive to active voice in several places (e.g, change “for the monitoring of buprestids” to “for monitoring buprestids”).

Done

15. Drop “To summarize” at the beginning of the conclusions section. It is redundant since a conclusion is clearly a summary.

Done

16. Could break up a few long paragrphs in the discussion that cover multiple topics. For instance the paragraph starting at line 425 could be broken at line 438 to separate discussion of the impact of tree and site characteristics form the impact of color and height.

Done
