# Supplementary material for: Enhancing Buprestidae monitoring in Europe: Trap catches increase with a fluorescent yellow colour but not with the presence of decoys
Source: PLoS One. 2024 Jul 18;19(7):e0307397. doi: 10.1371/journal.pone.0307397 (PMC11257278; doi:10.1371/journal.pone.0307397)
Supplement: Attachment — Submitted filename: PlosOne-review_revised1.docx [file pone.0307397.s004.docx]

Dear Authors,

Unfortunately, you haven't addressed most of my comments sufficiently, particularly the most crucial points 1-4, therefore I sustain them and will not be able to accept the manuscript as long as data are not managed, analyzed and presented correctly.

Generally speaking, you admitted that your experimental design in reality was far from what should be, but you haven't changed much in the main body of the manuscript. Writing a few sentences about the flaws in your experiments doesn't solve the problem of incorrect data management and analysis.

1. Yes, current statistical methods allows for some flexibility in data balance etc., however much depends on a type of experimental design. In your study, you intended to apply randomized complete block design (with each treatment, i.e. compared trap types in this case, present in each block at the same time of trap exposure), but the raw data show that it was not fulfilled in many sites. Why it happened is not actually important. You could use incomplete block design, but it has its own rules and actually was not planned by you a priori. So, I repeat what I already wrote in my first review, you have to use only those data that fit to the design and the hypotheses you tested, so that they can be analyzed properly and the results can be used for a well-grounded inference. The data look quite well for testing the effect of decoy (but see points 3 and 4), but not for testing the effect of trap type (see table 1 below). When testing the effect of decoy, I would suggest to do so for each trap type separately (single model for each trap type – this will give you flexibility in selecting datasets).
2. Second very important issue is that you cannot extrapolate data from shorter periods to longer, unless the difference is a couple of days, by just multiplying the available catches/day by 90 days, because insect activity is not constant throughout the season. By doing so, you use artificial data, either underestimated or overestimated, that could lead to incorrect inferences.
3. No, you cannot include data from two years in one model, when you changed experimental design (trap types tested) in the second year (which can be seen in the table below).
4. You have extremely or very low catches in traps set in *Fagus*, *Pyrus* and *Populus*, thus a detection of differences between treatments is rather impossible. In addition, species composition of beetles differ much, that actually could be expected. Therefore, I suggest just to simply describe the total catches and catches of each insect species (without making any statistical testing due to very low numbers) in different years for *Fagus*, *Pyrus* and *Populus*, while to conduct statistical analysis of catches in *Quercus* trees only. It will make your paper more clear.
5. The issue in comment 4 is also related to my question in the first review on the reason of including tree species in some models – with so low catches and differences in insect species composition, there is no sense to do so.

All the above issues could lead to the problems with model’s convergence. For the same reason, I would avoid constructing too complex models. For testing the effect of trap type on the catches of selected insect species, just do the analyses for each species with sufficiently high catches separately.

6. Summing two traps of the same type – no, it is no the same as using 12 funnels in 12-funnel trap, because your initial traps used in the experiments were single fan traps and single bottle traps. After stating no effect of decoy, you decided to sum the results and present them as it was a single trap (you did not write in your figures that there were double fan traps and double bottle traps).You have to present the results per single trap.
7. When asking for details about characteristics of forests/orchards, I meant that they could be useful in interpreting the results, e.g. presence of one or another insect species, particularly for understanding such huge difference in catches between sites.
8. When asking for details about trap position (proximity to stem or upper part of crown), I did not mean exact distance in cm, but the fact that some traps could be closer to leaves than others on branches and crown parts, which could affect trap visibility/attractivity to insects. This issue was also pointed out by another reviewer. The trap placement (randomization of order/distances) should be described in details in the manuscript.
9. When asking for tree locations in forests/orchards, I meant that traps placed in trees growing in the forest interior would be more shaded than in trees growing at the edge. This could affect the catches of your target insects considering their preference for sun-exposed places.
10. Not only myself, but also some other reviewers pointed out that there is no need to present non-significant results in figures – it is sufficient to describe them in respective chapter and mention in abstract, so that none will miss them. You will need space for presenting the results after you consider points 1-5.

I will not review the results and discussion until the analyses are done properly and on correctly selected datasets and the results are presented in the main body of the manuscript (not in supplementary materials). Abstract also has to be rewritten, as it describes the study as it was well-designed experiment with all traps checked every 2 weeks, while it was not so. In addition, you state that you tested the effect of trap color, while I could find this info in M&M.
